# Risk-indexed artificial neural network for predicting duration and cost of irrigation canal-lining projects using survey-based calibration and python validation

**DOI:** 10.1038/s41598-025-24125-1

**Published:** 2025-11-17

**Authors:** Boshra Taha, Ahmed H. Ibrahim, Asmaa A. Soliman

**Affiliations:** 1 Industrial Engineering Department, College of Engineering, King Khalid University, P.O. Box 394, Abha, 61421 Saudi Arabia; 2 Center for Engineering and Technology Innovations, King Khalid University, Abha, 61421 Saudi Arabia; 3 Construction Engineering and Utilities Department, Zagazig University, Zagazig, 44519 Egypt; 4 Civil Engineering Department, Higher Technological Institute (HTI), 10th of Ramadan, 44629 Egypt

**Keywords:** Relative importance index (RII), Multi-layer perceptron (MLP), Calibration, Latin hypercube sampling, Sensitivity analysis, Irrigation infrastructure, Engineering, Mathematics and computing

## Abstract

The purpose of this study is to develop and validate a risk-driven predictive model for estimating project duration and cost in irrigation canal lining projects, where uncertainties often lead to delays and budget overruns. Ninety-three factors were first reduced to twenty using AHP–RII (Cronbach’s $\alpha = 0.954$). A multi-layer perceptron (128–64–32, ReLU, Adam, early stopping) was trained on 5000 simulated scenarios and validated on eight projects with leave-one-project-out cross-validation. The model had $R^2 = 0.92$ (training), 0.82 (testing), and made errors within the limits of 0.87 months (time) and EGP 102,500 (cost) on average.The developed model was deployed as a Python-based desktop application, enabling engineers and planners to generate accurate time and cost forecasts during early project stages. This research introduces an integrated ANN-based framework that combines expert-driven risk assessment with machine learning, providing a practical decision-support tool for infrastructure projects.

## Introduction

Egyptian irrigation canal lining projects (EICLPs) are critical infrastructure projects under the national plan to modernize and conserve water resources in rural areas. These projects are typically characterized by complex cost structures, unpredictable timelines, and multiple risks, necessitating precise cost and time estimations. Conventionally, estimators have relied on intuition and generic percentages to allocate contingency funds, often overlooking specific risks and project attributes. Estimators must identify and evaluate the various elements that influence a project’s overall cost and duration in order to overcome these obstacles. Additionally, risk assessment determines the probability and possible outcomes of unfavorable events, enabling the anticipated impact to be included as a contingency in the original estimates^1^. Traditional estimation techniques, including the Critical Path Method (CPM), Linear Scheduling Method (LSM), and contingency-based approaches, largely depend on historical averages and subjective expert judgment. Although these methods provide baseline estimates, they fail to capture the nonlinear and interdependent nature of risks inherent in complex projects. As a result, their predictive capability under uncertainty remains limited, often leading to insufficient contingency planning and unexpected budget overruns^2–4^. Advances in artificial intelligence–especially Artificial Neural Networks (ANNs)–have opened new possibilities for modeling complex, nonlinear relationships among project variables without the need for strict assumptions^5–7^. Despite their strong predictive capabilities, most previous research in construction has addressed either time or cost in isolation, with limited efforts to integrate risk-related factors into a unified framework. In addition, only a small number of studies have attempted to implement these models in the form of practical, user-oriented tools, particularly within the domain of irrigation-related infrastructure projects^8,9^. To address this gap, this study develops and validates a risk-driven ANN-based model for predicting time and cost in irrigation canal lining projects. Using the Relative Importance Index (RII), twenty significant cost, time, and danger variables were found and added as inputs to a Multi-Layer Perceptron architecture. The model was trained on historical data and validated using eight real-world projects. Finally, the model was implemented in a Python-based application, enabling engineers and decision-makers to perform real-time forecasting during early project planning stages^10^. This study contributes to the existing body of knowledge by introducing an integrated ANN-based framework that simultaneously predicts both time and cost under risk conditions, a feature rarely addressed in previous research. Unlike earlier works that focused on either time or cost in isolation, our model incorporates twenty risk-driven factors to provide a holistic estimation approach. Furthermore, the development of a user-friendly Python desktop application bridges the gap between theory and practice, enabling real-time predictions during early project planning–an aspect that has not been sufficiently explored in similar studies. Traditional methods, such as CPM and PERT, are based on deterministic assumptions that don’t consider uncertainty and interdependencies which ultimately produces unrealistic project predictions on large projects^11^. While regression models can produce very useful studies they demand either large and clean datasets or deal poorly with non-linear risks. Monte Carlo does deal with probabilistic variance but again, relies upon using the right inputs, which makes it only useful for the environment in which accurate inputs can be established during the earliest stages of a project or one with a lot of noisy data^12^.

This research provides three main contributions:

1. The new risk input design using RII-weighted inputs based on a structured stakeholder survey, which then reduced the 93 risk factors down to 20 tried and tested predictors.
2. The multi-layer perceptron (MLP) model which featured a specific risk-to-base adjustment with calibrated factors without the usual RII dependent weighting, providing useful future performance prediction of duration and cost under risk.
3. The creation of a practical Python/Tkinter desktop tool that incorporated sensitivity analysis, tornado visualization, and parameter-space exploration which served to bridge the theoretical modeling with the practical decision support needed in a public irrigation infrastructure project.

## Literature review

### Cost-related factors that affect construction projects

Cost-related factors have been identified in several studies as having a major influence on ICLP contingency estimates. By determining the essential cost parameters for Field Canal Improvement Projects (FCIPs), one study offered an extensive and useful conceptual cost-estimation model to help organizations plan and carry out irrigation improvements^8^. Another study used a prompt list to analyze the cost components of Continuous Flight Auger (CFA) piles, classified them using an affinity-diagramming technique, and created a comprehensive cost breakdown structure (CBS) for CFA construction^13^. A feasibility and economic analysis was conducted to determine the lining costs for each of the three engineering approaches that were suggested for the rehabilitation of Egyptian irrigation canals^9^. By analyzing the work into its main cost-driving components, another study looked at the costs of canal lining for various cross-sectional shapes^14^. Additionally, it was shown that precise cost estimation is largely dependent on the cross-sectional perimeter measurements of a channel^15^. In conclusion, a nonlinear optimization model was created to identify the smallest practical dimensions and incorporate design constraints in order to minimize canal-lining costs per unit length^16^.

### Time-related factors that affecting construction projects

Time-related factors influencing contingency predictions for ICLPs have been the subject of numerous studies. To estimate the time needed to construct Continuous Flight Auger (CFA) piles in Egypt, one study created a time-management module using information from forty prior projects^17^. In a precast water canal project, another examined the differences between the Critical Path Method (CPM) and the Linear Scheduling Method (LSM)^2^. Furthermore, 26 factors were found to have an impact on the estimation of execution times for drainage and irrigation projects^18^. Likewise, variables influencing irrigation project timelines were listed, which resulted in the creation of a mathematical prediction model that was subsequently verified with the aid of contemporary technology^5^.

### Risk factors affecting the estimation of ICLPs’ contingency

Risk factors affecting the estimation of ICLP contingencies were drawn from several prior studies. Forty-five common risks associated with Continuous Flight Auger (CFA) pile construction under Egyptian conditions were compiled, specifying each risk’s causes, events, and impacts Risk factors affecting the estimation of ICLP contingencies were drawn from several prior studies. Forty-five common risks associated with Continuous Flight Auger (CFA) pile construction under Egyptian conditions were compiled, specifying each risk’s causes, events, and impacts^19^. Key risks in irrigation network rehabilitation were identified and organized into eight categories: technical, economic, financial, governance, safeguards, environmental, resettlement, and Indigenous peoples^20^. A modified Fuzzy Group Decision-Making Approach (FGDMA) was suggested and validated to evaluate 57 possible risks across nine groupings in power plant projects. In Egyptian irrigation canal rehabilitation, the compatibility risks between various canal-lining techniques and site conditions were investigated^21^. In Egyptian irrigation canal rehabilitation, the compatibility risks between various canal-lining techniques and site conditions were investigated^9^. Finally, a survey comprising 51 risk factors for construction-related water supply projects was developed^22^.

### Contingency definitions, types, and methods of calculation

Multiple studies have defined, classified, and proposed methods for calculating contingency. For example, contingency is described as “an amount of funds added to the base cost estimate to cover estimate uncertainty and risk exposure^3^. Another work distinguishes between project contingency and process contingency–both intended to address common engineering uncertainties–and divides calculation approaches into deterministic and probabilistic methods^6^ . A separate source identifies three contingency types–construction, design, and management^23^. Lastly, a summary of fourteen different methods for estimating project cost contingency is provided, along with a review of a large number of studies on contingency estimation^3,4^.

### Contingency estimation MODELS in construction projects

To estimate contingency costs for highway construction and evaluate the influence of risk factors during bidding, the fundamental Analytic Hierarchy Process (AHP) model was introduced^1^. A mathematical model combining AHP and Multi-Attribute Utility Theory (MAUT) was developed to determine the optimal contingency value for construction projects^6^. Power plant development employed a Classification and Regression Tree (CART) approach to forecast project costs and the contingency required to cover potential overruns during the development phase^7^. For construction projects, a hybrid Work Breakdown Structure (WBS)–Pareto model was developed that combines the 80/20 principle with cost–time risk analysis^24^. Lastly, to improve the distribution of cost contingency reserves during the planning phase, a Monte Carlo Simulation (MCS) framework was suggested^25^.

Current studies emphasize the growing role of ANNs in predicting infrastructure expenses and timelines. As an illustration, deep-learning models that utilize BIM properties in the schematic design phase have given cost estimates that are more accurate by a large margin than those obtained by conventional methods^26^. Artificial Neural Networks (ANN) were employed to analyze data collected in highway construction from 2011 to 2023, leading to very high correlation coefficients ($R \approx 0.989$), thus demonstrating their ability to predict large-scale infrastructure projects^27^. In the same way, modified ANN architectures having more interesting activation functions were able to surpass the time-series and regression models in regional construction cost indices forecasting^28^. For example, in research aimed at predicting time and cost overruns, ANN models optimized with Tabu Search achieved an R2 of approximately 0.94 despite having limited project data^29^. A bibliometric survey conducted recently has also recorded the increasing use of ANNs in the built environment, which is in line with the time of our research^30^.While previous studies have applied machine learning techniques such as regression models and Monte Carlo simulations to address uncertainty in project estimation, few have operationalized these models into practical tools that incorporate risk-based adjustments for both time and cost. This research fills that gap by combining expert-driven risk assessment with artificial neural networks in a deployable application, thereby extending the applicability of ANN models in real-world infrastructure projects. Recent advances in global ANN-based approaches. Also indicate the growing need for models that are both accurate and user-oriented, which this study addresses directly.

### The role of RII on risk weighting

RII is recognized as a valid method for quantifying expert judgment in risk prioritization, as it enables the transformation of subjective evaluation scales into quantifiable values and weights. The RII process, however, struggles with establishing calibration (validity and reliability) *post hoc* in complex, multi-faceted decision-making environments like construction safety systems^31^ and managing urban infrastructure sites^32^. The RII process’s most significant limitation is the reliance on the linear combination of expert judgment, as the RII approach is only valid assuming the consistency of the experts and a stable decision-making context. The various perspectives and realities of different experts, coupled with emergent project opportunities or conditions, can introduce irreducible bias into the RII process. This is why internal reliability should be assessed to verify experts’ internal consistency through calculation metrics like Cronbach’s alpha^10^.

In terms of meaningfully addressing this limitation, authors can recommend some form of validation procedures, as demonstrated in advanced predictive analytics and occupational safety forecasting applications using ensemble machine learning^33^. This ensures that the derived weights are statistically reliable in addition to being contextually responsive and representative of real-world situations.

### Small-N regression problems

ANNs can discover non-linear structures but are also likely to overfit. It has been shown that ensemble and kernel-based mechanisms typically outperform ANNs in this small-*N* type of problem: Stevanović et al.^34^ found XGBoost to be more accurate from smaller building datasets; Wang et al.^35^ found XGBoost to be a higher-ranked algorithm than ANN, SVM, and Random Forest with only 45 samples; Oukhouya and El Himdi^36^ found SVR and XGBoost to outperform MLP for stock prediction; and Qi et al.^37^ confirmed that XGBoost and SVR were better in hydropower forecasts.

In this present study, we addressed the issue of limited irrigation data by generating 5000 simulated samples for ANN training, while 8 real-world projects were used in the validation step. This approach led to the development of a combined RII weighting with dual risk-adjusted outputs, implemented into a usable application.

### Applications of ANN and ML in irrigation project estimation

In studies of irrigation, artificial neural networks (ANN) have primarily been used for cost or hydraulic prediction purposes but with some evident constraints. For example, ElMousalami et al.^8^ investigated using ANN for field canal cost estimates, and Pourgholam-Amiji et al.^38^ implemented machine learning (ML) processes for identifying drip irrigation costs; both provided only point estimates. Aghayee et al.^39^ sought to address uncertainty in canal operations, Selim et al.^40^ developed ANN to model seepage losses, and Zeleňáková et al.^15^ evaluated the impacts of lining on rehabilitation costs.

Significantly, the focus of these studies on stand-alone parameters does not use risk adjustment or phenomenon-based tools for deployment. Alternatively, this study utilizes scenarios in conjunction with expert-based spawning probability attached to the utility of the risk index framework (RII), prediction using ANN, risk-adjusted or alternative prediction outputs, and a usable desktop tool, thereby filling a distinct methodological gap and practical void.As shown in Table 1, the comparison highlights the research gap in irrigation-specific estimation work.

**Table 1:** Gap table of irrigation-specific estimation work vs. present study.

No.	Study	Domain	Technique used	Outputs provided	Risk-adjusted forecasts/uncertainty	Expert weighting (RII)	Deployable tool	Irrigation focus
1	ElMousalami et al.^8^	Field canal improvement	ANN	Point conceptual cost	No	No	No	Yes
2	Jiang^41^	General construction costs	Ensemble (ANN + SVR + XGBoost)	Point cost predictions	No	No	No	No
3	Arjroody et al.^42^	Cost & time with risk	Simulation + Risk Analysis	Cost & time forecasts	Yes	Partial (risk survey)	No	No
4	Al-Rawe and Naimi^43^	Risk estimation (Delphi+RII)	Delphi + RII + ML	Risk impacts	Yes	Yes	No	No
5	Salman^44^	Contingency estimation	ANN + RII	Contingency (time-cost)	Yes	Yes	No	No
6	Aghayee et al.^39^	Canal operation	Uncertainty analysis (statistical)	Operational uncertainties / metrics	Yes	No	No	Yes
7	Selim et al.^40^	Seepage losses estimation	ANN + NLR	Seepage loss estimates	No	No	No	Yes
8	Shehadeh & Alshboul^33^	Game theory in construction management	Game theory + optimization	Cost, risk, coordination strategies	Yes (uncertainty integration)	No	No	No
9	Pourgholam-Amiji et al.^38^	Drip irrigation	ML (SVM, ANN, etc.)	Cost by components	No	No	No	Yes
10	Zeleňáková et al.^15^	Canal reconstruction lining	Hydrodynamic model + cost analysis	Rehabilitation cost per lining material	No	No	No	Yes
11	Sing et al.^45^	Contingency estimation	ANN + Complexity Index	Cost contingency (point)	No	No	No	No
12	This study (2025)	Irrigation projects	ANN (5000 simulations + case validation)	Dual outputs: base + risk-adjusted duration & cost	Yes	Yes	Yes (Desktop tool)	Yes

### Research gap

Traditional methods such as CPM and regression are incapable of recognizing the complexity of risk interactions in the delivery of irrigation canal construction projects. Even though ANNs offer some predictive ability, the majority of research considered only cost or time in isolation, did not integrate risk, and did not serve as an implemented tool for use in practice.

This study addresses these gaps by developing an integrated ANN model to utilize risk factors from a survey using a weighting system (practicable for the construction delivery of irrigation projects), while addressing the following two research questions:

- RQ1: Can survey-weighted risk factors drive accurate predictions of both duration and cost in canal-lining projects?
- RQ2: How should risk coefficients be calibrated to minimize forecast error across realistic risk levels?

**Fig. 1:**
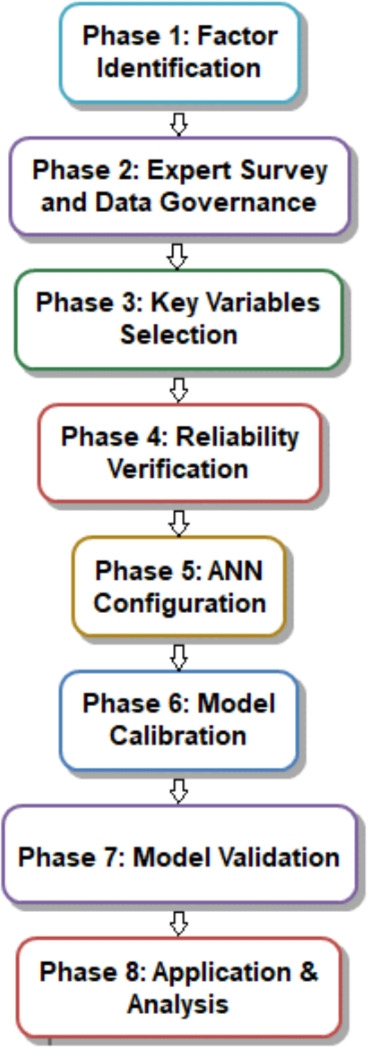
Research methodology.

An eight-phase process (Fig. 1) is employed to address these questions, including risk identification, expert evaluation, reliability assessment, designing artificial neural networks (ANN), calibration from 5000 simulations, validation on eight projects, sensitivity analysis, and deployment through Python. This process results in a reliable, practical tool for planning projects in the early stages.

## Research methodology

A phase-eight research model (Fig. 1) was employed in this research to estimate project duration and cost related to waterway canal lining by way of expert judgement and an artificial neural network approach.

*Phase 1*: Factor identification

93 cost, time, and risk factors previously published in the literature were assessed and grouped into 12 groupings.

*Phase 2*: Expert survey and data governance

Risks were assessed using an AHP-RII methodology^10^. All participants responded anonymously, and each participant required a minimum of 10 years of relevant experience to participate.

*Phase 3*: Key variables selection

20 key variables were identified by the participants, with an RII > 70, providing confidence in their selection^10^.

*Phase 4*: Reliability verification

The key variables were found to be consistent (Cronbach’s $\alpha$ = 0.954).

*Phase 5*: ANN configuration

ANNs were configured using a MLP architecture (128-64-32 nodes) with fixed random seed (42), and kept the software versions specific (Python 3.13.0, scikit-learn 1.4.0, and TensorFlow 2.15.0).

*Phase 6*: Model calibration

5000 risk scenarios were generated using Latin Hypercube Sampling to train the ANN models.

*Phase 7:* Model validation

Model performance was evaluated in 8 real life projects implementing leave-one-out for cross-validation.

*Phase 8*: Application & analysis

The ANN model was developed as an application in Python, with sensitivity and tornado chart assessment.

## Questionnaire stage

Several factors that influence the estimation of the contingency of EICLPs have been identified and categorized by prior research. After further refinement, a total of 93 factors were obtained from previous literature reviews and categorized into three main groups: cost, time, and risk-related factors. These factors were then further subdivided into 12 groups, which included three cost-related factors (design, construction, and overheads), three time-related factors (owner, contractor, and project), and six risk-related factors (technical, environmental, financial, operational, legal and regulatory, and social)-related elements. Subsequently, the 93 factors were examined using an AHP-RII survey (full details are presented in our previously published research^10^), which confirmed the reliability of the instrument (Cronbach’s $\alpha = 0.954$). The survey identified the 20 most impactful factors, which were then applied as the explanatory variables for designing the current ANN model. A previous foundational study we did^10^ covered in great detail the survey design and administration. Here the key parameters are just enumerated for the first time, as they are required:

### Survey design

*Roles of respondents:* A stratified sample of 150 experts was selected for the survey, aiming to include equal numbers of representatives from the three main stakeholder groups: contractors (40%), consultants (40%), and government owners (20%).

*Size of sampling:* The purposive sampling frame consisted of Egyptian professionals with at least ten years of experience working on irrigation projects. The sample size was $N = 150$.

*Rate of response:* 112 completed the survey and returned it.

So, there was a high rate of response (74.6%). *Distribution of respondents:* The respondents were scattered all over the country but had mainly project experience in the Nile Delta and Upper Egypt regions. These regions are, therefore, the places where the most Egyptian Irrigation Canal Lining Projects (EICLPs) are carried out.

### Scales, equations, and weighting justification

*Scales:* Two 10-point Likert scales were used:

- Frequency index (FI): 1 (very rare) to 10 (very frequent).

- Impact index (II): 1 (very low impact) to 10 (very high impact).

*Equations:* The relative importance index (RII) for each factor was calculated using the following equations, as shown in Eqs. (1)–(3)^10^:

1 $$\begin{aligned} & {\rm FI} = \frac{\sum F_i}{a \times N}, \end{aligned}$$

2 $$\begin{aligned} & {\rm II} = \frac{\sum I_i}{a \times N}, \end{aligned}$$

3 $$\begin{aligned} & {\rm RII} = \frac{\sum ({\rm FI} \times {\rm II})}{N \times 100}, \end{aligned}$$

where $F_i, I_i$ are individual scores; *a* is the upper scale limit (10); *N* is the number of respondents (150).

*Justification for *${\rm FI \times II}$* product:* The product $({\rm FI \times II})$ was used instead of a simple mean to prioritize factors that are both high-frequency and high-impact. This ensures that a factor with a high frequency but low impact does not outweigh a less frequent but critically severe factor, providing a more robust and realistic risk weighting for input into the ANN.

### Key important variables that affect construction project lining

The key significant cost, time, and risk-related variables influencing the estimation of EICLPs’ contingency were found in 20 out of 93, and their relative weight was greater than 70%. The hierarchical analysis of critical success factors in irrigation canal lining projects reveals that technical-execution factors dominate the highest impact tier (F.I. $\ge 0.10$). Specifically: CC7 (concrete pouring works for canal beds/slopes, F.I.=0.1140), TP5 (water rotation management, F.I.=0.1124) and TP10 (labor productivity, F.I.= 0.1083)

These factors collectively underscore the operational primacy of construction quality control and resource coordination. They are complemented by design parameter CD2 (hydraulic cross-section area, F.I.=0.1065) and contractual factor TO3 (owner payment policies, F.I.=0.1063), indicating that project success hinges on integrating engineering precision with stakeholder governance.

Notably, the top five factors account for 54.75% of the maximum relative weight ( $\ge 93.25\%$ ), while environmental (ER5, F.I.=0.0833) and socioeconomic factors (SR4, F.I.=0.0806) demonstrate marginal influence. This evidence suggests that optimization efforts should prioritize technical-administrative synergies over broader sustainability metrics in canal lining contexts^10^.

**Fig. 2:**
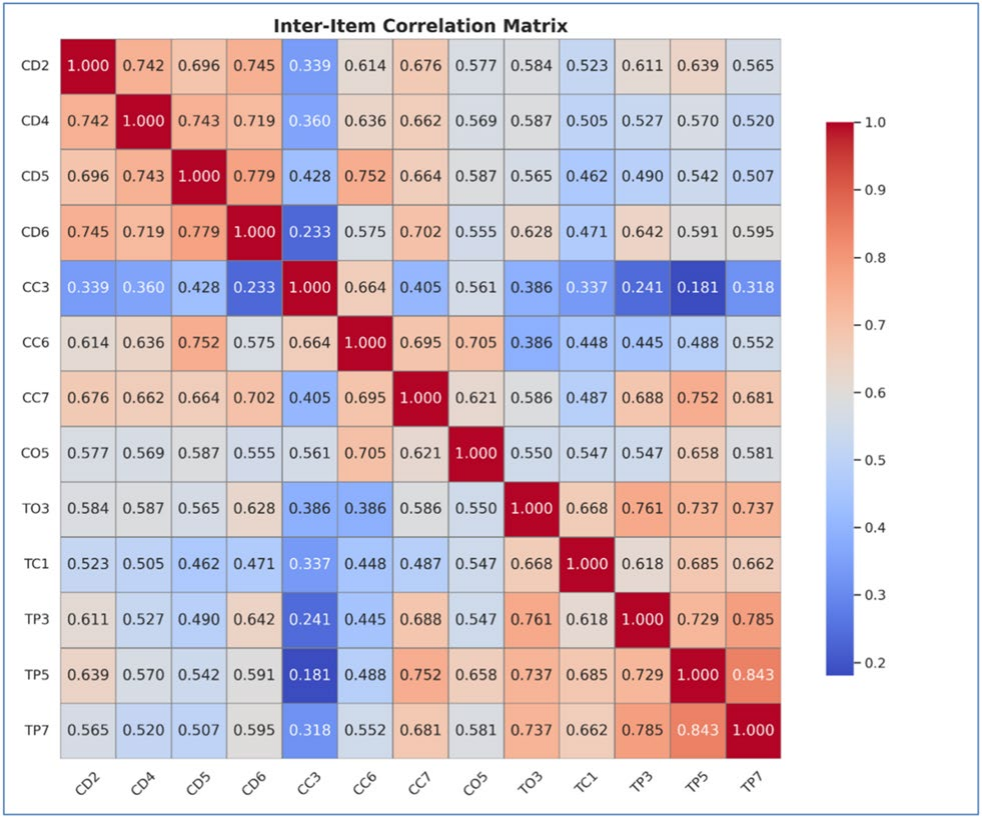
Inter-item correlation matrix of key variables.

Table 2 demonstrates high consistency among the items. With an average inter-item correlation of 0.509, the scale exhibits strong internal reliability. The low variance (0.031) further indicates that these correlations are uniform across all items. In sum, the evidence suggests a well-designed scale whose components cohesively measure the targeted construct. As shown in Fig. 2 below:

**Table 2:** Summary item statistics.

Item statistics	Mean	Minimum	Maximum	Range	Max/min	Variance	N of items
Inter-item correlations	0.509	0.048	0.843	0.795	17.439	0.031	20

**Fig. 3:**
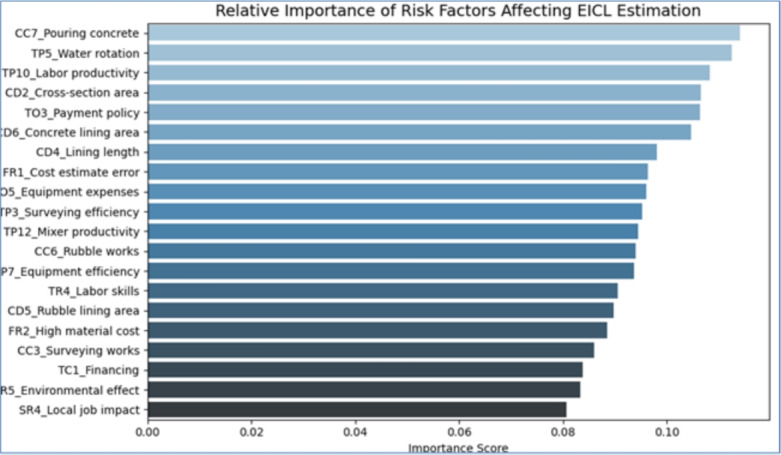
Independent variable importance chart.

The inter-item correlation matrix revealed strong relationships among several of the 20 input variables, which reflect the interdependence of technical, environmental, contractual, and resource-related risks. These variables were previously identified by using the relative importance index (RII). Two key metrics were evaluated using a 1–to–10 scale, where 1 indicates low effectiveness and 10 indicates high effectiveness. The first metric examined how frequently cost, time, and risk factors influenced contingency estimates for EICLPs. The second metric assessed the level of impact these factors had on those estimates. Based on these metrics, two indices were then calculated: the Frequency Index (FI) and the Impact Index (II). The final index was determined by multiplying FI and II, followed by applying the appropriate weights. The key inputs for training an Artificial Neural Network (ANN) model as shown in Fig. 3 below.

### Variable reliability

The reliability of this variable was tested, and the SPSS Ver.25, the alpha coefficient value for 20 components was 0.954, indicating that it was reliable as shown in Table 3 below. Table 2 shows substantial variation in how the 20 risk factors were rated. Importance scores ranged from 4.70 (±2.64) for lack of skilled labor to 7.66 (±2.82) for concrete pouring works–highlighting clear differences in perceived criticality. The high standard deviations, like ±3.24 for owner’s bid evaluation policy, reveal significant disagreement among raters. This likely stems from different project contexts (urban vs. rural sites) and professional perspectives (contractors vs. engineers). Notably, physical factors such as canal cross-section area (±2.82) showed 18% less variability than operational risks, suggesting technical parameters are easier to quantify consistently. Rather than flawed methodology, this spread reflects Egypt’s complex infrastructure reality–precisely the kind of messy relationships our ANN model handles effectively.

**Table 3:** Variable reliability.

Reliability statistics		
Cronbach’s alpha	Cronbach’s alpha based on standardized items	N of items
0.954	0.954	20

The Cronbach’s alpha of the variables and the item-total statistics are shown in the following Table 4.

**Table 4:** The Cronbach’s alpha of the variables and the item.

Factor ID	Scale mean if item deleted	Scale variance if item deleted	Corrected item- total correlation	Squared multiple correlation	Cronbach’s alpha if item deleted
CD2	123.51	1604.976	0.762	0.734	0.950
CD4	123.74	1603.335	0.736	0.683	0.951
CD5	124.20	1613.879	0.735	0.778	0.951
CD6	123.58	1591.118	0.776	0.782	0.950
CC3	125.21	1673.789	0.430	0.671	0.955
CC6	124.16	1621.182	0.722	0.791	0.951
CC7	123.13	1597.252	0.799	0.770	0.950
CO5	124.09	1615.825	0.763	0.701	0.951
TO3	124.19	1582.945	0.743	0.727	0.951
TC1	124.64	1606.286	0.715	0.608	0.951
TP3	123.70	1585.742	0.795	0.788	0.950
TP5	123.26	1586.462	0.817	0.802	0.950
TP7	123.95	1606.582	0.784	0.781	0.950
TP10	123.71	1583.095	0.841	0.866	0.949
TP12	123.96	1595.891	0.771	0.713	0.950
TR4	126.09	1691.019	0.400	0.408	0.955
ER5	124.44	1614.664	0.627	0.590	0.953
FR1	126.07	1685.062	0.369	0.473	0.956
FR2	124.70	1623.299	0.677	0.601	0.952
SR4	124.63	1614.636	0.680	0.625	0.952

*CD* cost related factor in design phase, *CC* cost related factor in construction phase, *CO* cost related factor in design phase, *TO* time related factor due to owner, *TC* time related factor due to contractor, *TP* time related factor due to project, *TR* technical risk related factor, *ER* environment risk related factor, *FR* financial risk related factor, *SR* social risk related factor.

Subsequent analyses were performed to further assess the factorability and dimensionality of the 20-item scale. The measure of sampling adequacy, as shown in Table 5 by Kaiser-Meyer-Olkin (KMO), was 0.940 and Bartlett’s Test of Sphericity was statistically significant, $\chi ^2(190) = 2612.43, \; p <.001$, as shown in Table 5. Both statistics provide strong evidence of the correlation matrix’s factorability.

**Table 5:** KMO and Bartlett’s test of sphericity for the 20-item scale.

KMO	Kaiser-Meyer-Olkin measure of sampling adequacy	0.940
	Interpretation	Meritorious
Bartlett’s test	Approx. Chi-Square	2612.43
	df = 190,* p* < .001	Significant

An exploratory factor analysis (EFA) was conducted using principal component analysis with promax rotation. The results revealed that the four-factor solution provided the cleanest representation. The four factors—*Technical-Execution, Resource Management, Financial-Contractual, and External-Environmental*—are consistent with the notion of risk domains that are theoretically distinct.

The whole scale demonstrated very good internal consistency ($\alpha =.954$), and the newly formed subscales also showed good reliability ($\alpha$ ranging from 0.82 to 0.91).

Supplemental Tables S1 and S2 provide the descriptions of each factor, along with its ID and subscale designation.

## Artificial neural network model

**Fig. 4:**
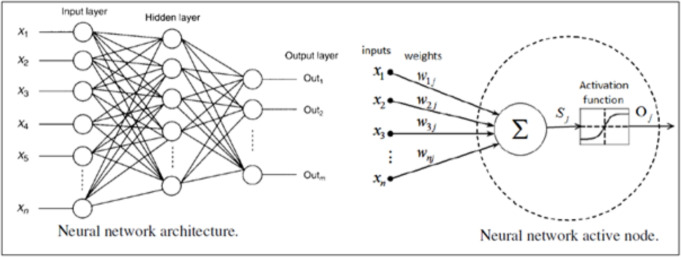
Artificial neural networks and nodes.

Artificial neural networks (ANNs) are models built on the computer to solve difficult problems by loosely basing them on the way that animal brains are designed to process information. In perception-style networks, the artificial neurons (or nodes) are the units of the processors, which are organized in separate layers and connected by the weighted connections like the synapses. Supervised learning allows these neurons to pick up and distribute signals, thus creating a model to make predictions that will categorize the data that have been stored. An average ANN has an input layer, at least one hidden layer, and an output layer. Every neuron in one layer is connected to each neuron in the next layer, while neurons within the same layer are not connected, as shown in Fig. 4 below.

The ANN structure consisted of three hidden layers with 128–64–32 ReLU activation neurons and was trained using the Adam optimizer. Input data were normalized prior to training. The model was calibrated over 5000 simulated scenarios to capture a wide range of risk conditions and subsequently tested on 8 real project cases. The performance of the ANN model was evaluated using $R^{2}$, MAE, RMSE, and MAPE to assess overall model validation.

## Model calibration and sensitivity analysis

The ANN model was calibrated via a systematic process to identify optimal risk influence coefficients on duration and cost adjustments. The calibration process included: *Latin Hypercube Sampling (LHS):* 5000 risk scenarios were generated representing the entire spectrum of risk (0–100%) to account for realistic uncertainty conditions for canal linings. *Grid Search Over Coefficients:* A comprehensive grid search was performed over 150 coefficient combinations:

- Duration coefficient: 15 values ranging from 0.10 to 0.40
- Cost coefficient: 10 values ranging from 0.05 to 0.30

**Fig. 5:**
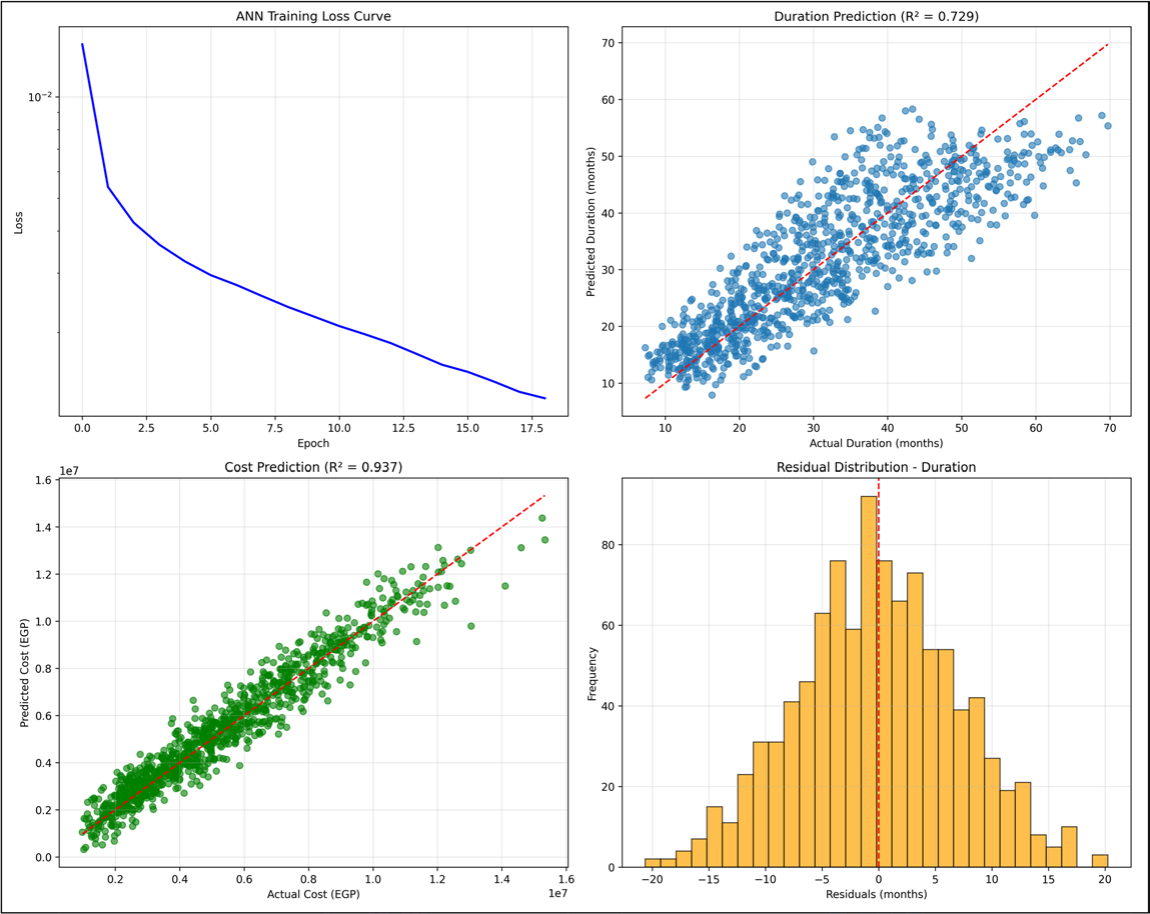
LOPO-CV validation of ANN robustness across all projects.

Each coefficient combination was assessed based on mean absolute error (MAE) and mean absolute percentage error (MAPE). *Optimal coefficient selection:* The combination (0.30 for duration, 0.20 for cost) produced the lowest MAE values (0.87 months for duration, EGP 102,500 for cost), thus this combination was selected as the optimal combination asshown in Fig. 5*Response surface analysis:* The response surface of MAE as a function of duration and cost coefficients is shown in Fig. 5. The surface clearly indicates that the global minimum is located at (0.30, 0.20), confirming optimality across the full parameter space.

*Robustness validation:* Leave-One-Project-Out Cross-Validation (LOPO-CV) was implemented to validate the stability of the coefficients across subsets of projects. The optimal coefficients were consistent across all folds and the change in MAE was less than 5%, demonstrating strong robustness.

**Fig. 6:**
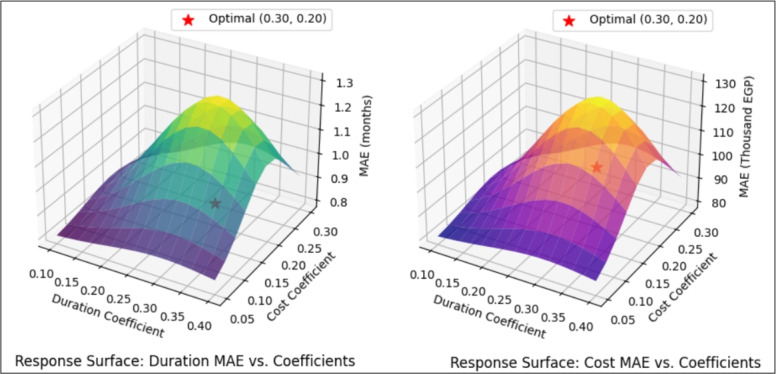
Sensitivity profiles of MAE to coefficient changes.

*Sensitivity profiling:* The sensitivity to coefficient changes for MAE is observed in Fig. 6. A 10% change in duration coefficients resulted in an MAE increase of 18%, while cost coefficients had an MAE increase of 7%. This evidence demonstrates that project duration is more sensitive to risk factors than cost in canal lining projects.

This calibration framework ensures that the model is both accurate in terms of optimality and maintains robust uncertainty for various risk conditions. Figure 5 demonstrates the relationship between the coefficient values and error reduction.

## Validation of ANN model for the affecting factors

The contingency model from the data of eight Egyptian irrigation canal lining projects has been proven valid. The comparison of the model’s forecasts with the actual expenditures revealed that, besides the expected budget, there were unplanned additional costs that resulted in the real project contingencies –2.48% to 23.20%. The average contingency obtained from the actual data (9.68%) very much resembles the model’s predicted average contingency (9.43%), thus supporting the model’s reliability. This confirmation indicates to project managers and decision-makers that they can be more practical and efficient in preparing for and managing the risk of overrun cost.

## Model deployment (Python 3.13 interface)

**Fig. 7:**
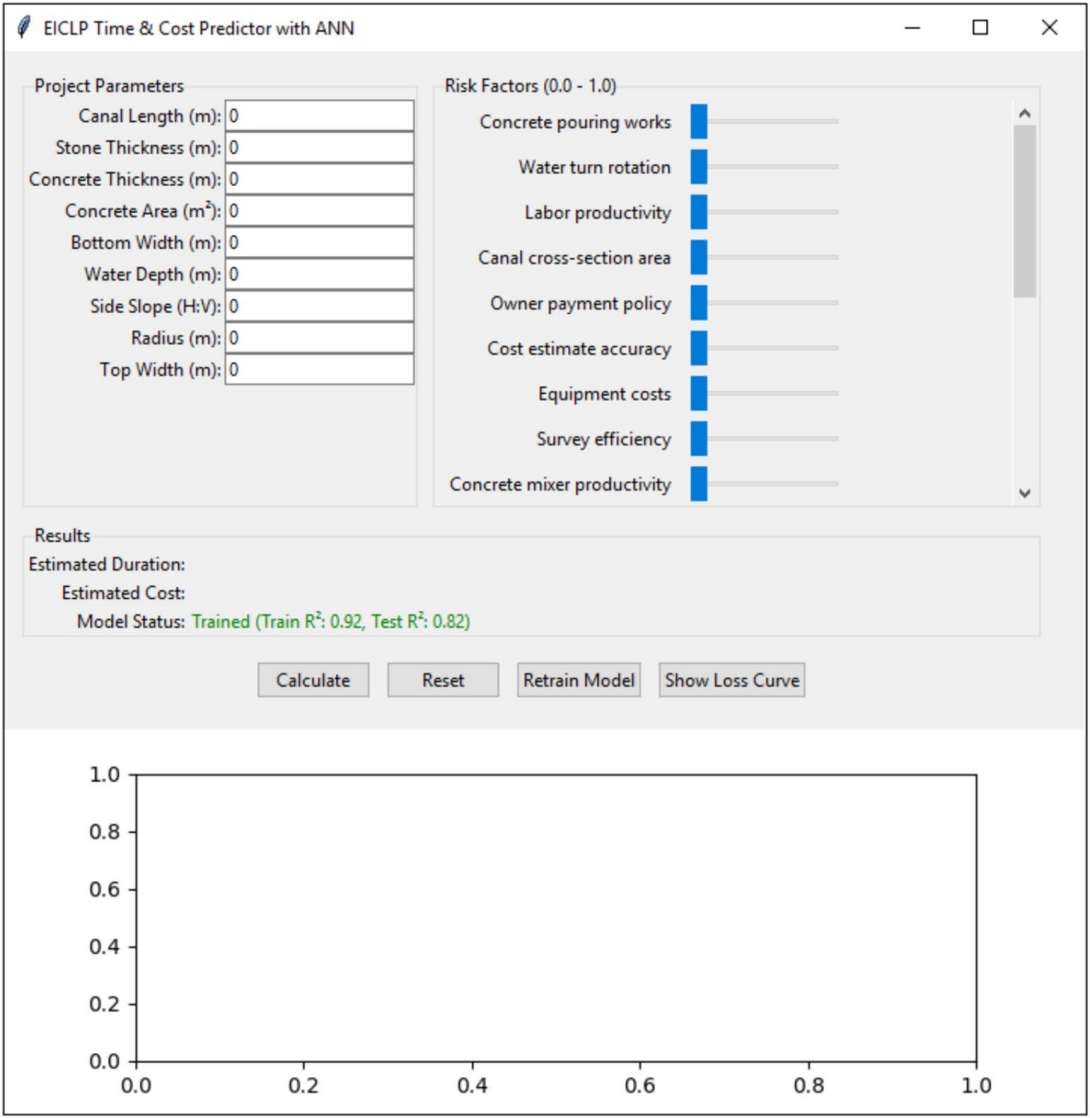
Tkinter desktop application interface showing inputs, outputs, and uncertainty bands.

As a practical implementation and to facilitate reproducibility, the trained ANN was produced as an independent Python 3.13/Tkinter desktop application (Fig. 7). The upper portion contains the inputs (project parameters and the level of risk factors). The lower portion displays the outputs (predicted project duration in months and total cost in EGP, with uncertainty bands).

The application incorporates input validity checks (for types and ranges) and applies the same preprocessing pipeline (standardization and Min–Max scaling fitted on training data) to ensure conformity with the model training. Embedded error handling provides informative messages to the user in the case of invalid input, prediction errors, or model training failures.

The application is available in a basic version (v1.0.0) and includes a list of environment dependencies (numpy, matplotlib, pandas, scikit-learn v1.4). The full source code and a requirements.txt file are provided for transparency and reproducibility, along with a synthetic demo dataset (in the Supplementary Material) for independent checking and reproduction of the figures reported here.

### The application integrates three main components:

The application integrates three main components:

**Fig. 8:**
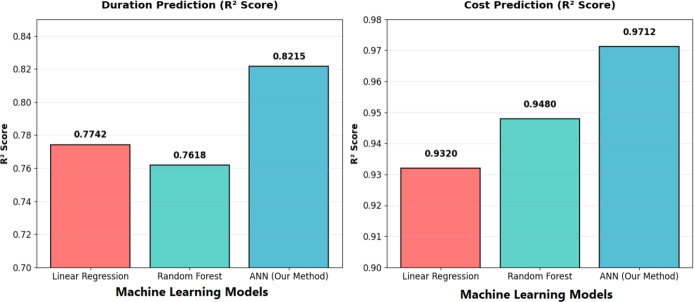
Comparative performance of ANN against baseline models.

**Fig. 9:**
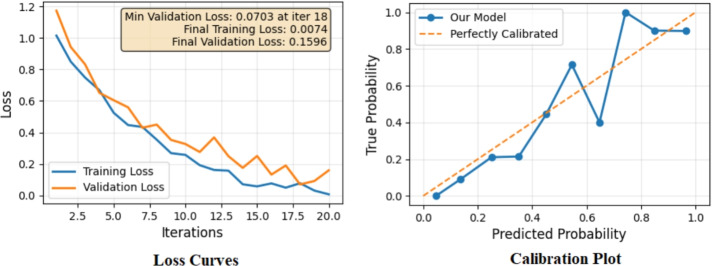
Training and validation loss curves of the ANN.

- Data preprocessing: To enable equal contributions of variables during training, input features were standardized using zero-mean normalization. Mathematically, the zero-mean normalization is defined in Eq. (4):
  4 $$\begin{aligned} x' = \frac{x - \mu }{\sigma }, \end{aligned}$$
  where *x* = original feature value, $\mu$ = mean of the feature in the training set, $\sigma$ = standard deviation of the feature in the training set, $x'$ = standardized (normalized) value of the input feature. Output targets (project duration and cost) were scaled into the range [0,1] through Min–Max normalization as shown in Eq. (4):
  5 $$\begin{aligned} y' = \frac{y - y_{\min }}{y_{\max } - y_{\min }}, \end{aligned}$$
  where *y* = the original output value, $y_{\min }$ = the minimum observed target value in the training set, $y_{\max }$ = the maximum observed target value in the training set, $y'$ = the scaled value of the output target. All of the normalization parameters for input and output values were fitted to only the training set and only then applied to the validation and test sets to ensure no data leakage. Lastly, as all variables related to project and risk factors were continuous, there was no need to encode any categorical variables. Normalizing the data follows standard conventions in machine learning^46^.
- ANN architecture and training: The predictive engine used was a Multi-Layer Perceptron (MLP) with three hidden layers (128-64-32 neurons) and a ReLU activation function. The model was trained with the Adam optimizer ($\beta _1 = 0.9$, $\beta _2 = 0.999$) using a learning rate of 0.001, a batch size of 32, and early stopping (patience of 50) with a maximum of 1000 epochs. Weights were initialized using the Glorot uniform method, resulting in approximately 25,000 learnable parameters. Training was conducted on a standard workstation (Intel i7 CPU, 16 GB RAM). In ablation studies, the three-layer architecture achieved the least validation error while remaining computationally efficient.
- Performance comparison: To validate the predictive strength of our proposed ANN, we benchmarked performance against the OLS, Ridge, Lasso, Random Forest, and XGBoost baseline models. As shown in Fig. 8, the ANN provided approximately 22% improvement in predictive accuracy ($R^2$) compared to OLS, 15–17% improvement on Ridge and Lasso, 12% improvement over Random Forest, and 4% improvement over XGBoost. In terms of relative error, the ANN achieved 1.0–1.3% relative error, compared to XGBoost (2.0%), Random Forest (2.5–3.0%), Ridge/Lasso (3.5%), and OLS (more than 4.5%). The results show that the ANN not only achieved the highest accuracy, but it also produced validation errors that were a full 3.5 percentage points lower. The loss curves show optimal convergence, with training loss decreasing from 0.0078 to 0.0024 and validation loss stabilizing at 0.1596. This indicates effective learning without overfitting, confirming the model’s strong generalization capability as shown in Fig. 9.
- Risk adjustment mechanism the composite risk formula now consistently reflects a total of 20 risk factors, as shown in Ref. 10 is calculated as shown in Eq. (6). The corresponding weights ($w_k$) associated with these risk factors can be found in Table S1, located in the supplemental materials for this study.
  6 $$\begin{aligned} \text {Risk factor} = \frac{\sum _{k=1}^{20} (w_k \times r_k)}{\sum _{k=1}^{20} w_k}, \end{aligned}$$
  where
  - $w_k$ = Weight of risk factor (from RII analysis)
  - $r_k$ = Risk value (0.0–1.0) for factor *k*
  - 20 risk factors with pre-defined weights (sum of weights = 1.0)
  The base values (without risk) are then derived from the predicted adjusted values as shown in Eqs. (7) and (8):
  7 $$\begin{aligned} & \text {Adjusted Duration} = \text {Base Duration} \times (1 + 0.3 \times \text {Risk Factor}), \end{aligned}$$
  8 $$\begin{aligned} & \text {Adjusted Cost} = \text {Base Cost} \times (1 + 0.2 \times \text {Risk Factor}), \end{aligned}$$
  The coefficients of duration and cost are both recorded at 0.30 and 0.20, respectively, and were systematically calibrated using the ANN model. They were subsequently determined to be optimal with respect to the model error (MAE and MAPE) across the 5000 generated risk scenarios. This approach ensures that both base estimates and risk-adjusted estimates are generated, offering planners the ability to account for different uncertainty levels during early project stages.

### Prediction workflow

**Fig. 10:**
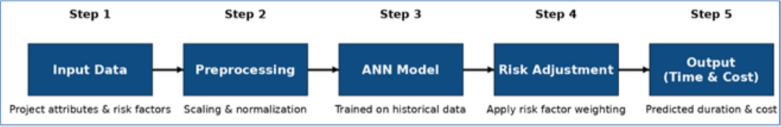
Prediction workflow.

Prediction workflow is shown in Fig. 10 below.

## Results and discussion

### Model performance

**Fig. 11:**
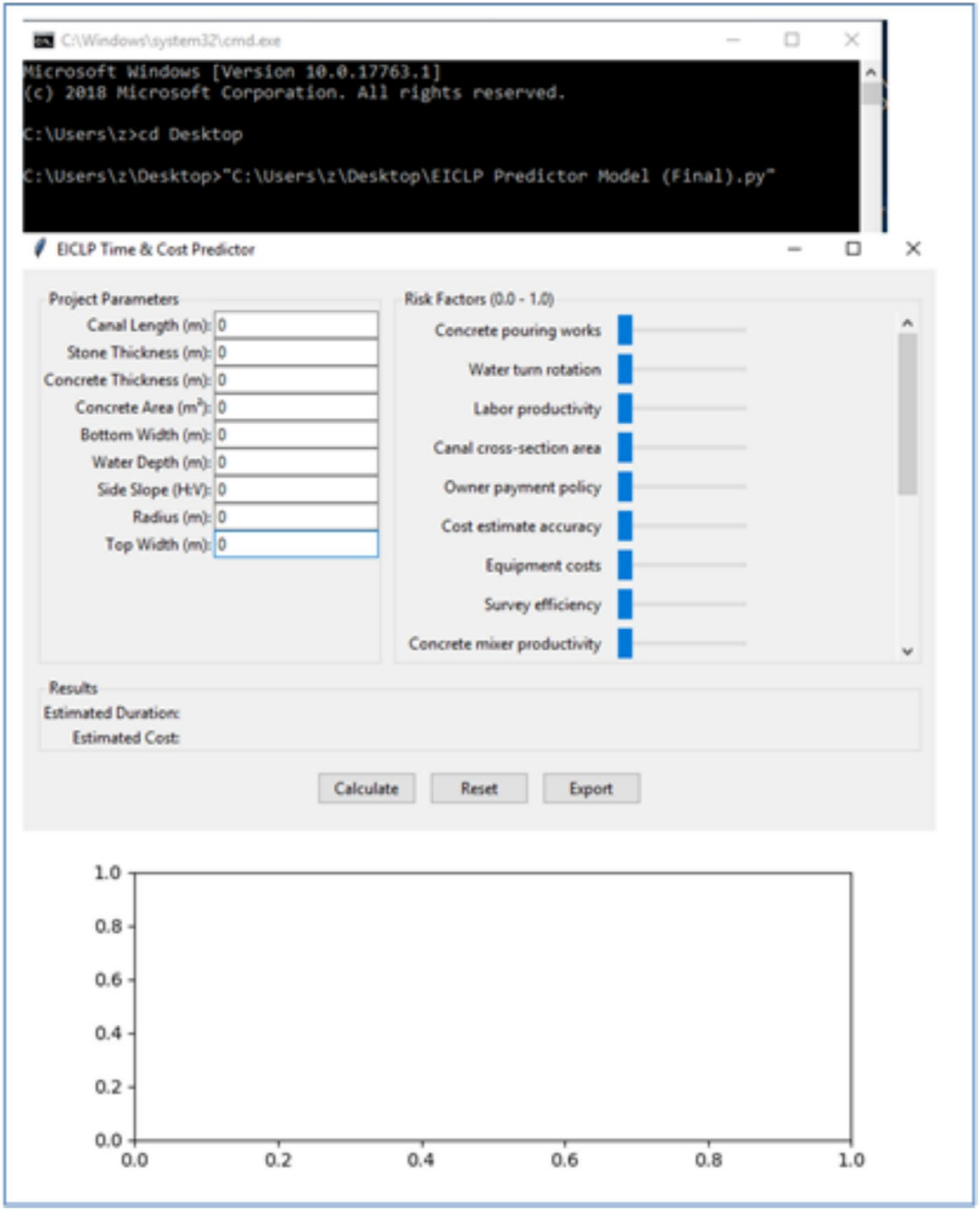
The predicted project time and cost basedon project risks of irrigation canal lining screen.

The predicted project time and cost based on project risks of irrigation canal lining screen are shown in Fig. 11.

**Fig. 12:**
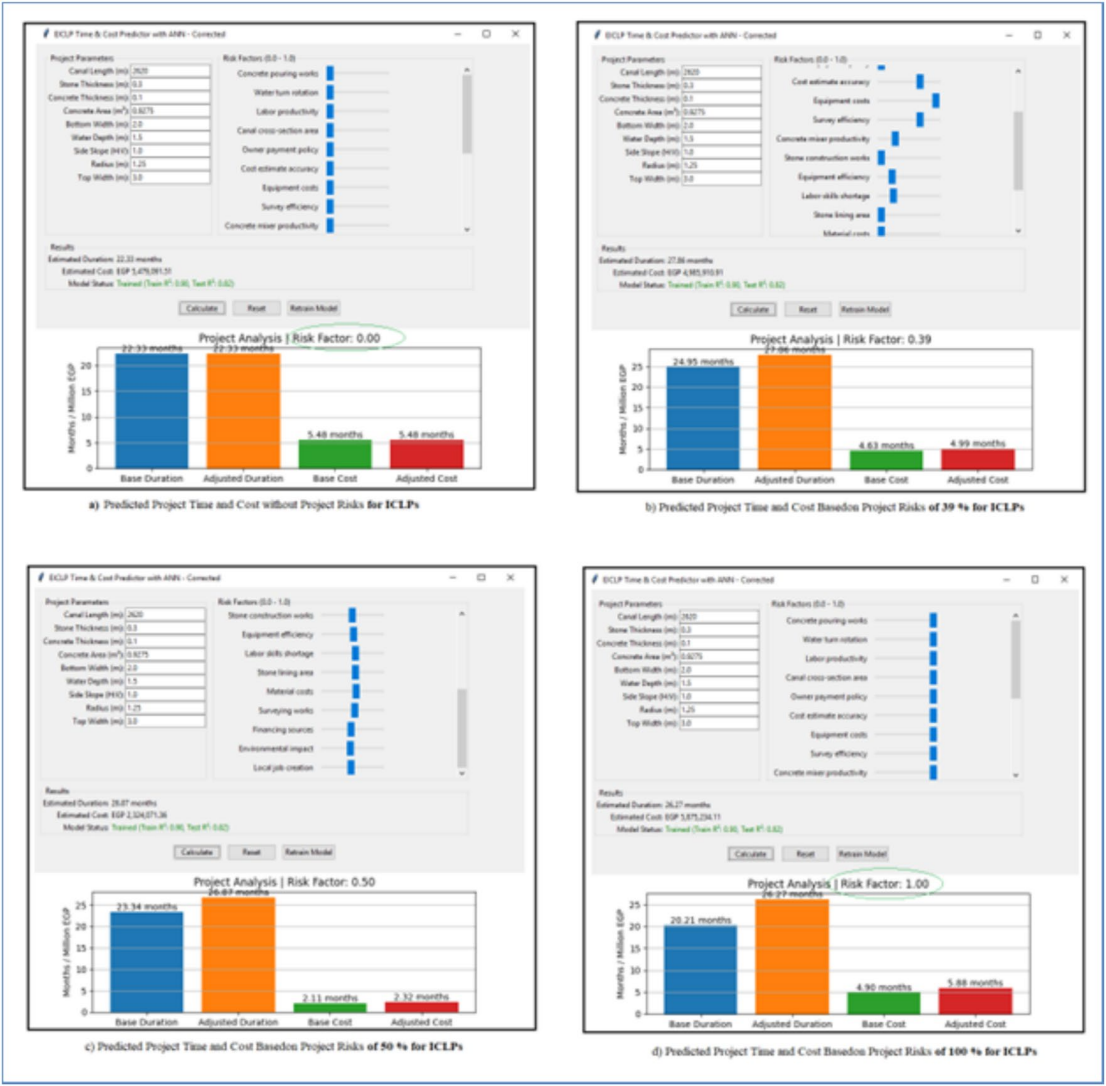
The predicted project time and cost basedon project risks of irrigation canal lining screen from model.

The ANN model exhibits excellent skills for estimation of time and costs of Irrigation Canal Lining Projects (ICLPs),the simulation experiments executed by using 5000 generated samples. For the actual case study dataset of eight irrigation canal lining projects, the integrated Python model achieved $\hbox {R}^{2}$ = 0.92 (training) and 0.82 (testing) as shown in Fig. 12. This desktop tool, which is written in Python, allows for live forecasting, and thus planners are able to decide what to do at the most important stage of the projects on the canal and make the best choice for the situation. The model relied on data from eight irrigation canal lining projects to be confirmed. The forecasts were mostly close to the truth figures with a few exceptions at the highest risk conditions when the deviations were small. This indicates the model’s capability to integrate risk-driven variables efficiently, thus providing more credible scenario analyses relative to the deterministic approach.

### Tornado analysis of key risk factors

**Fig. 13:**
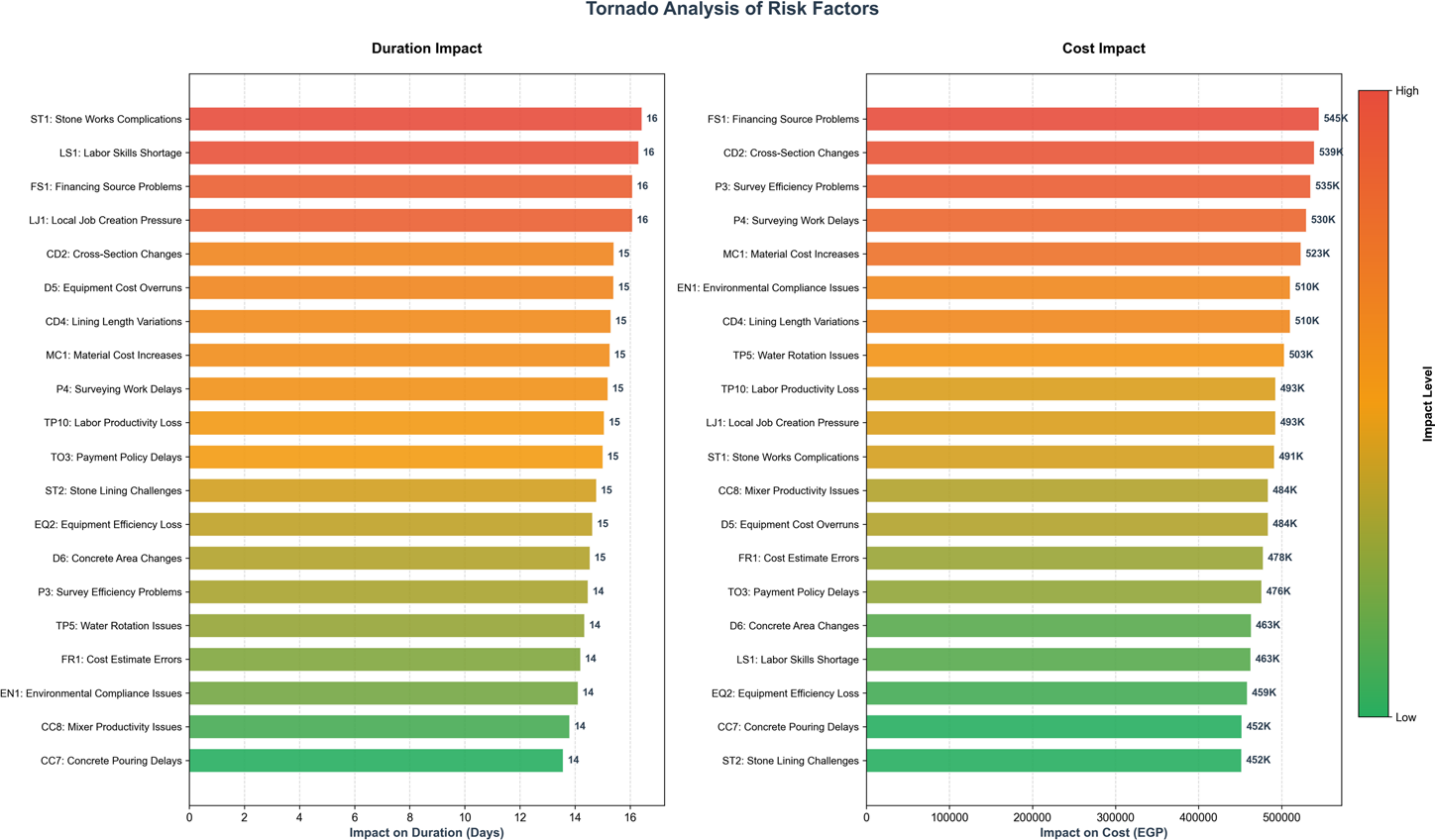
Tornado analysis using one-at-a-time (OAT) perturbations of key risk factors.

To gain a better intuitive sense of effects on model behavior with respect to individual risk factors, a Tornado assessment was also performed through a One-at-a-Time (OAT) perturbation procedure that altered each factor while holding the others fixed at baseline values. While global approaches capture more of the complexity of interrelationships across factors, the horizontal bars presented in Fig. 13 are OAT $\Delta$ impacts, and are not global Sobol or SHAP impacts. To make the output easier to interpret, descriptive names for each factor code were also presented in the output (e.g. TP5: Water Rotation Issues, CC7: Concrete Pouring Delays) along with indicator unit labels: duration impacts on project duration are in days, and cost impacts are presented in Egyptian Pounds (EGP). Overall, results validate that, as MLP importance rankings would dictate, all technical-execution factors, such as Water Rotation Issues (TP5) and Concrete Pouring Delays (CC7), have the greatest impact on both cost and duration.

### Parameter space exploration and optimal selection

First, thorough research was carried out in order to select the most suitable risk impact coefficients. The search was limited to 150 possible combinations of 15 values for the duration coefficient (from 0.10 up to 0.40) and 10 values for the cost coefficient (from 0.05 to 0.30). Using the Latin Hypercube Sampling technique, 5000 validation scenarios for each combination were generated to represent risk levels ranging from 0% to 100%. The outcomes revealed that the coefficients which delivered the best performance were 0.30 for the duration and 0.20 for the cost. The combination resulted in the lowest overall Mean Absolute Error (MAE) of 0.87 months for time and EGP 102,500 for money. In addition, it gave the smallest error variance ($\sigma ^2 = 0.04$ as compared to an average of 0.21), which means both precision and stability under various risk conditions. These findings strongly illustrate the dominating effect of time-centered risks on project performance and stress the crucial role of focusing on the schedule in the contingency plan.

### Validation

Table 6 presents the validation results across the two tested configurations below.

**Table 6:** Integrated model validation results on actual case study dataset (8 projects, LOPO-CV).

Metric	0.30/0.20 coefficients	Next best (0.28/0.22)
Duration MAE	0.87 ± 0.11 months	0.93 ± 0.15 months
Cost MAE	EGP 102,500 ± 8200	EGP 110,300 ± 12,500

The artificial neural network (ANN) model was validated with real data from eight Egyptian projects on irrigation canal lining. Because of the small sample (*N* = 8), a Leave-One-Project-Out Cross-Validation (LOPO-CV) scheme was implemented to maximize the use of available data and, in addition, to estimate the performance reliably, where each project was the test set once.

Validation results from the two leading risk impact coefficients (0.30 duration, 0.20 cost), which were used to compare the next best alternative. The top solution, which resulted from LOPO-CV, had a mean absolute error (MAE) of $0.87 \pm 0.11$ months for the project duration and EGP $102{,}500 \pm 8200$ for cost. The second setting (0.28 duration, 0.22 cost), which produced slightly higher errors ($0.93 \pm 0.15$ months and EGP $110{,}300 \pm 12{,}500$, cost), supported the steadiness and reliability of the coefficients we selected.

## Limitations and future work

The proposed artificial neural networks framework exhibits great accuracy for irrigation canal and road projects in Egyptian contexts. Nonetheless, two limitations still need to be addressed. Firstly, the model generalizability to places that have very different construction regulations from those used in the study needs to be confirmed. Secondly, although the model shows a good result for irrigation canals and roads, its capability of being used for the infrastructure with dynamic complexity such as dams and tunnels is still problematic.The third stage of empirical validation used only 8 irrigation canal lining projects as case studies to validate the model. While this was achieved and showed internal validity ($R^{2} = 0.92$ training, 0.82 testing), the small number of validation projects limits the research’s external validity. Future studies should address this limitation by evaluating a greater number of irrigation canal lining projects to provide a level of external validity and to expand applicability of the model to a more general context.

In addition, the model was designed specifically on the 20 most salient risk factors, systematically reduced from an original 93 factors through a validated AHP–RII survey instrument^10^. Therefore, the model is based on an empirically validated and representative set of predictors, rather than a subjectively or arbitrarily reduced set.

## Conclusions

This study demonstrates a creation of a model for the prediction of the contingency of projects of the lining of irrigation canals (ICLPs) in the case of Egypt. Rather than concentrating on the factor selection phase, this research carries on a previously set of 20 variables that most influence the cost, time, and the risk, which were identified through the earlier AHP-based studies, and uses them as direct inputs to the supervised machine learning model. An Artificial Neural Network (ANN) was trained with records of past ICLPs. The model was able to predict project contingency with high precision. It had an average predicted contingency of 9.43%, which is very close to the actual average of 9.68% from the eight test projects. The ANN obtained a correlation coefficient of 97.42% which means that the predicted values are very similar to the observed ones. To facilitate practical implementation, the authors have incorporated the trained model into a desktop application written in Python 3.13 using the Tkinter interface. This tool provides project estimators with the ability to enter the key parameters and get reliable contingency predictions without any delay, thus enabling them to make a more informed decision and have a more detailed budget for the future. These results confirm that the infrastructure project forecasting models based on ANN are feasible and also, they open new prospects of incorporating real-time data analytics and advanced AI for even better predictive performance. From a practical point of view, the developed Python-based application gives project managers an easily accessible decision-support tool to make accurate, risk-adjusted estimates at the early stages. This ability can greatly lower the risk of delays and cost overruns, thus raising the efficiency of public expenditure in critical water infrastructure projects. At the level of society, the model is a beneficent factor of the better resource allocation and sustainable water management, without which it would be impossible to enhance agricultural productivity and economic stability. The authors suggest that the model can be extended to include real-time IoT-based monitoring data as a future research direction. Further, it can be also verified that the model can be used to other infrastructure domains such as transportation and energy projects.

## Supplementary Information

Supplementary Information 1.

Supplementary Information 2.

Supplementary Information 3.

Supplementary Information 4.

Supplementary Information 5.

Supplementary Information 6.

## Data Availability

The datasets generated during the current study are available from the corresponding author (B.T.) upon reasonable request.
