# Supplementary material for: Risk-indexed artificial neural network for predicting duration and cost of irrigation canal-lining projects using survey-based calibration and python validation
Source: Sci Rep. 2025 Nov 17;15:40316. doi: 10.1038/s41598-025-24125-1 (PMC12623735; doi:10.1038/s41598-025-24125-1)
Supplement: Supplementary file 1 — Supplementary Information 1. [file 41598_2025_24125_MOESM1_ESM.pdf]

canal_length,stone_thickness,concrete_thickness,concrete_area,bottom_width,water_dep
2498.16047538945,0.3926094938462863,0.060312386883593264,0.6689350630721645,
4802.857225639665,0.22524198949851465,0.14025529066795667,0.778590339031958
3927.9757672456203,0.24848861422838414,0.10052523724478571,0.67701048427674
3394.6339367881465,0.4695662565581238,0.13264574661077416,0.588702533757055
1624.0745617697462,0.38192871789787697,0.08200496010306117,0.62063587110060
1623.9780813448106,0.2027591154849889,0.13955232284962005,0.960778768032725
1232.3344486727979,0.23044146285980965,0.0889201678734163,0.706333718405792
4464.704583099741,0.39905053073241675,0.05108376514802984,0.864269861048075
3404.4600469728352,0.20151847515386562,0.14053819764192638,1.00341727085485
3832.290311184182,0.2482424154252496,0.059128667678613356,1.190394828629365
1082.33797718321,0.36462013680997585,0.08193136375904149,0.5393121398410989,
4879.639408647978,0.407568559307808,0.14500619670508047,1.2994103989090426,2
4329.770563201687,0.39558837785078016,0.1450607146937556,1.1279003894909079,
1849.3564427131046,0.26728079283816797,0.10734378881232862,0.58175903194887
1727.2998688284024,0.41365376640426077,0.11318372121697992,1.37357862410677
1733.6180394137352,0.27117472624904004,0.09484455219783197,1.42087240053181
2216.968971838151,0.29761990944778033,0.07932107716980645,0.561077959854863
3099.0257265289515,0.4239474215354072,0.0828664545369916,0.7768776481472037,
2727.780074568463,0.3948898697141644,0.11725184560770384,1.3062012797930613,
2164.9165607921677,0.4547670231482534,0.12523745294376798,1.248259690383658
3447.411578889518,0.39728386769010304,0.12915790437258484,0.684521019356377
1557.9754426081672,0.37049258100064153,0.12896181427945538,0.70934932333671
2168.5785941408726,0.22810243034842775,0.05912061030486904,0.870472102791383
2465.4473731747667,0.31031474091783007,0.09944203047025815,0.98452298519102
2824.2799368681435,0.2795607103045176,0.05575587600166443,1.118254771530296,
4140.703845572054,0.2731968930137251,0.10495288823237356,0.8689136395697724,
1798.6951286334388,0.4919031664257337,0.0941530501373377,0.9625347161331479,
3056.9377536544466,0.3179293174000281,0.13877041827582998,1.247470938133756
3369.6582754481697,0.467613966553134,0.08509150125520787,0.5366832028905979,
1185.8016508799908,0.3893415877991789,0.06170670164276059,0.752436944344020
3430.1794076057536,0.4384433910624945,0.06429916820528359,1.213349585884552
1682.0964947491661,0.35079112793155764,0.12615106317174724,1.39520683768719
1260.206371941118,0.37307116538790774,0.11182180633162611,1.011677442115666,1
4795.542149013333,0.34775530814565914,0.06011226761227903,1.032113485265315
4862.528132298237,0.2585728963394134,0.058410680611499746,0.607172011339776,
4233.589392465845,0.41673563457845164,0.12009691314591199,0.947412366823454
2218.4550766934826,0.2842317087322567,0.05727630063641936,1.032617266455023,
1390.6884560255355,0.20729478992943617,0.13218600592903562,0.74247050363472
3736.9321060486277,0.39364168877215033,0.12062422271564961,0.76924323094938
2760.6099749584055,0.2531332038221147,0.05813487806418998,0.877284163104622
1488.1529393791152,0.4821375753058743,0.058483771408519195,0.52007119777772
2980.707640445081,0.4861785731007762,0.14866395785011755,0.8220791655831783,
1137.5540844608736,0.47445931706613453,0.08742707957561204,0.71144800699654
4637.281608315128,0.31104761007663334,0.0870642147066891,0.8274973521779146,
2035.1199264000677,0.20463698495866023,0.13127995672575027,0.61976213181925
3650.089137415928,0.4784955687763176,0.14472485773838586,1.390527280739895,2
2246.8443043576435,0.3284552444951943,0.1486001063822871,1.0935924535540487,
3080.272084711243,0.48999644571310086,0.12533781852589415,1.179102319144489
3186.841117373119,0.48908599312677586,0.08762595855309158,1.289171238607338
1739.4178221021082,0.45590283664020803,0.05835007166986688,0.99844219892905
4878.338511058235,0.28833466762087573,0.12771469159274368,0.58692028808742
4100.531293444458,0.3155293185805776,0.10584042497358051,1.037106541818548,2

4757.995766256756,0.45534100145505707,0.09242220092469763,1.086841118020879,
4579.309401710596,0.2950766015468833,0.14063543850947358,1.24543947418433,2.
3391.5999152443405,0.2508478240058278,0.06111974823061514,0.931659546229679
4687.496940092467,0.36704037873750506,0.0992625104290859,0.6275803027955638
1353.970008207678,0.4808464322482343,0.05113536447674191,0.7837759057987245,
1783.9314496765808,0.40880893900249193,0.09686606419941263,0.86308229639863
1180.9091556421522,0.37101835102680947,0.055630327568183736,1.14591724133160
2301.3213230530573,0.22915294813123058,0.061881791626807195,1.0707783046689
2554.709158757928,0.3845021680097509,0.06175262467771049,0.8560967258978462
2085.3961270955833,0.497016155031279,0.11492103021160635,1.4865152487929798,
4314.950036607717,0.24202520457095722,0.12460448792654233,1.105774819356887
2427.0133067743573,0.355498895709121,0.10833687650971596,0.7372267917359945
2123.738038749523,0.46321192157838664,0.14621725484745418,0.601782472620403
3170.784332632994,0.42223058532626134,0.08748705795237041,0.652859139184332
1563.6968998990505,0.4091047222985804,0.07857120862818608,0.745957728384508
4208.787923016159,0.41074522519613277,0.13685991281894602,0.660681373259555
1298.2025747190833,0.30784734536592656,0.07235958385194527,0.68656702405130
4947.54774640207,0.28807755327934803,0.14632225394406112,0.7850951686938471,
4088.9790771866296,0.44280834664355406,0.051215447468981636,0.6733735952947
1794.8627261366896,0.4430340184037542,0.1469878826707639,1.3967654246264252
1022.0884684944097,0.4601216955740311,0.05431599119505762,0.580233745661642
4261.845713819337,0.4739721657669414,0.13911431136980712,1.0245113895702547,
3827.4293753904685,0.35340271965828135,0.10277011090862997,0.9103968269896
3916.028672163949,0.3504548884061599,0.14929647961193004,1.4823786169086064,
4085.081386743783,0.43948855369003254,0.05737965647353989,0.612038902168052
1296.1786069363616,0.39498917923332955,0.10538542844013207,0.89785559904574
2433.8629141770907,0.410590063177311,0.1469302535619099,1.4694704332753687,1
1463.476238100519,0.4387378008308303,0.10230978441701488,1.3655071258939802
4452.413703502374,0.4670016025452699,0.11293986381352625,1.3170720709492798,
3493.1925073102316,0.30139854705546076,0.11957486889846171,0.757902827044939
2323.592099410597,0.3126748857919832,0.09545410647677732,0.6708875873900658
1254.2334011440946,0.22819458195226072,0.11275580800840634,1.16864321992443
2243.929286862649,0.37348404229885224,0.10843143119231002,1.429375989127586,
2300.733288106988,0.21078268213902263,0.14011580104909888,1.05676289301393,1
3918.4247133522563,0.33967940543973807,0.05454463803414579,1.07161268946989
3550.2298854208525,0.362793390412273,0.07809631895922303,0.7799790936602842
4548.850970305306,0.2859623756384853,0.14504114840765586,1.269492933191937,1
2888.859700647797,0.37724997817070327,0.13902637838909163,0.687043748557523
1478.3769837532068,0.20915007498171484,0.09556567527857 13,0.823679236404243
3852.97914889198,0.21120445662476434,0.11201325978015367,0.9254364386164168,
4043.14019446759,0.44678016819789745,0.07773811829811327,1.007610378684455,2
3245.108790277985,0.30805719242337887,0.06881211597237613,0.742409732415080
4083.868719818244,0.23811815379556545,0.09636984049399822,0.614836824739203
2975.182385457563,0.35667297801 64413,0.08533522280260528,1.1106200424416326,
3090.9313175279763,0.43099806592958323,0.10836561118508 72,0.788630553240255
2710.1640734341986,0.264746308249053,0.05777346369649849,1.0812382214226122
1101.6765069763808,0.3868671427457001,0.14743948076661664,0.654362715274202
1431.5657079732177,0.2256042394981304,0.1486210744479603,0.9811401018548175,
1125.716742746937,0.21550451635058232,0.11981617140197451,1.032589432551585
3545.6416450551214,0.3594063894704444,0.10360963663441203,0.551823536822426
2257.4239243053066,0.36219053648303196,0.08095276162863277,0.836604278193920
3034.282764658811,0.391228970449462,0.13137950197069487,0.6344146769389742,2
4630.265895704372,0.4178274001167985,0.11847311725538792,0.5633749704727677,

1997.1689165955,0.4927556238387603 7,0.0662616939344891 4,1.4899602323899452,2
2641.531692142519,0.3548901044903586,0.1410927184493842 6,0.822353844974723,2
4022.2045541721945,0.2968869418823738,0.1322537242923169,1.3098744458546348,
1915.1926619664898,0.43855585843061107,0.1449799913291924,0.754640654763763
1307.919639315172,0.2812496753786223,0.122571950838836,1.1815027222239292,2.
2159.005811655072,0.3316914262116908 4,0.11134151959357899,1.260227859889686
1644.8851490160177,0.2235369144026798,0.0918243036290618 8,1.095638740607844
4718.790609370292,0.20760522302463727,0.1432728483354013 3,0.971576188550158
4232.481518257668,0.4887945244033775,0.13660638895004085,0.911840914147268 5,
3533.615026041694,0.45079403615366176,0.05452186701061894 5,0.848868266542999
4485.84236075087,0.4087922618281094,0.05263669744972520 5,1.4295291442478257,
4214.688307596458,0.32268588332428094,0.08764633668780496,1.330619407787729
1746.2802355441434,0.2519882960212537,0.1310553330781833,1.4650269106665126,
4570.235993959911,0.24693111280132582,0.14872761293149445,0.624297223485544 7
3157.3689676626027,0.2750728694493786,0.0650416891103528 2,1.230867475203644
4229.7606206562505,0.36476799941183613,0.10941307153521351,1.43834045682103
4584.365199693973,0.4143787768100186 7,0.0880890856631021 5,0.68123306616566 0
2272.0138998874554,0.3980592130153194,0.1469914397814603 1,0.566496267366777
1440.207698110707,0.2839801690837829,0.13421189231357086,1.241120649290059,2
1911.7406501677667,0.48645958419895824,0.1338328704711137 8,1.07447311317991 2
2708.431154505025,0.42136907500873055,0.09686931597949702,1.341828776758272,
4272.059063689972,0.3663062157534202,0.0914819502337665 1,0.639772376626289 5,
4442.9223330253735,0.3835162238703057,0.0773407071930706 2,1.29526731185989 0
1027.8085221247627,0.32588001872833694,0.0556375496650927 1,0.701627320047 74
3042.989210310263,0.2743192968503472,0.1364722376255053,0.663655942865704 5,1
2669.644012595116,0.3067918035953785,0.13129010091300775,0.664265797930992 9,
1888.4312418829209,0.42735383313931075,0.14997176732861306,1.31457472023138
1479.4614693347312,0.20431804658892677,0.14966368370739053,1.1651972206962,2
2350.460685614512,0.23482179215207488,0.10554317056026274,1.02306542476911 9
4771.638815650076,0.21380079260652585,0.12689874151805103,0.858830484123502
2292.811728083021,0.21221864069569105,0.1444765729882428,1.3772005408131083,
3075.162486973464,0.4566381752033022,0.13496473906774115,0.8924451074226354,
3812.0758355807116,0.41109735781400714,0.07473481017431977,1.31659943947157
2454.518409517176,0.3422521487261976,0.09505441353100935,0.9391349085702184,
4887.128330883843,0.22935024819530045,0.0629159415151495,0.8769444294249076,
4849.789179768445,0.3474847625350497,0.14540510272587223,0.9626797856696064,
2007.1291833014566,0.3420415312341697,0.110617463445088,0.8013778741641421,2
2988.9940235695417,0.25196056097300457,0.07286428055034627,1.24760938017625
2203.5132392670785,0.33015549477139194,0.11717006844058567,1.00272039009247
2139.3619775098705,0.319551420319212,0.11181282404578957,0.7322126951468173,
1147.5477894181313,0.384755029415665,0.08581627180328405,1.3995745732745686,
3438.2573359195876,0.39052809526029314,0.06135575921996290 5,0.8838912213732
3010.716092915446,0.21359120293161338,0.11715731955927995,1.04355286111398 8
1205.9150049999573,0.31238378438794134,0.10203077009037934,1.40647211096454
2114.585856946446,0.3877579747142709,0.12723183917356393,1.124237995913992,2
4633.063543866615,0.3509408775740263,0.10201635011119933,0.6168980407083641,
1958.2475626678897,0.4569469523564967,0.135218150031854,1.439832123613475,2.
1579.5794883648923,0.3976080894856835,0.10519068387744855,1.12770805307141 7
2957.811041110252,0.24888032812442892,0.10609379715353862,0.834905614657086 1
4942.601816442402,0.22117062422012895,0.13766536026583448,0.63927207266338 7
1968.2210860460018,0.3927257834618947,0.0903482866212397,1.2940251892702959,
3688.5421896235143,0.20795339316248657,0.06340152284506408,1.12007275592851
4046.4784613148704,0.375732674382039,0.0528782676313339,1.0334610919763216,1

1950.5501759695985,0.48206907242748726,0.1255137255673619,1.393892583050957
3912.865394447438,0.3726422533627637,0.11203095513534647,1.2885972112245307,
2471.132530877013,0.3164509778619566,0.12040797680992235,0.6516748797327512,
3529.223322374318,0.392986465532706,0.07129641615089108,0.8117220677955482,2
3534.1188430435786,0.33747586714745503,0.06363714755867697,0.74848913981446
3143.098736299034,0.3636850367947805,0.0514544665667882,1.243946292572677,2.
1361.1590802176333,0.48243944263295757,0.0850587558806597,0.533532434735779
4341.2099823569515,0.3158307913402323,0.10899176868546331,1.069889684871316
2283.120259886943,0.48835716914717425,0.08922440450997324,1.262458685740690
1746.074041599417,0.4716051925868191,0.09374749220237291,1.3767656367617493,
1163.1005662190557,0.2587373404367889,0.14041586944937484,0.842081748715907
3363.5717727529673,0.22080839026254964,0.08482554670233003,1.32125730467201
3710.2574473691297,0.230233400413228,0.10139894891598109,0.6106317369552072,
1066.3513157114246,0.20546654769546494,0.1283653012741143,1.346452291734518,
3048.372233197124,0.22833288822677852,0.08965427823212702,0.627488662331982
1905.983100791752,0.40490203202490704,0.11220867002278734,0.897287290560367
3580.6911616377993,0.22135659453806872,0.1362363708746745,1.297295365779553
1697.4657160199658,0.2956926890881284,0.1449520623657642,0.6499174273487738,
3763.7509524098637,0.4534625932908364,0.0647073480929038,0.7292513952326415,
2546.94138520215,0.20698158072074777,0.14265876251614945,1.2222525683930663,
4746.919954946938,0.4443405447766807,0.09921162930795382,1.2200365365460744,
1550.0837765839728,0.28455643243202,0.07582443882989584,1.1411476328852972,2
2364.265404201034,0.2354494482864969,0.09591357562382613,1.1939484444671,2.0
1453.8940849623564,0.4090211496092452,0.1480032575285477,1.0427244433475962,
4698.774473114251,0.3886828540339652,0.09926180939928696,0.7517990589069528,
4509.357413523924,0.4632416040581159,0.08287516102875082,0.8456959935039194,
2031.7665108606225,0.420521313141165 7,0.11334008543167258,0.681597716801425
3639.9361841367163,0.4410442791154546,0.0740145618778193 1,1.40845056133628
4268.888800804863,0.2846103717713919,0.05758633281086639,1.0833917947661205,
3220.8032463978493,0.2532318631339169,0.06288797219106493,0.900851416763639
3118.602313424026,0.4251844254922575,0.06280458389577724,0.9620058036441327,
1967.4091636018068,0.44205042178017917,0.0651902693512294 3,1.44728333961181
1372.4110712235968,0.497151542600202,0.0638827172649410 2,0.6533514031160802,
4588.863031813307,0.32378530307342795,0.11408747448032146,1.086229832016797
4601.672228653322,0.3116054257378349 5,0.06818800843991449,1.005888678884466,
3532.4058290930716,0.4329238882225991,0.08456672833238632,1.111454235434648,
2356.1191641948026,0.30224106207590534,0.13967884099060118,0.51811018382084
2396.8382984506434,0.4792271976810694,0.09739616402628723,1.372123908944151
3903.8227154809574,0.45752382555290355,0.11675577385210271,1.43211828248361
4588.441039810308,0.3286982082125055,0.06723198712016298,1.065133183589209,1
4548.345697060469,0.42526132033744923,0.06922890188086708,1.19665082387689
4119.502183430495,0.4263628622254047,0.05408686162664788 6,1.422499381177295

th,side_slope,radius,top_width,risk_factor_1,risk_factor_2,risk_factor_3,risk_factor_4,risk_
,2.5608579514700978,1.1851329288386196,1.2582631959290227,0.7505628618514248
6,1.728808564371392,1.541900947378358,0.5245869164588015,0.7514288244324765,2
68,2.3644325402502195,1.8729458358764082,0.5221235515289973,0.55500714040031
6,2.4100725695742837,1.7322248864095613,0.8236102191495412,1.60460302259846
08,2.136196006953579,1.8065611478614496,0.9886431904046658,1.49570679143270
3,2.604666353437084,1.6587833667107175,1.2704074178077933,1.2119463136247208,
5,2.9015505221535225,1.6922765645178526,1.1832953766065053,1.76625567345379
5,2.8883527693601643,1.8491956515653194,0.9459027063767483,1.70850522942507
7,2.176259057106198,1.249668008859186,0.7736266662816659,1.3780315465951283,2
3,1.669857068761133,1.4894249636431405,1.4971245001577111,1.8024069207698878,
,2.977261798443502,1.2212094418196022,0.926181302235973,0.8087618150551523,3.
2.758347129668901,1.9876680079966471,0.9513870243296755,0.6678794290965918,3.
,1.6869940218049002,1.9440593396866133,0.6636238211916692,0.9046244172754845,
19,2.8812628239260585,1.039426811368506,1.2948095487499294,0.58563028413397
71,2.8048445430931923,1.7055751725156885,1.1936822257814885,1.29675429200154
32,2.2782570856891082,1.9252483174156658,0.7207696127887604,1.90490853844244
8,2.3869131536173938,1.1805753451273335,0.5823810456158427,0.55901531100276
,2.0985040558051953,1.5679452305526294,1.180499302074713,0.6831648710150402,3
,1.582142458233047,1.9154882975880418,1.1545112142811353,1.1782985424251529,3
2,2.0027958624688513,1.0339459785857987,0.7732595269982093,1.90081252629185
3,2.7042801728970174,1.69742026724684,1.4508635622504102,0.9742341574651258,2
03,1.5069480345069044,1.2973490073725507,0.6510578917809002,1.26085221303258
2,2.000248757536716,1.9243961953765303,0.9323348010426019,0.5623592885755881
13,2.097253040386415,1.9710582451653678,1.4436159201675989,0.72251480144313
,2.3060934044068846,1.944266489113434,0.9197273169261223,1.9799451844382703,2
,2.8797834246191405,1.4742142166574639,1.1385259476640854,1.9476780446540682
,2.0195189915489418,1.8620426509893133,0.8975943979627943,0.5074099714016145
5,2.0204298028443413,1.8445493985350705,0.77421520234867,1.9277176781348584,3
,2.6062518721646226,1.3191004732432556,1.4839776479598283,1.4586799067232505
3,2.178326911334711,1.8289154741506772,0.909334006315043,1.8018774417800332,2
4,1.8369072344099728,1.0370076347154926,1.3940992036791344,1.18210978345078
93,2.17865927419904,1.596269878482053,0.7299546058910862,1.2733940428688753,2
.7112855305697,1.230008837287703,0.7131047040250833,1.2332698703853802,3.329
3,1.764580479759335,1.1205668857772788,0.5311340828845114,1.5002963863191565,
2.2475516591092197,1.0769532016292092,1.151666825375885,0.7094768821344694,3
6,2.1283881742568216,1.6962887758781398,0.8685263437237611,0.544960384809017,
,2.87226885160215,1.3398749637680663,1.3643582498396372,0.9618949123867864,2.
97,2.04359084867495,1.7247667715287616,0.9732099066915572,1.55702114410497,2.
1,2.3708825254170653,1.0653563407989424,1.4681934279147217,0.80278017818442
6,2.4483964318792957,1.3152903378306102,0.6855255157031211,1.510148649987376,
64,1.5196416848825005,1.5394912923753372,1.3686231679556107,1.95486806916090
,2.495306058025066,1.7907231648389637,1.2765968527917417,0.6408510736841275,3
46,1.7670539503046272,1.31875250293207,1.270921844644052,1.5089031773376895,3
,2.941605476204183,1.625891376437009,1.3447832281168877,1.1656253289568568,3.
12,1.7229940916296695,1.8859777482361872,1.261023990942999,1.802213381566258,
2.1219361855905357,1.6158631881823045,1.1262203216314153,0.7657246841710789,3
,1.6280245021179658,1.2329594747536339,0.6312448776823876,1.5389389283392467,
6,2.995311377768921,1.0244007815565381,0.5325261794912519,1.7571729344721654,
4,2.253292515496864,1.8700988739009299,1.4208478478156867,1.9169213292110348,
73,2.3930775259800656,1.0212694108503872,1.116650314520738,1.52487204243446
7,1.6006147160826412,1.8747016726841994,1.2965372909761763,1.245762146012641,
2.6249407055987666,1.528937134027212,0.9815223515125501,1.4267708603018208,2.

1.8148583896433785,1.939067698512896,0.6173081889623945,1.8033574767180773,2
847081434161071,1.7987832357736653,0.6251857922025504,1.3559146199582446,2.7
4,1.8077094607230109,1.9979341105333375,1.1855652872289713,0.545580589545255
,1.7860315809954999,1.3507118154517102,0.9303058948994627,1.8964230432604667,
,1.5548245017721423,1.767188288931127,0.7005247267003367,1.5342901265473416,3
51,2.208100417664999,1.4019309136092422,0.9915945467414369,1.514770078665869
012,2.3472616998939246,1.479875620303909,0.5642089370751341,0.823512728596575
119,1.5985629591425286,1.62750546321837,1.081971401914399,1.488328205348986,2
,2.663291425042516,1.8736771141863389,0.7689934044350977,1.0907966084812377,3
2.1799332521220416,1.984083469199295,1.297559100637188,1.4768494655622382,3.0
,2.28658540399137,1.7682734138645182,0.8103619589235738,0.6598895454619861,3.
,2.161144120407342,1.4177667821673339,0.9552201490818012,1.486767956904554,2.
7,2.101144591312989,1.42135700227707,0.5116205399081006,1.999120588656,3.9843
,2.3394604969623267,1.7375823015888914,0.5724468877945589,0.572318058290834,3
1,1.7328603688960689,1.2387771457683023,0.8924935564066848,1.965761276331976
7,1.7728921957429067,1.1104741131313947,0.9799388347171235,1.110361941084306,
58,2.792678431520276,1.3546221576407764,1.1000205481193037,1.806130175505888
,2.919173193200449,1.2872389916540818,0.7916625787093032,1.6735782260874101,3
548,2.059963974419629,1.29630812045599,1.1949818861265427,1.350524391480001
,1.9061170097153306,1.23360775104991,1.3601223971892047,1.6076738138769897,3.
2,2.4659993148585237,1.0420931896363619,1.2798509888933498,1.817773341952022,
2.1131012566471448,1.0178739347334138,0.5396188253483571,1.1062104824765495,
15,1.5380795334905173,1.9877223897360317,0.9805069472578289,0.990549742344858
,1.7342288960492855,1.4277731337358623,0.6049301784181749,1.501390078345377,2
3,2.573958343271096,1.3843266471596816,0.7420450158625275,1.7117689129118878,
16,2.488385912865227,1.67964728269307,1.4866625932671464,1.6434277021649235,2
.5406439887552252,1.2182538878650642,0.6424955429015132,1.6967204732879517,2
,1.8329582428994242,1.9499611839502253,0.9988881534513561,1.1533749722193996,
,1.846612194882107,1.7863450144155522,1.118155734318119,1.7267513242625325,2.6
98,2.5078391153980926,1.0894110023122594,1.202464970544104,0.680313583055912
,1.5295658066315463,1.4175807757849284,1.059648683486914,1.3167336469412743,3
1,1.6561628729768607,1.8791183075621647,0.5097708474191837,0.508637990747182
,2.699874128059784,1.9447320222914102,0.8264613082439919,0.9868787448504055,2
.7678169930815004,1.4674015112498697,1.0177116433847115,1.0496923023070968,3
98,2.4791191617778123,1.6134113892107078,0.5878664991448334,1.094259037413883
,1.8572741715700898,1.1670339460920753,0.8506269312091808,1.5432008100347931
.6491620891390177,1.9911686261369765,0.5332031087913666,1.0828371519055187,3
4,1.8647582864991812,1.2316717013834433,0.5785784971550207,1.173040433933795
7,2.583400397783489,1.9427317741351255,0.8969232762015961,0.856316196311491 1,
2.7835447021594284,1.6496466489923685,0.6327157540430604,1.0598776874440101,
.7453297968504873,1.6077367948788592,1.067540848261558,0.8409044408947273,2.7
2,2.095775294427682,1.5126885110165085,1.1894649691372656,0.6097938856526655,
5,2.5021277048559694,1.2306698117177586,1.3005866991090829,1.405172890072066
,1.8074764431237313,1.1765280320055083,0.7001502442448101,1.5023191978325243,
3,1.93972159539152,1.2204862090701754,0.6674825822590698,1.4292355190144217,2
,2.84450372778168,1.1864382621442546,0.6045678403344003,1.1952410657008585,2.
3,1.519502885266104,1.7795844735667532,1.1364302495436367,1.0696786702688448,
,1.6282627962817022,1.35012525916673,1.206475726486901,1.795000474357738,3.09
9,1.811829382719041,1.0578426765633964,0.531586144825642,1.278622677704327,3.9
9,1.5397983058107296,1.9691026301408114,1.4362122462436897,1.218772816444596
06,1.7721531526346959,1.8837858849634257,0.5519712836514724,0.538463098709653
2.3745623414545385,1.927752283195213,1.0412963353010705,1.0118717414392346,3.0
2.1321368258887468,1.9949078226464354,1.2090605194509165,1.0702934281787857,

2.839007566615462,1.1738952492194872,1.3709691237460855,1.0982341713453507,2.
2.7261653426076613,1.3962420189026588,1.2140869321324277,1.3702585538409984,3
,2.012726027546814,1.7582384757040912,1.3017280830697917,1.3004038200903176,2
9,1.8891351501469387,1.6960206180537922,0.8394501925428061,1.411857639190167,
06953861225090003,1.153895906339855,1.3148251137465121,1.6473248923131925,3.3
6,2.3854424137722114,1.8158331249906179,0.5801148463846751,1.7194786080741402
3,1.9020954612343144,1.2244405718366607,1.3948166560605277,1.577184614339741
4,2.436223361773701,1.223817614822623,1.0475923761537362,1.933285543932402,3.
,2.1141174782868606,1.5369744228934135,1.3172977699624937,0.5273488740145424,
53,2.3280707712779702,1.592939934841703,0.9523182845183001,0.793666978525319
,2.154189793702975,1.580086207837811,1.1435776951965249,0.5113443124672132,3.9
1,1.9416986393128766,1.0914868373977549,1.0264026609361132,1.471212071231904
,2.9226799604432347,1.8774608626303804,1.2315895217553319,1.8470458234249802
7,2.6454086912396413,1.265600042588706,0.5816299820305896,0.8652234448644489,
4,1.7101697636496789,1.1295149212828042,0.5603520839905638,1.89055181719446,2
78,2.8027019638468693,1.8887480798689773,0.7471032340101464,0.590401085434341
2,2.2311467973742705,1.9556514982297535,0.6595446801131883,1.901654040208271
5,2.841828340341137,1.8621276172654506,1.3717835665922018,1.0274340308594516,
.6997828839209728,1.8095160747248782,0.7192139873580443,0.6521312426738248,2
2,2.1378202567038502,1.6552419806390217,1.4758652558191314,1.228807638779488
,1.533703962480176,1.5508573706091393,0.8368957917711068,0.885164851580762,2.
,1.9030160390774191,1.0869867599114102,0.6821179156886993,0.9273093529428842,
3,2.3124513219913005,1.4084532130706986,1.2896985071424791,0.960934947389429
45,2.450217329739221,1.372688517012314,1.1587077755008761,1.7045388467489953
1.8868315281498034,1.2597537837603263,0.998195716453139,1.3087419150128818,3.
,1.7090341111092362,1.723420113688585,1.0553635509376313,0.9669615487340055,2.
2,2.7523953551989484,1.4958757350787708,1.219201778272264,1.4155006749663777
2.9766032710553283,1.081046215907648,0.7284547413312987,1.5742260113347903,3.
3,2.2885352734540287,1.2201832019498113,1.496333916056742,0.9089360011003007,
5,1.7575189287724553,1.683258763659596,1.4747931621467831,1.1203236522880273
,1.9084609897790534,1.0761308594903003,1.1503256863469369,0.6828291400054214,
,1.5275860148212002,1.8512069140487686,0.6995424509291451,0.7717240243020205,
7,2.8714482098407483,1.4951465270139743,1.1802282424312913,1.521676780947474
,1.6766266243352117,1.480586577326648,0.5721984089791758,0.7721575215434404,2
,2.3647747132713803,1.5924077846595175,0.5306525022058061,1.2877450755005904
,1.911082831030809,1.824680965925149,0.7576828885112137,1.563569392583649,3.6
2.3312670037736902,1.347809207902193,0.9626229567393163,0.6603153846421157,3.
11,2.4771305825277965,1.6780161525590636,1.3682725054083806,1.350968329443355
93,2.7446127055608027,1.5657319639957912,1.227169069766308,0.884844175552045
1.8096319076409049,1.2670282701694213,1.242706521199981,1.9443903128563698,2
,1.5164937429877208,1.878629986355158,0.9254933344480754,1.2253184700798871,2
114,1.705328445103211,1.797426021606928,0.8459349925469629,1.708988824662737
6,2.850027962772158,1.6584518346584254,0.8710387629846027,1.3253398132343928,
68,2.810835116343773,1.850581729094242,1.4876495637360578,0.5651187992822287,
2.3961196532554627,1.8672942009598024,0.5401091914124825,1.4497270633910335,2
2.40077529065048,1.7083629767150348,1.3670314961224486,1.9271050133099856,3.
497555011819383,1.837013328363672,1.0786754085723933,1.4024177302194967,2.43
9,1.7630569179351743,1.6974714616692836,0.9386154191895905,1.728783289140185
1,2.8716179188874396,1.6801407717603003,1.2252576604151364,1.826309695022611
3,2.12815578733811,1.6186113782151843,0.9866689414247029,0.8421196579233987,3
,2.0747077923742463,1.7527166395576412,1.3734232380816604,0.81806725986234,3.
35,2.2783765579242563,1.1586051052930737,1.4007018640112585,1.416471483276487
1.5704489501625827,1.8808707591989284,0.9217209268734554,1.1165427047100571,2

6,1.7494250531341191,1.871843527745232,0.7768277972350516,1.7597919543000806,
2.6070504246395556,1.0292472830345591,1.0923503285933622,1.8500346850970169,
,1.6241980018876898,1.8258167505647627,1.4123633456166909,1.0301320689540772
.4047281641995824,1.1288698674734452,0.7106621890056666,0.8553058384617231,3
57,1.8680236645219814,1.335118854259142,1.1229665835634828,1.670788272865792
0839434210629646,1.7435082562916078,1.1315602200925206,0.9122090533222041,2.
4,1.9330406051560498,1.160759896048308,1.233113022415281,1.7339214777705176,2
4,2.033509074697424,1.8179670241190622,0.6315676851272598,1.1356073804731444,
5,2.5785688577763684,1.8321341779577418,1.2158249646820884,1.501324848937205
,1.9456825734347625,1.5074677337608362,1.409032520665641,0.6433029707520023,2
5,2.349606960445346,1.0063858717168337,0.679683108870242,1.4357889870148595,2
29,2.2140756032986495,1.2870381331749128,0.7375433249238776,1.17765151810584
,2.4955067480439723,1.6169269183757422,1.4713950940416396,1.3799126947632112,
2.905244608987137,1.9811861780274236,0.6809769527094898,0.7520213116172951,3
4,2.5988581458153757,1.6318135270166683,1.3543850933695791,1.605310617416273
3,1.822410567886135,1.2598035810641597,0.9922778564480348,1.794195616314843,3
6,1.546774702591927,1.6340057030996114,0.747231074403177,0.8251097001361635,3
,1.8933960664497376,1.5399853797158554,1.3707499012725113,0.6435718332802426,
,2.392616896050382,1.7798453951511437,0.9453052550026655,0.5354578790695046,3
,1.5771387202263756,1.1069806388269585,1.0148173539296903,1.462957250664899,2
,2.2445493708018542,1.7610279025020281,0.8592333693997657,1.410641054133171,2
.395264273375334,1.5412665786761104,1.0929508514349275,1.320046119071105,3.68
01365836225441,1.9629920038589947,0.6635238725850228,0.8479206441739631,2.07
,2.6563683056187326,1.341872166038686,0.8910815366517607,1.086358993858272,3.
,1.6598973797006578,1.632621893133948,1.4694123223352875,1.3917145027792595,2
,1.6127066726037131,1.9320281055100175,0.7581334327011275,1.245150288288754,3
7,2.592283134305405,1.1025097279906793,1.1567366645412922,1.981678280322331,3
2,2.2432369743092977,1.9372284872364511,0.8251900642246949,0.704659628835099
,2.5326035946416043,1.6878857223008135,1.2734731256866008,1.5427168310713908
9,2.1522410079056193,1.0678370591051718,0.6308736607183498,1.106478152156438
,1.8696030498586602,1.3009635669468163,1.4698210450785447,1.1422994223884222,
52,2.7286534765112993,1.7081720886452816,0.9537895413836392,1.57639664848726
2.6991238184534687,1.0673506014687717,0.736050463346464,1.5386542269865544,2
1,2.54204470628164,1.5821704601762732,0.5734967473300127,1.9868839905532825,3
,1.908217705844944,1.3458830569529567,0.6697579050875407,0.6925914341811878,3
2.3853460003036306,1.620915517767477,1.0197739485560176,0.6561644740762496,3
05,2.0414608454100405,1.045742033812513,0.8370031764312863,1.5865082256931118
5,1.6373731099899511,1.8715368061523763,1.3288833658826094,1.367580375509613
25,2.875970363193364,1.973488969177397,0.9308875236618059,0.9112409998397089,
.705227946378442,1.9688778552856916,0.7487142725876299,0.6191290534834799,2.
3,2.9253560307312036,1.7496518317429248,1.1171449866040302,0.628487374394645
7,2.1690086594369333,1.130086240130632,1.2067772168854458,1.8412863119961775,

factor_5,risk_factor_6,risk_factor_7,risk_factor_8,risk_factor_9,risk_factor_10,risk_factor_
,2.3837346494374856,0.9565013982994801,0.26170568373590597,0.976800328510659
.6467431242310413,0.737508359459007,0.24697879907199982,0.1107205933263156
45,2.453312804453976,0.35325140850206416,0.9062545805210367,0.4225484283119
9,2.709992611663875,0.2965355812876552,0.2495461998495061,0.042024675171405
,2.1388476854921867,0.34970322974584955,0.271949726128631,0.739904120766930
3.0381195818165407,0.774653531181819,0.7593982624179637,0.9180765521991506,
7,2.13522512718443,0.6613706110704144,0.44973984245054,0.28003732955048943,0.
8,3.600713015644723,0.18519556786746794,0.7767105569551812,0.858341676799120
.467424163820866,0.17410933533408435,0.06536615756438524,0.2922182304618977
.080023829209021,0.09839564971339732,0.4875711936733843,0.9107701616439152,
7601581751229167,0.6603027197013381,0.03361360018328263,0.7539619035967839,
301754738094388,0.7643726622217359,0.06265320345535452,0.8049067768414386,0.
,3.0659155731026706,0.265046428325004,0.906437453344411,0.0180065556004545,0.
2,2.648667506230751,0.020944961030912812,0.13924537111759516,0.962810357074
,2.6660038261163956,0.08217166747642735,0.5324206822752171,0.726670992070
22,3.3389739103963803,0.9678600348450305,0.41109560260070654,0.304751095386
5,3.988278722442335,0.29544477747742026,0.3473433262588427,0.829394664858846
.323678379698757,0.7692231478381336,0.8998333456872725,0.28152119841492995,
.11556683474005,0.6246635688542638,0.0218233967754895,0.872753839566128,0.03
4,3.4613010204784924,0.3819396396528959,0.6637896861755891,0.112580390411937
.930411224567436,0.2056872621955551,0.9633944342135504,0.7036859774607958,0.
88,2.1202846852015704,0.12138641815940643,0.5601681834618419,0.5406984766449
,3.1245936317558196,0.6150129680388666,0.9368224620330918,0.0965344701954408
3,3.9152505690107606,0.774633780637788,0.05225787928602377,0.241893828258838
.3506058847153195,0.6439042535411307,0.4187933190741614,0.01240403627731479,
,3.3800097816413315,0.5303021332491583,0.26015779096205116,0.468767965464505
,2.401867377318113,0.041951223431636264,0.7308209649807298,0.301265260760000
.0716553689461623,0.9684887775943838,0.9812970904972467,0.5983574842977588,
,2.1933528992934392,0.7987141916545746,0.2565300624442478,0.2972378077086172
.9007418725163703,0.29282203670096474,0.6541746014740745,0.2999191115069847,
8,3.512326657790492,0.9799703293588506,0.19809763275974013,0.743193198731603
.695143048477964,0.6018815896363221,0.5653302545716099,0.04814283597835345,
23448974436,0.5824226582072465,0.4639324866883362,0.9028949951919872,0.9840
3.5908999215794584,0.7480731794295321,0.9720053296315371,0.8522638743911815,
.8543556389589053,0.8117697880901376,0.6085272726964666,0.6678046832264065,0
2.469284163710907,0.6564786073315027,0.3495063720868188,0.5932215701073843,0
798631831618616,0.12809574636620003,0.11409578488793337,0.8923024981396522,
3048320266608346,0.3382675059944984,0.15124682873493456,0.18533017952871444
6,3.9849670043167507,0.9280836403260497,0.2253169299512351,0.078969162190828
3.854001935848591,0.22461561184385193,0.250966661340298,0.2395101699095381,0
7,3.079914260561119,0.3721670238375133,0.8506160578411378,0.7945782787001903
.6840665887528425,0.432076882944196,0.5612227882825249,0.03467026672648332,
.041915954544084,0.4394049896944522,0.5233909113743664,0.5828068350834896,0.
2471713760055194,0.6129395824945255,0.11476887186921747,0.9954375161913099,0
,2.1782488642320335,0.9430758377447076,0.860139693349435,0.8556960938375394,
.510540840993616,0.24069271183250496,0.7228143032084837,0.5214456890435895,
,2.25542696727047,0.12150137630091806,0.06766836190000158,0.063640909286642
3.652135260505057,0.19747048985993731,0.7078350972297687,0.8313735143857098,
3.564056175340772,0.8869249002092695,0.5435382173426461,0.5989785081772105,0
6,3.417489395485953,0.645810812655206,0.08172534574677826,0.114932997059566
,2.0723207610495242,0.28590678923916013,0.45830064152865646,0.0938572848233
606256711890657,0.8159469389686173,0.484696287130047,0.9096268194365924,0.96

.526225139821686,0.861369996363436,0.1657745401632757 5,0.6692002629380784,0.
20272814943038,0.846514338690278,0.9456981470002823,0.8292867993167555,0.347
2,2.175285493527677,0.9189265336395545,0.8499753714806619,0.878978900282267 3,
,3.873915646062126,0.2522410201733616,0.6690223373291083,0.5717723520483222,0
.1076044814247528,0.7550419287462783,0.4622955610006072,0.5174463517550413,0
8,2.611048621316047,0.46053949727414,0.4117655415845085,0.4304274103209902,0.
58,2.793963034148567,0.8419985528900615,0.6509734671509542,0.316946587791119
.8944050810096713,0.7284906771616132,0.5454318694069055,0.4345959608587117 7,
.201188666721235,0.7764474464997246,0.0622731058818856 15,0.7738796557301564,
0313588553929955,0.6561618376810715,0.512502648770331 7,0.6019234310151316,0.
8387839464288347,0.17742876946970687,0.8064036172328395,0.892523289954846 1,0
993926965811660 5,0.5450269188969383,0.4592398775969580 6,0.4433800143791877,0
160296466253,0.9846697395245082,0.0519565775542684 1,0.607089578718754,0.6206
.7028499154901917,0.9373880664971189,0.7862781993840331,0.6313075530919887,0
,2.4170210287146716,0.04317373579701167,0.2013637821359744,0.591697162306325
3.8611904292878325,0.1648148157294371 3,0.2586208348880388,0.702633773085516
7,2.232732795770952,0.1317287734545619,0.1647063534322959,0.237433468374806
.634899416982383,0.7259799099852955,0.3302150648714975 4,0.5123637775457907,0
,2.76124658695316,0.8177853298569451,0.756751505688635,0.1042248028083476 7,0.
7559486408901392,0.213511366310671 9,0.5193858595039373,0.3845113276448125 5,0
3.736113380214901,0.505852692951829,0.2048812741507125 6,0.487667068621264 4,0
3.611850800235242,0.8407030280112,0.8778300781520408,0.6522242595454725,0.722
8,3.5800608726141228,0.7328015447662506,0.8795818549515292,0.950531052586203
.6093582781960816,0.5422372054918483,0.8705784250460069,0.6006510665483559,0
2.1618385661025044,0.5903476904587729,0.2387962113815392 8,0.743593916176559
.805960357530323,0.50836054691628,0.4512393441809881,0.5062660594952028,0.79
.347049029973017,0.2975484513188206,0.9849896561408751,0.634104031376331 3,0.
,3.3899021778049234,0.5650219928590161,0.7720124763099904,0.070932216133773
6921994530755713,0.6888853020500907,0.0271674212255598 18,0.254391580841847,0
,3.95122040171983,0.8733229158608077,0.0652045912604920 5,0.3618530932051387,
.281944154690664,0.6362913539792481,0.4639313768123327 6,0.4724933550065135 7
,3.644961127268722,0.761121537043158,0.9092202058615602,0.045648745502383 73
.265049345483697,0.160071634954573,0.5387017979527142,0.1400240975533176 8,0.
.724028964545245,0.4615574744197899,0.4978125080342902,0.2768143077913058,0.
32,3.8455143810267884,0.009331619827093296,0.1054736999566305 8,0.97153269090
,2.9741238376378716,0.2466788645216230 2,0.656780104724294,0.331347007103050 7
.2125058755019547,0.7264617151130048,0.8221031606002255,0.4820410581120825 3,
9,3.529619601805991,0.9918099513493635,0.3804200075075623 3,0.196097712738392
2.349677254520825,0.0991780995872159,0.7756118509253275,0.6107800710757683,0
3.005132152926885,0.4014943158612882 5,0.9644766539008116,0.280683215515974 3,
797326055544626,0.8000709686991595,0.2037664591276891,0.2069925784384267 8,0.
2.2927479843244982,0.20403563462889884,0.5233296311947413,0.516572628445979
,2.735068844583063,0.5550849493022862,0.2871379580845755 4,0.005505799160420
,2.136344618770987,0.7330712960296748,0.7928544190338394,0.007664032649225 32
.051623813898974,0.615985450260179,0.5775933658811575,0.2190688091018577 8,0.
2703325783096453,0.1880247346154161 3,0.6345824175503038,0.0367213625008211 4,
,3.9262302231783477,0.3553845686857394,0.7979141596158941,0.108025754130691
9059071790034,0.7837917844853972,0.395970472662454 9,0.3388606493387516,0.407
9316443223064397,0.5542265141320166,0.9150900684070432,0.8025856795798129,0.
,2.864995756660263 7,0.005229613542912737,0.53302886724614,0.572048643369300
35,2.623632266170519,0.7609907600280938,0.157954822835348,0.512667725956416 4
012283725680488,0.03531135494023907,0.6958991181688025,0.2934888232081216 4,0
2.8790233772658205,0.7457337827312321,0.7932613504382544,0.9317537194328164,

211329368523488,0.2024805601652374,0.31676167706752667,0.39701504123719167,0
3.2816526293804125,0.9580734801193802,0.857179256998753,0.08709276679577149,0
2.432076365019199,0.3679407513020845,0.9061432547657664,0.6170665161178013,0.4
3.239175909795659,0.3269316163380791,0.2769044873778089,0.11383839347660141,
004021847963068,0.14888805033244468,0.9835214727152221,0.3452231428016921,0.8
,2.304049706257296,0.3056042154902149,0.14071152804875842,0.5074119387178089
7,2.1226992542213363,0.8766507618282006,0.20201566840248997,0.87422252468517
5615231721368796,0.9963343376272473,0.18422483923802613,0.49354658034011867,
2.91960084753864,0.36830953050684834,0.8939897100078704,0.7022587660316643,0
2,2.1163275910109514,0.44861063095454257,0.6542925523249619,0.99281683899907
9897326343395343,0.722070938202737,0.15210428322227287,0.13148911989798162,0
6,2.1155611219927333,0.8861957804650745,0.440323418368349,0.2747315255195195,
,3.3900704577171066,0.5930443341867842,0.615298025069802,0.3945760755976464,0
3.9673578505268274,0.39152569817782934,0.08346408498938329,0.42182817853883
2.4783602084318512,0.41262184084862374,0.8824163684073387,0.4110210752655457
13,2.2844987373743924,0.6956181464968069,0.8036035354415352,0.907611002556554
6,2.242769878844858,0.0032182636042786816,0.5052067735766382,0.71403264446685
,2.6065502951351913,0.6195893374598407,0.9671266152591704,0.6079052140907037,
.2020916220212934,0.3554930104413574,0.4177609792750997,0.3093727178627821,0
4,3.384322683388264,0.7941973312770843,0.9841103190364978,0.8237903842212947,0
124583596260125,0.09299063982313216,0.6679199988036271,0.9550605114849889,0.2
3.0188442562995794,0.5882022669483905,0.6346712732539415,0.8211982825502265,
9,3.9933937077059536,0.48097288988612374,0.165954895295785,0.0015651051914729
,3.6279405390120374,0.6423255259052274,0.881927758304644,0.636401370183407,0.
2304388764620215,0.06485359448296835,0.42748968792061515,0.05113538144210072
.6125072415732236,0.5799837874281313,0.16223340578247936,0.25760722033372185
,3.247791672583277,0.5614845879595237,0.01260751997575904,0.0595247003477716
0540829278233033,0.5606600944161709,0.5597556836383826,0.6037954529493877,0.2
2.852166767780743,0.6034876665964548,0.5273996221226763,0.6865899134926068,0
,2.261420753480399,0.6764679372263622,0.7193536223691586,0.11448763806590512,
,3.7732084304809335,0.8049889996807698,0.8902580530178021,0.3838388830449795,
,2.8995692976426453,0.26982072012821434,0.07940658464302952,0.456236508955542
3,2.3892450236415304,0.8250494101800621,0.7314964903403623,0.3690530217609166
2.7355187017545792,0.4982556822634536,0.18741194409758222,0.12102500048519305
,2.8282595050578543,0.07705827913433116,0.858177053157164,0.4189477901874754,
55075791133749,0.0585509292439792,0.8190635138616561,0.7511780478084181,0.658
4672287611228043,0.3342383186960608,0.5407937549855444,0.07100766453325547,0
52,3.5386097872826796,0.7848969771114676,0.7102427986233115,0.080180014081903
6,2.0220625288572944,0.7076809347409158,0.3143501131837484,0.3547712918359842
.832307996359523,0.7886149646724064,0.47116789893281463,0.941725711618925,0.0
2.9626883032706472,0.5172690564416094,0.8216368977104717,0.668572646722914,0.4
2,2.0383845536461136,0.44019899795549056,0.4592651971732139,0.678669956672564
2.5196264174900684,0.14745252670628395,0.3577982653254418,0.3619199908378649
3.5205796462442702,0.32819275360931155,0.4942124472502999,0.5936607912085384
2.274219411457998,0.43401936617387527,0.8282492524372184,0.010131184027743378
0706202629051833,0.08860042967961723,0.33520804737087273,0.6360960464095422,
0403744034475,0.22061195271121126,0.1737615931357438,0.9132869475762367,0.110
3,2.0242415492877868,0.5982252940637388,0.7120140203307808,0.612573468344819
6,2.482402915199083,0.7356631142391898,0.8259782127080818,0.873698593466345,0.
.9517475098201076,0.9983475113929496,0.10063747852353333,0.7239730554660938,0
6030742237659794,0.9331133313435577,0.23987411275594472,0.12055841640369025,
75,3.919153288642067,0.6425651995351915,0.1419719897609939,0.9024533072858417
2.975708087852725,0.421248053294072,0.3479414115821666,0.06644384435577488,0.2

,2.2194723973550072,0.6361773647082327,0.45035097890541265,0.533967472547763
3.095918977947386,0.785651618080232,0.7488263369633065,0.14213712379345766,0
,2.9087546691199218,0.11833619092338732,0.6511475848816741,0.011706610730904
.688714165479232,0.40990488969628824,0.6209285674784312,0.422034260127639,0.
3,2.196165161568489,0.8398022856356172,0.3523749466096544,0.295040707654156
9764822740435566,0.383832951933044,0.8414475530847599,0.486000493797603,0.07
.3000973293879685,0.5718722712271865,0.47128716739816456,0.5771998867345508,
,2.6493518537801197,0.5877693608621982,0.9791049380151545,0.043739072308947
7,3.474714154775599,0.18447625313363936,0.6341421382957368,0.123003295123745
.9520362587375333,0.3622354416522884,0.12626474156450274,0.5586423964504253
.751776570291539,0.33451128874193214,0.676177562075434,0.34316735317265823,0
36,2.7889525571400706,0.026196708532064794,0.325104015431699,0.7291734875494
,2.9188935368119733,0.024191763891355245,0.6863271966860974,0.652291648591939
.5700330876741138,0.8316970436757735,0.06964114816240574,0.8456043629899068,
2,3.7841693778804686,0.2730708099728124,0.1748814371759594,0.692492826669225
.9106693730078566,0.5180787662388154,0.8557372589057863,0.42993107739014813,
.57380677251059,0.29872557358877305,0.2271795570235149,0.6729666223944324,0.
,2.6308137914960255,0.9406792451153405,0.83704112524294,0.2753811742554185,0.
.3762694121730843,0.25929675886577375,0.27927608845441043,0.306313498778212
.875206248110784,0.4296568127389351,0.6428819100587077,0.7889851599207933,0.
.509341244092102,0.8727302504846018,0.694150573509831,0.4464195164015822,0.3
17431588518454,0.8419335667918086,0.5126547531450529,0.7983803405183525,0.64
685269792465,0.18610141784627732,0.3053106766967313,0.8224223736682784,0.122
8035239842708473,0.8026433097957104,0.21264437637109612,0.8575623376612838,0
.9229549292494683,0.45818688673789265,0.03318935456242622,0.916635235841207
.274402953616561,0.4829688720853552,0.3039464012407238,0.43098522051329624,0
.3187078444115654,0.13347997241594378,0.653162790531283,0.31886681832113883,
7,3.790235481413073,0.08060151375712465,0.9383048184484827,0.582198746594113
,3.2733393643344146,0.7279393069737652,0.871204482263052,0.3711724234376276,0
2,3.2278671695201235,0.49646115235529975,0.7660647765353026,0.601074166800233
,2.1333040811213797,0.43685070261517955,0.7884473432532987,0.705585862643824
42,3.036816043890408,0.7295082286523967,0.6649849034759953,0.688405018492136
.300338001634083,0.7655128989911097,0.260286857566003,0.37455288702012135,0.
.474867537442971,0.15890816766935356,0.9071950307058299,0.16686020169875282,
.0244438416253487,0.6102251494776413,0.670732311905834,0.43052933846220753,0
.3604555584167066,0.13535408227768553,0.56044063098044,0.14259414408190874,0
,2.0833458012047688,0.7513750860290457,0.11098946921348107,0.890097035183808
6,2.169584032091606,0.6569551562671395,0.4470555438064254,0.3458774607378574,
3.4326467761253645,0.956614621083458,0.4603582712019263,0.15445974722332378,
1441686719973467,0.06895801635642118,0.86456360952267,0.025447759905750877,0
7,2.1425134555305174,0.05705472115125432,0.5466482201877885,0.645822386286648
2.024216950469775,0.28218707469320015,0.38040054897040965,0.636902317454743

11,risk_factor_12,risk_factor_13,risk_factor_14,risk_factor_15,risk_factor_16,risk_factor_1
3,0.34060416658129167,0.6295576968917366,0.7925951162883833,0.6727029942088
,0.071711888847651,0.05433203482290383,0.9078987937796194,0.7966813971913723,
46,0.409628894895232,0.7486452341262,0.9437015928202208,0.250467898792499,0.1
9,0.3112174950633936,0.31758679526111666,0.9601356044054472,0.6248740996069
4,0.6771206397350094,0.00013469300448532007,0.5214596387214719,0.5717459831
0.6057792494526903,0.5111291392372298,0.9773079079915037,0.8328303767882711,
36459372819754,0.046851908527603126,0.7573102071344389,0.9060870604812605,0.
9,0.2178925542549901,0.27616955782089947,0.1616714362369619,0.01215677147738
7,0.9880359661555919,0.7069764874557162,0.47690010646797,0.6740199190587927,
.45400162119611487,0.06268962162186587,0.718331175372004,0.051835799119729
.688274235780237,0.8393384923560023,0.24734031581908333,0.5488586650773559,
14055296757836078,0.003819922658583108,0.6406151835488874,0.287632729043678
4855896996593978,0.24682386920190225,0.6666004790466192,0.3067765995221412,
26,0.027536719869465487,0.7409040561520714,0.1626997438085659,0.352958503452
33,0.50545511791045,0.3162701497293068,0.5650853124527563,0.6212924490496101,
333,0.9640174071177023,0.10189238806601353,0.7716269109786077,0.334049965668
9,0.3842067129858002,0.3602339188004279,0.4988956645359419,0.732699050590280
.03899601257491159,0.27039328638452553,0.012103116776107425,0.404527385573
95579091934353,0.8427119021605549,0.009038469079292177,0.06835320030995862,
01,0.3879867599468695,0.31334791758514313,0.3570293439700122,0.783759842484
16002493646319405,0.7889324717760072,0.926194068431782,0.285758324592706,0.5
103,0.023352096328185912,0.8918721448107347,0.22867677960360877,0.4327668795
1,0.7562136139501768,0.43381741957652953,0.6343636888716871,0.68544388322190
98,0.4585230088818907,0.909943143854392,0.22207587069332058,0.33245615714871
0.2892447719726545,0.37731805396319285,0.3216699896595486,0.056585656146715
83,0.9000831830164807,0.9640784164901082,0.848042089601484,0.373921047012813
4,0.11613896020052561,0.08928952452557293,0.7288614422699469,0.9444485826943
.9559188289553192,0.6870192665032427,0.09539927731321063,0.6417343972985582
,0.3139667173773528,0.49381404045692867,0.4287020209864336,0.671479148667206
0.8884036723086812,0.38764880545564706,0.029169763645264468,0.63228215363763
4,0.6029428764794615,0.6327118787486009,0.480890146603009,0.1989921389594368
.8267073597414875,0.7038570916343312,0.6624340117010681,0.4183386410228013,
131632104643,0.004363269650309087,0.11850262359815156,0.7509397983633183,0.2
0.2884054378187457,0.16695308874719872,0.28888493890916567,0.101372897553159
.961135485407593,0.7130460506343187,0.3978366255021569,0.27785277608971226,0
.38948189490242036,0.6663854135782065,0.9195310246560497,0.2763191084658536,
.3855119047369223,0.9660475051286772,0.993255015422964,0.432018927594506,0.2
,0.3403871042545057,0.761037790250783,0.044911298995615856,0.980368740942185
61,0.5414076073475852,0.950772707731338,0.761007923495392,0.0675025466838219
.1541450431784871,0.702540501534807,0.37172406640234323,0.518700994380595,0.
,0.5537114853580112,0.29805220863944315,0.39245818622948003,0.17936468201221
.541704812410004,0.10536492225085647,0.7542649733470227,0.9706765454097441,
76190181260487,0.7818238633449356,0.9184264321454086,0.1133035047274642,0.54
.8336499619375856,0.6441492214759159,0.9509285945025484,0.40360100236159124,
.44044135031242404,0.04818601239329623,0.5771225860383208,0.737884984834606
.30233324935149375,0.36005046590097833,0.3571211593005028,0.704554428027445
4,0.25933489731709447,0.9567998154842862,0.7875490858587793,0.42272863689393
,0.19462383670487193,0.5004011457102012,0.25100138326230026,0.346524167952718
.05764758219902488,0.43272913601970464,0.5640740288335178,0.3976123785528705
3,0.34244797883471334,0.457699605159294,0.35857846152487316,0.26427626315863
62,0.27024769213078326,0.20888272400516372,0.6566310737078761,0.205332938706
64173858664074,0.3687071708647952,0.2403989128935069,0.48303969755889,0.1153

5577088262500439,0.3698132559871766,0.19159294573482577,0.2685337560676073,0
27703903050755,0.0523550201874724,0.9182390866754371,0.2874616648972501,0.11
0.580467600993696,0.7675676285179183,0.1018036829495369,0.6567560634745571,0
).1391967013456955,0.416504431903879,0.5059579077349317,0.9685373316998922,0.
).4440437492022029,0.8221798868164233,0.22085278296300892,0.6036372005593751,
5262343214210638,0.8503480011198954,0.03892996332566745,0.07697946706496628,
3,0.4888978317924514,0.21199405469865806,0.03601881829142639,0.07558361385406
,0.40177880065432425,0.6573534821701286,0.17522177210582734,0.95142322084556
,0.9938483888996982,0.4722818041629817,0.8667703545060003,0.2972907903073336
380314762148927,0.8801567379214851,0.28247874397702666,0.09206698608247543,0
).6234059581594401,0.21574067917645412,0.9504591705752957,0.5990445871503816
).5693773611822828,0.6778132003100442,0.5816222929891935,0.6236487782902661,0
50479824584,0.6077521222527226,0.4366141897687661,0.6485048201186882,0.79275
).20131210782243325,0.2953014775718328,0.5800888346694546,0.2674020278575413
5,0.3951377432643186,0.1366009036511726,0.5166983852262678,0.015110684214475
3,0.03945953289089932,0.6516397604772766,0.7587762537573252,0.965015369454471
8,0.47554603748682667,0.7385974581323307,0.28249606597285226,0.2508930456911
).5430245839576632,0.31562734376335,0.3530503916003024,0.6760262880525475,0.6
2277497361032096,0.6448326040223138,0.8940943025332507,0.7066298698787096,0.
.9640288471655137,0.39513090761150327,0.9464565131504055,0.6100074170794857,
.909454610937242,0.7132068832826177,0.8925582274864745,0.3129073942136482,0.7
1432354559059,0.1992152748940531,0.4194479939573136,0.27109625376406576,0.53
5,0.5333866287653626,0.8902150026361744,0.7803655030068817,0.597668282702703
).869969159647685,0.28740959646557973,0.4763357333010988,0.8660956336116915,0
3,0.1306523438234629,0.3677859877561529,0.4975398521461022,0.9467337289233956
0509794274885,0.058091952080349785,0.20468010905728395,0.10590580440375763,0
1248208139668483,0.1115122123212764,0.5911303032585165,0.15482861259771985,0
3,0.7942106625494054,0.5158619027123281,0.18613589775334793,0.944736258783079
.27576756825135695,0.2675926767279715,0.33156469906719377,0.73653522833347926
0.8770905810181889,0.8354802257421944,0.8550302016199351,0.8829938248297731,
,0.9440420938253978,0.014693388654953843,0.20707580921085522,0.2026326341353
5,0.14879596940511564,0.3790940486719362,0.07115828517343281,0.58758587110940
4626756050653935,0.3373455467690927,0.0690075926128163,0.7011395944174736,0.4
9809873781626851,0.019327630917601035,0.9407845205031079,0.6801118854469727,0
10507,0.4834074359731466,0.12436839900051022,0.5069204379618346,0.4081516908
6,0.8635475225145451,0.41364267491863527,0.40941207181701833,0.0153948916913
0.5887315221313199,0.49286458444744663,0.8108785810079184,0.5829260568042631
94,0.37532990791960186,0.4042897472548691,0.835825927910516,0.25310153917960
.2857835555258702,0.5309376784120938,0.3321910331156046,0.45025424614289034,
0.20322309108608727,0.5951316749056815,0.6936180574737952,0.9575810150838173
7617981913776972,0.00992382112783563,0.7711228158518866,0.3990353176424347,0
1,0.3865405068230279,0.4640951594535039,0.6546506868659545,0.8398016066644267
321,0.5112754622007512,0.9634992408875883,0.15159507480009937,0.1885406057492
75,0.4923254800011819,0.5190322801295487,0.875883083517057,0.6724604980250288
5772790292331312,0.6775422674589532,0.5390945527360087,0.9770069544389435,0.6
,0.8655771456245522,0.3118638579168832,0.2824724263700037,0.10189310087293679
8,0.9807393435834294,0.7739913781527625,0.4252281854376939,0.008319942227192
58420857381694,0.7729214818761151,0.03757109039890738,0.43358279263261434,0.8
3275189221063031,0.521296047654139,0.12786680122241756,0.0926254830513672,0.4
8,0.7645277946547177,0.9760138232792136,0.7655467505158609,0.7483843496762314
,0.5735289514555733,0.12555021476265338,1.1634755366141114e-05,0.914548663848
.9560471436119331,0.016947664889580993,0.4165658284822922,0.4340209727087588
0.20047451552840279,0.770158211757096,0.5225101810855884,0.25871164215544096

.10926421030962141,0.8071555373648825,0.054634420000972295,0.43440331900660
.8539619669554221,0.12020722912057624,0.9730781629723328,0.723447817850001,
4391498166337483,0.2655828329586061,0.22612533124967193,0.00905416158037097
0.8469688542070605,0.01755236336662891,0.30419871963628087,0.58945398948469
8930902051954149,0.2933086107805647,0.30394251225502733,0.6132916848873791,
,0.06245759361907144,0.7731418739186715,0.2304166621173499,0.637688246895935
58,0.8834647629860607,0.5179605277125789,0.001473822086311416,0.242022079980
,0.4483190553694222,0.3480955931682881,0.7293447940879964,0.7140527233781268
.5104324890042322,0.3717392949063303,0.9668454997992854,0.0913914863317764,0
04,0.6265925110932102,0.0013536257154681541,0.22429348328504506,0.1992703364
.9263826067300868,0.2998005275652359,0.6630471918034224,0.8774700466544819,0
0.01911609442976403,0.6464587416379305,0.7418963269587151,0.738723150726566
.4768434683178171,0.9741944438163571,0.8484253790905247,0.01374555401134525
137,0.6877220424305607,0.8470608269838995,0.4226291970136091,0.2483762603698
7,0.7227069576982285,0.023594964645529126,0.30293090564042924,0.214406482993
49,0.692532431537108,0.8985606046748393,0.3252951340230311,0.270806328036972
564,0.13443512940812186,0.7831984132986259,0.7126213330985128,0.247559770279
,0.29939558837168123,0.7803761235709804,0.816779466505636,0.0625262957428457
.35871552541701346,0.4579957590035715,0.18161396775660266,0.458940516596820
0.804437207679505,0.3980052929545028,0.3709410081577652,0.7327334747861651,0
278759872414553,0.3029157288331926,0.90194019674915,0.6067317247174364,0.751
0.21070496386720317,0.06569485895436578,0.8066935196947392,0.67287166859807
9042,0.9574480190380962,0.2282353634936991,0.9848584027624758,0.081149178536
0088632851134689,0.24660363497802462,0.7542482596820808,0.9514907101767872,
25,0.9978208556819782,0.4840770697102016,0.39319520102792227,0.8384918338430
,0.6768212988573956,0.747444406596694,0.5906378339946378,0.8050903184956627,
86,0.8284692994491171,0.4738098283979466,0.6610142542473203,0.82298380504176
29461939634619316,0.057844449447160606,0.07845581631712395,0.93271050813276
.014315277667059978,0.9577898009882039,0.5444969591038651,0.544253966113697
,0.73786951856153,0.9427243190941123,0.7093208284726096,0.2002820178676259,0.
,0.8341445970006758,0.7851895431484084,0.16732130400106038,0.616783607852541
2,0.7404761319535191,0.9913279015642206,0.7806317174255571,0.742881493667939
,0.1428502873008386,0.5444823328002629,0.5837727805141116,0.737917393354025
,0.7534279222569691,0.9627683502532061,0.9522217856975946,0.521449024166880
,0.768922782107641,0.07560504061675077,0.042422298206891695,0.0684587193335
5405156700549,0.3656807482613713,0.26532613755546697,0.3711129384301278,0.88
.7661159327955862,0.22538126800676583,0.6015538792998241,0.9207666084633573,
45,0.8459214396777321,0.19583308413427003,0.2965599122059307,0.584448909757
2,0.6136041142374584,0.14080294889267608,0.7144241640712422,0.53833194325571
886047139696 7906,0.6224136415659967,0.759005282521931,0.2688535143821935,0.
48762780966721087,0.7813218557473051,0.10251596406647445,0.3684489199165789
8,0.07764933467160506,0.5782983994203607,0.5138544642067677,0.8953460891531
7,0.40754303103243084,0.14696270958820967,0.5088905555910559,0.666463047526
,0.40710648912562764,0.8111237699334709,0.36928077021554107,0.78738702675233
,0.06600984414024158,0.6359547071637285,0.9329248625696721,0.45434542306765
0.3488205339694793,0.3881657061269955,0.8275063198732212,0.6302472194561579,
99809879131484,0.674130295536063,0.6972093819096368,0.24838360874320753,0.42
2,0.8082352100092222,0.25990497924283285,0.7143266357349976,0.70546083028348
.9476880259644708,0.3451922084942105,0.4617161438131191,0.4276013337033373,0.
0.0723164752579386,0.9174858431319902,0.9209945210965759,0.4425455148147456,
0.9551152072917014,0.2905045056714204,0.6945954272532854,0.6493223245768817,
7,0.5225765993367452,0.4699882681579366,0.7289810616558164,0.936280621468926
2995656779538739,0.8901411373960146,0.8616909037873453,0.06400634660862092,0

8,0.07686207527616351,0.7077213502675275,0.274071589342191,0.824743237347007
.5006242680704178,0.06220322616470275,0.807070903866703,0.2923831104121931,0
66,0.7945155444907487,0.14730304427897034,0.19524060295649204,0.44391945450
7070864772666982,0.007841590771916818,0.34534195224488784,0.02191332877500
,0.05022601146397376,0.6309217902291634,0.3356104542698427,0.3010460778343
290183536790351,0.4476734254104183,0.9785254683891406,0.5026312426301305,0.2
,0.4028732826326371,0.13427485090227553,0.8565372237479268,0.05617607734743
,0.2952904940479433,0.9579341210567704,0.7011698615902597,0.4910957293831651
64,0.23238432305604784,0.5296597239456199,0.7270567616153901,0.927110628858
,0.2810044989087136,0.24189329411288918,0.5620727944025711,0.105393218107805
.8034827432968846,0.5006035711547075,0.9470906671085191,0.7644407280755043,0
48,0.9292280536318539,0.6796203327351078,0.49625875288236154,0.4096710533759
97,0.4051026945275664,0.07623921797356448,0.3805177098698945,0.655173714524
.9061110021324971,0.274703815047892,0.163035336931591,0.26023659798522936,0.
3,0.32149570124788196,0.8069654908839936,0.786205652261058,0.159492262541358
,0.4764369863437916,0.45966487040266124,0.7344439320755914,0.160462614271226
22602899781599972,0.5465771420322949,0.3843550385241349,0.07049194564314465,
40476042175844,0.43281600220946925,0.02519341395922936,0.18564670490124302,
7,0.9789811151748089,0.04389994264300512,0.8389973277180107,0.664219384988968
6034930960433786,0.16574977982015593,0.011417983454073632,0.881690611843508,
5781408411354887,0.4455416387418909,0.703699779629051,0.8141256855911618,0.0
78174459905284,0.20918546540792782,0.970257109726057,0.6851343098744841,0.6
92067846538757,0.04997902865967874,0.43766132814820347,0.11043186341110267,0
.8886590802653986,0.8436496640518237,0.23497340499595276,0.289187446295949
3,0.5030839507798364,0.9811925291641158,0.7048710100433817,0.309806875322469,
.44934974203964206,0.7931428739967394,0.8171281420155851,0.249956946397621
0.5858647884535192,0.853784921704667,0.5464303160778473,0.5150056597097231,0
,0.6247838627894025,0.24197713031704537,0.9670352752561462,0.5355538148796
.07177580622651303,0.9606269755131998,0.0516687216579611,0.3568859751711102,
3,0.6826172212234024,0.19692570445342839,0.5047955983045281,0.3537891587364
,0.24193168031552958,0.9514298103618323,0.7184539473225501,0.828555597906081
,0.7139526325065898,0.994819293965951,0.862640471149179,0.7892928483196856,0
225347943841967,0.7117228057460487,0.17925561190779005,0.30779583168074565,
,0.8039585075525203,0.9811437317918219,0.8000034817636814,0.9137884438732593,
.5525009674078891,0.5695397795671037,0.5527070757185453,0.952815017002506,0.
.5201698923107512,0.25954178317609033,0.3965536819899632,0.326742542030040
3,0.14287596133941172,0.43699588566562086,0.13171502857909356,0.354424948793
,0.7753461504031356,0.593560935378786,0.8652957589089124,0.5056341534219588,
0.27140938044671437,0.07308156202701499,0.15727320817971535,0.94112081253367
.49669542281794654,0.6223432646172461,0.30978785920909924,0.876319433142311
43,0.2842740929275167,0.9811778367622945,0.2900455319624369,0.102567962583589
2,0.13382836299969314,0.19010764978372763,0.8714140341908543,0.3927307432714

8,0.5533711897497718,0.06725591396736785,0.14122354337314946,0.2963576135132
,0.50313247255154,0.09491793682166971,0.5708774168394762,0.9383157286513161,0
9386306793689267,0.9988926120332906,0.1852703553833237,0.8187402294840508,0.
39,0.8588167711681547,0.32663924942591094,0.2786430631150364,0.98356720651366
37487,0.6769407829968374,0.7481839120488464,0.21870980032358323,0.2596708389
0.8379083748518036,0.8066650530268165,0.1828377798688171,0.969723632797977,0.
8587642793494722,0.8580232710072292,0.825710951560667,0.43123386979155787,0.
6807,0.7483558536657398,0.9976282974021565,0.28570756766089034,0.34751879531
0.43921060124971734,0.24149648525686784,0.9271237818644493,0.050349526969353
2,0.6105467550154245,0.04035082685969971,0.9699265253451909,0.05276525785960
0.16036540611974315,0.411191517994586,0.5712604699370973,0.6920112559466547,0
2,0.6736454174749077,0.13008066982894695,0.1430011226657264,0.45792899443402
0.17925325573928075,0.022425889162055124,0.37454824337958126,0.2265216260361
3566,0.6939495905101568,0.3604268271414861,0.797732263348214,0.6139703063252
0.22959816075396866,0.7837382510752364,0.36739072869707046,0.252858118250111
7692,0.11755130015021997,0.5661806912250709,0.08701721830278875,0.5776317543
4,0.16528449014295743,0.31277798921035405,0.5567849109281879,0.3592087469826
697,0.001992135219070268,0.6543420054927197,0.8451208514960348,0.82355222661
0.7187396178535302,0.23201818030881072,0.7959263871435024,0.820696160766779
072,0.7324010490583791,0.014381446072085025,0.17512314013035324,0.4773503981
149333499132595,0.7643533864364274,0.6727646144124667,0.3508191093350156,0.4
9926263,0.1613628010553837,0.6237430848640091,0.2205042634356471,0.362559796
15,0.08355433751068975,0.76230262450096,0.21820753455217223,0.80571452566821
2,0.01912896643728701,0.03893801668766561,0.8740717733051852,0.3281794827976
624,0.1657481769652207,0.8371196278306522,0.24966895274023015,0.2090593737068
6,0.8908555349831467,0.6195262000363033,0.2632473465180112,0.0846883418753068
034,0.24153684170168177,0.5633953507126401,0.0006533907612995549,0.466693120
,0.35427132721721566,0.6246140481145316,0.8706690341255177,0.482132098919841
9,0.10534548448133318,0.8644332468683641,0.7925948132091347,0.84090871040290
398,0.22236130474817029,0.5869365065589071,0.627328935090678,0.22123869644606
,0.5192245707802051,0.5809503798881793,0.7502657639388423,0.3809912736683999
,0.6077526830956526,0.9906791570657658,0.1520485674244918,0.8084057388936909
4535231416621106,0.756730167068568,0.45832359458757543,0.8236236107489735,0.0
915,0.05773215922511721,0.4422938054511353,0.3512193873487809,0.3846110759787
.39103286912739865,0.7073970000329277,0.09377652625701649,0.4591240232866809
,0.23420337092633414,0.3892304716917885,0.4861304852149241,0.303285890849282
1955392322980194,0.228875462483499,0.9207396503426354,0.9325548784193121,0.9
1,0.9598628027134553,0.5968518768877594,0.04005911905069448,0.118643765741580
4,0.6163004859257241,0.9281820981139515,0.2914609134937939,0.93374942300147,0
5568784431720959,0.9291395200154627,0.20754093620570346,0.6838664518353432,0
045,0.4157751035233219,0.3419052781054903,0.23814665366569632,0.531572152686
0.42894433343014426,0.527677466221695,0.9084345518793753,0.03265906078449587
09050038380808,0.2116358985698612,0.4680690542342901,0.4755961918220083,0.467
0.6964298070361072,0.9956311094988834,0.4662994129398188,0.4077481050027323,
2,0.7021460961818294,0.9811179078679357,0.7605756424552823,0.1612188119751225
3,0.17169390770544957,0.6495089297074473,0.1544922040049258,0.656409603500650
225,0.500112752076627,0.8044140596774734,0.4872653541000508,0.971150907368285
323,0.41195809503410197,0.7151001612837,0.4299852141210597,0.5621888110645251
57,0.8705269662693842,0.5931582190394682,0.5969106905466561,0.714753276443945
93,0.6314189212183764,0.053347996988897695,0.9995577032504386,0.0684504525666
7998,0.5325040664159818,0.45476434255266107,0.7694488940820471,0.41766699581
9464217891737,0.6747863021674105,0.3978657030174272,0.11635848516449587,0.68

).6056378893234114,0.6775316138836847,0.827553052274047,0.6127958355365104,0.8
720390568023342,0.3731226184223836,0.17070836331248018,0.9384821582141546,0.
.33720657556575206,0.9416919866580306,0.030463686308960125,0.661777732471638
14284196481433187,0.167357032236781,0.20444837246427305,0.0768030483144215
,0.6920736470243689,0.5003594462155606,0.3404115561142596,0.354666204985082
0.2062521138361526,0.6908839765508228,0.5105807574595599,0.5506633794408515,
698,0.391859373188026,0.6971358058918092,0.6151291405411998,0.403207756133
6,0.8956747776187787,0.6486389567688156,0.9106057172144217,0.833644010475172
3,0.2043163833989703,0.2752882134518601,0.5098540095504577,0.814671004709989
).5078050639587791,0.15620656868662197,0.5012756446257294,0.6118310201032214,
,0.419271661166022,0.6359403268267109,0.05024300982298213,0.3734579451327052
.018124041045729733,0.5993021161394377,0.0349115526562238,0.255336373763244
31135728128,0.17935601028924097,0.5511599619163721,0.10587431821312931,0.8655
,0.06904590631459973,0.7051391455705633,0.4381786132483445,0.354897683486554
034,0.4742550209769536,0.4551719917336564,0.8391803949802986,0.41317251720771
19,0.5606782949389499,0.6677605916031529,0.16067958471184518,0.67582292510407
025,0.6284264566145871,0.8373670797701507,0.02497166211704959,0.65815982429
886316293078756,0.16972842857692316,0.4490387679301258,0.0701081396412569
2532723835227151,0.019144867996836680,0.23747855675225749,0.395259315431157
0.009978776658449373,0.7791025863957868,0.04961437333883745,0.181778867217
723443662354912,0.6096974251526159,0.724598286847629,0.1568127053168057,0.59
56565993475786,0.6996395696221217,0.11159299995724747,0.8273021497372459,0.6
1,0.8361177032072488,0.8381549749886426,0.6083195000077717,0.04191521671625
).8213883629006943,0.8028109348245005,0.2810497501668891,0.41918287590212
,0.8433261677878832,0.9609201469288247,0.17349605035502202,0.1682036557665
).4850946599795401,0.5359350788521182,0.37975152291931746,0.879330215678474
.33362839583686565,0.4884956326599053,0.801445776222953,0.557040513275035
97,0.79155820259137,0.4018871998907301,0.3920935769045727,0.2313806363768480
,0.45129411302925493,0.1536910428442045,0.7507696492110557,0.50242728716637
0.1834421964034345,0.5726072328820224,0.1254862967991507,0.7314863597031158,
4316,0.8549740195614743,0.2770125243259788,0.7727714196650791,0.958118008919
19,0.8825598767723473,0.9213075122129755,0.2369333249066486,0.2204460042454
4663097561401701,0.5831908042770976,0.6773931298888728,0.8861817721293451,0.
).07569972730978747,0.5928401805081102,0.5657815548409963,0.9343041141674903
666981,0.3878260899169312,0.35492466087701,0.9291447172116873,0.9164205445
08477,0.8035376594626034,0.05198935197770749,0.3873225179753140,0.635414356
,0.9017740285462287,0.03174376537298862,0.06616518883444045,0.629820654434
876,0.20347274249759584,0.4233351441531975,0.01915548555125290,0.4032106331
0.0669736120252715,0.08456537809723785,0.826858560972753,0.752397882303787
,0.8773504531328387,0.6068469555401391,0.5247356376614322,0.531294018350931
.389404938978875,0.8809730056396574,0.7752440518349493,0.6774380712282945,0
,0.5417609642675633,0.8829148165187222,0.2344256000115147,0.428457903848795
25113,0.9680658120507589,0.6590454101322324,0.34508374551323373,0.731496464
8,0.06656472791422285,0.211231831472931,0.0301038722113189,0.824471517604549
483180074520956,0.8629968313588721,0.9614923245741122,0.14643625660281612,0
9,0.07410542257486707,0.8860934353132082,0.6678679067008134,0.832929715574228
012,0.3754689284665004,0.1966535849529587,0.9332050877593062,0.5402965317903
8038145451934468,0.7374260857706773,0.2654964074290723,0.8446659090902243,0.
4334722327556344,0.2865195489271229,0.6114636112256608,0.4313017063410146,0
4,0.9971823539029504,0.8026403123529297,0.6784999682926378,0.379040485383035
35589,0.5590592849466022,0.9972378999036027,0.3175497002575884,0.91506073251
3,0.3211621526359535,0.03002600532939281,0.848109235015630,0.2511692675484
,0.22011132590475313,0.8973658657909319,0.9471614629002478,0.844212325286143

98,0.3505205342861618,0.622630533598658,0.8848551176960043,0.484020572509404
).3725332737595508,0.9733826191648212,0.7390669759393725,0.5140954425512597,0
9,0.06871367772396098,0.4650095175264276,0.27746467972495403,0.3084682814894
72,0.36957419942677316,0.8473879305713737,0.28161604210563174,0.573042200619
).4642385197392249,0.06239406859827723,0.9633603204460379,0.3252111962762113,
5,0.7227377522388047,0.3352790500782957,0.010284495935692028,0.0392694142416
8772,0.656729484721608,0.06701653775120997,0.7160499732421445,0.271998605344
,0.7087656552291542,0.9753431334790705,0.7062461625552622,0.0063790434065906
.008363681876857099,0.8169739072718367,0.6226895409497974,0.978354787056856
668008,0.21766739958053605,0.8525474212459705,0.9901088460868658,0.965747209
.6616842883998565,0.9379652314338942,0.31187943576372523,0.395155297472305
4,0.48398936817974714,0.08510348611978535,0.3399718637932482,0.72837085433964
1,0.005313475668345946,0.38559759251333336,0.07893940294655555,0.34580609841
002,0.8044946424318223,0.07103488904400213,0.44279813654373745,0.67109315708
16456,0.7728095732903374,0.21079310155002862,0.2610485262348481,0.8054300430
6,0.5484191629935626,0.22936577703767236,0.3433867021776442,0.9467562446922
8864,0.06646332577627212,0.4691072110475787,0.8345717902556277,0.40021227584
4,0.7665150495325846,0.2685621674536426,0.9354845135274957,0.783297982956047
34,0.583713587731364,0.10097810681776109,0.18573307200518563,0.265928450632
.7819599771579592,0.167491089094107,0.37250893155346587,0.9903323037936483,
7469923958404,0.14727556355209093,0.9286305621153941,0.025769598764351143,0.6
98,0.8030020719578371,0.9730767979122983,0.06180846068922263,0.60352880650112
23915,0.5180080664046248,0.7592925069184736,0.09236052811137851,0.65950598165
).140324518193515,0.967761465269562,0.16279098920622936,0.6880455570741288,0.
248,0.6711574733796887,0.43974048650790853,0.5952128341268748,0.120387578789
0.6204737850519706,0.2783684816626566,0.15047227100599747,0.9385297834731304
75,0.7422531871990872,0.7982931972091867,0.9680763814507335,0.18118327905008
14,0.1698810060507353,0.3262539180411468,0.44671452725852034,0.6227534032398
7,0.19497519990028922,0.2993833737053483,0.5015179603501664,0.22267607912308
8904095172193285,0.2325059922078312,0.2466586158054065,0.3072164170212709,0.
2,0.7499777984520708,0.12967897822423535,0.4709602524347335,0.5464585417281
,0.9082284887003229,0.25646306101676597,0.6619136326370115,0.417193418564576
4,0.7587153211639207,0.3553197484738877,0.7511771369540428,0.1602256009765063
1,0.5971553059903019,0.6739942081457304,0.753868082709913,0.1709364024208483
806,0.6541327370278737,0.06256484221945158,0.5778911126940883,0.41814478816
912792394001,0.21050693966534295,0.9043597749938572,0.7572829782755007,0.342
0.5788067545119405,0.8090236402916764,0.8174078473061094,0.8979646468477885,
426,0.6323913243283874,0.14692192358377076,0.7570481146986254,0.0841276622593
34,0.15647671600360058,0.34264514652252154,0.05560552168046062,0.39310390755
4739212201161027,0.8646589218581401,0.006877962219649314,0.10024556372287663
,0.716397111958099,0.15492531114344266,0.21244727719547296,0.016592480632597
693,0.2709426983163654,0.0827368356181899,0.6635235939134523,0.6617186826439
3357,0.20225418052453492,0.48453799508001083,0.4111974888825458,0.6024494419
329,0.31382680822441555,0.3024195112785364,0.40240625929931173,0.163019113814
513,0.24150062799283345,0.5634083644838541,0.8845868882296137,0.233812833093
0.21491263703639585,0.8038048904834751,0.8955628481318387,0.02370775364451
2485084286808694,0.13714848815274316,0.9091752640535857,0.8349472056944037,3
63,0.9078385138835341,0.580699084554285,0.3138780765150273,0.974690596048551
.5070596467387183,0.5055087603898639,0.6910558127392618,0.1354595367946908,0.
0.18792038081532303,0.14407878601319957,0.27163318127436964,0.2314752137652
0.07697166938087407,0.6239984855289824,0.19060983307444979,0.8684095239469
7,0.6961561451379252,0.27364041786077165,0.18463245734736922,0.9264637979561
.38279876443638783,0.4878343185440125,0.34210762086359403,0.419627382806810

6,0.8218309465853593,0.08227232427706188,0.4299576197798217,0.05062247226907
.6595107686407702,0.45958405721362305,0.8309491710550684,0.0393918336928194,
3778,0.7962414505413685,0.3064634680428203,0.1198910934727121,0.57394986606
956,0.27194603941844875,0.8223812111853543,0.7349258473948942,0.39289510889
303,0.6923589224610581,0.05697872699736828,0.5314549141893053,0.028652427577
640619846937188,0.41860727491832994,0.2879453426759925,0.5832523923125568,0.
01,0.9390684794502916,0.458431424131438,0.4928195845328246,0.01130253658902
15,0.6363742815333744,0.7250060762991187,0.29994206977702265,0.78737195814927
273,0.3245118001753421,0.574287575090211,0.596081955238306,0.306432822703371
22,0.26951186229227264,0.667212337194181,0.4338439228552442,0.04025800300923
.1909265850716435,0.7770492856517561,0.16440552907887873,0.5883522209831533,
9263,0.6946409588937278,0.8622029736581888,0.11658307455569672,0.39768441443
38,0.2187156129979273,0.3138754967224705,0.5466956820721302,0.97380041335714
5953032659274891,0.538022705363903,0.9024130332861034,0.5441986625037022,0.9
3,0.26463502692535734,0.8401080784391999,0.34395768655768555,0.2752391810370
43,0.6619689447938686,0.9894511309650564,0.7330001072226232,0.70943886463397
,0.8149397285405392,0.8895193110437631,0.658661354939767,0.27149200254271133,
0.7780252431446532,0.3714672626243768,0.931554914309481,0.9042432914075651,0
33,0.7608513309307817,0.19516651721865041,0.8209810172991409,0.3747219705073
0.18772240569225207,0.4893925374065887,0.566605258905463,0.5498685627762052,
3838494698451871,0.7416414518360422,0.6570304982341073,0.05057310382422886,0
989488284873856,0.4925690707532976,0.8983864665840349,0.4261212379522308,0.3
.3683472137943513,0.4831808633136685,0.3995187845869348,0.832363539660995,0.
3,0.4323462264033473,0.8382304210818006,0.32679470585777737,0.80564880536334
0.031149092793391064,0.36140377906718635,0.010811083635196339,0.22431855885
3,0.2595764492750492,0.8597946748116456,0.8265510741914642,0.226079071010562
.03367640387446402,0.40680480095172356,0.8009807004033649,0.817305312213236
26,0.8791857726012476,0.3282331056783394,0.10445451810015138,0.9303984201693
0.24339668321853047,0.453989911199699,0.5768573811327002,0.09536092127300355
6,0.557337169573008,0.7624469586748684,0.4639816981668874,0.4500586394262252
14,0.03897932066113041,0.12600202786399428,0.11870909550911146,0.337454278964
.6668473913056581,0.19641921587201738,0.9805682270607236,0.8706884554574393,
0.32302734575409386,0.951444825663092,0.21469588746335544,0.0835249146800187
,0.8979191869576658,0.17549162738135737,0.06723418009056115,0.211125942558037
3881181889340358,0.5677273179310546,0.5948473795019096,0.7524683375864943,0.
3,0.3252906183617478,0.5793144666619379,0.7388666771330327,0.051293848981215
05486,0.9009608956904679,0.4896244620502448,0.03244344604718696,0.4925353807
.9961576734987788,0.6447452485768186,0.6587176387270222,0.44210703467972,0.1
735,0.8254156641239481,0.22982672579761843,0.5322918759514544,0.3344011789887
5,0.844871347755229,0.5526622677767482,0.10326500887504841,0.394572315318923
98,0.2490087361090899,0.37217325524689715,0.17252387694636206,0.5299405869302
162,0.5767023988577563,0.661667259629189,0.5679742973160805,0.16136735838838

884,0.5719958782835928,0.8499593305032054,0.12328377929894685,0.198631613861
.8054323292999863,0.19624571670616564,0.5185616886657339,0.7833872199958384
760160929790159,0.1255497898984771,0.2458474424934003,0.9092526944369924,0.9
3,0.15389990469377368,0.9634799474743275,0.35813971652914445,0.370232185684
50414,0.1492494697414739,0.10804455937640711,0.9895053333843224,0.751278924
2681743677449133,0.4784910701071891 6,0.6843142489253448,0.8969952893644111,0
3610747263410372,0.5855637287933019,0.9489806670470013,0.846124210530231 6,0.
37546,0.4084555814084826,0.5413744209074383,0.1425565550331156,0.18281338853
24,0.6796972168333453,0.0860321570329432,0.3821394748325692 7,0.9562918815133
262,0.05668043216583540 4,0.0570269140036193 7,0.5547318032823543,0.376676440
.034672704226066386,0.10548342746103567,0.07675806188873802,0.339240661173
79,0.391910562943909,0.5855876838726125,0.004187443627957865,0.0624313272777
057,0.6971636921379805,0.5443448876343211,0.6704343337969175,0.486173654104
326,0.19343534295648257,0.23374079203674425,0.6418195168014353,0.109013615640
2,0.6415044812562517,0.6377741418455916,0.41078880999961176,0.24853982092742
444226,0.25982813794029946,0.8200675020556494,0.48964407603203264,0.3169426
9034,0.8860861088135163,0.041578959846593766,0.4154430005920896,0.8088263234
1819,0.8956899442666165,0.4981299435480424,0.024005044844888856,0.875488650
,0.2972872042813265,0.6893104121026903,0.3353716062174624,0.302466317461469
224013,0.22999375396 32405,0.2523119270696871,0.17708225539728983,0.858698121
1130397206623126,0.3077709052931491,0.09842418910136874,0.8572309373697911,0
55909454,0.24053158518014872,0.6136379029893838,0.9567204338893498,0.7646509
79,0.6723838432917036,0.8979352934437369,0.5993597250421656,0.14669511401518
12,0.8260646845482855,0.8099244203028994,0.7286562842366027,0.69032284690072
34602,0.673092130911217,0.5831302190995835,0.3153637432661175,0.945024064580
83,0.8243504472493495,0.7302217177572492,0.3931471294940152,0.682540478767 15
8844084,0.39699216220747413,0.3648224702077987,0.23692399389672347,0.3316168
9,0.15631697070886086,0.6409655280987577,0.09696567096542474,0.5005793174308
72,0.7379509473975921,0.46618159112755697,0.1790316374415838,0.6457250433510
398,0.3604745051415539,0.1899361364575609,0.7975140452964858,0.510570408728
,0.6712708252291325,0.7017382738772572,0.6775458051837758,0.1199529417309698
,0.27064394198176567,0.556464637348822,0.5469288759854769,0.3120640446520206
08122963480667267,0.3586215819535914,0.47508987286676807,0.8023960305536325,
682,0.9925817954877634,0.9113391900786307,0.9224342836561245,0.8614180460955
,0.15620150793059928,0.020934117826733267,0.0727349477786684,0.1373000327155
9,0.988420993963481,0.315773151249496,0.28060081323791597,0.9523867523987246
772799325664473,0.05688139777764656,0.34993785357071405,0.32882261784860933
031,0.7938181039080396,0.7665273162216272,0.7824258122127878,0.662313738730 7
.6594229620819976,0.7021074541137926,0.9925146201619713,0.751825145956681 2,0
.5778070502510847,0.33135221873631704,0.24067051475715306,0.811946234637883
7312,0.8661015453778347,0.675662902207073,0.8743073341370806,0.945749702225 9
5,0.28943952161948794,0.39593722619594984,0.8306227604317913,0.3129195480288
768121248487704,0.7562537914224343,0.22465739764176662,0.858506454551554 5,0.3
0.6193899693665019,0.4541836627367061,0.3993325801992226,0.131686903436207 0
6,0.4111904758694911,0.41227972783166555,0.4096728692925191,0.705040906353 59
69,0.427486454090268,0.934729915488301,0.9782206685265583,0.7419777295139859
58,0.3302846675020844,0.25087909989070023,0.18089190137889832,0.677723241201
,0.564231816684536,0.12044733999656543,0.799157971104782,0.252314736135153,0
58,0.850574528015116,0.5845431132594582,0.3341927141731099,0.873043147015203 4
01678,0.20152837800631285,0.9693218122841789,0.7308703457761604,0.168858463 3
699473,0.934433038753441 4,0.37783921372718665,0.42001100429566285,0.05363479
90876537482297,0.06203676362878152,0.57835976311654,0.7174649906119122,0.381

3232732126800302,0.33930526730956745,0.8326117733261311,0.4754757152327337,0
5561906926300116,0.5059316543195584,0.8041655649838841,0.8490980199586761,0.
35,0.7795167014240635,0.1619230066322137,0.8664231835397002,0.38339714105825
,0.01620039733946721,0.658102218056492,0.05961732534465902,0.168278588507712
33,0.8183804247795352,0.9977493890102006,0.6920505530397448,0.83644219246986
0.04013885908536652,0.4517394759814438,0.13998737672176287,0.54890963164007
522,0.8899133763628625,0.35472855673021264,0.415818568018411,0.18919579239035
3,0.9919627472386433,0.40070416683446664,0.5485884150546885,0.72038399858644
92,0.2940674621767778,0.11514617839520114,0.40273948474683074,0.5111136384895
,0.21031855628579366,0.8832736014262238,0.5196116758030075,0.60431298224031
5,0.7653633638580407,0.414825655521352,0.9965263087986191,0.460898338206581
414,0.2530263409202532,0.3872744114248413,0.13521731371285473,0.828708983818
623831722035,0.6596902729304743,0.6754560453364256,0.8295627156830498,0.3137
04,0.10284259215747671,0.4420334209311235,0.3960009663856152,0.7093888218243
195,0.12595519637935826,0.648233468807846,0.13300979890732878,0.105401377396
736,0.9791515551006156,0.061446290130787506,0.15873676337430953,0.8388538619
75735,0.6738388982700046,0.8137233437222274,0.9492731062456169,0.67101298053
,0.8468872244289528,0.9405039528794026,0.879601496711762,0.6970241660042995,
,0.324329377351518,0.648765386159386,0.9070419604150218,0.36298284040387163,
192,0.6764837495206044,0.9545659288588108,0.9915327758273865,0.879279556157
42418273085972,0.1514323143699633,0.20831539000851795,0.7253716002348575,0.
03150120122947,0.4774161894803537,0.35543933736406175,0.11259046652919447,0.
53,0.6825324595377216,0.6018067339454473,0.6692190299248667,0.40777803440616
3,0.5753591731290986,0.5817366474211867,0.48380253986064725,0.88638036196014
32,0.42906236930266595,0.40146177908904324,0.4183895333783335,0.04719670169
,0.27592269438054684,0.3380844093662614,0.35781124933559305,0.96141661964204
1,0.768581330913202,0.1268240225504188,0.5937806319434522,0.6791625683195628,
3,0.2262724300031862,0.3917424505622782,0.575593428640082,0.6313759776433766
764,0.6923543061544142,0.16266081510548513,0.16145783406424008,0.27055925648
0.23333282264932664,0.7343656764922184,0.47173983751843906,0.24879178873917
4338,0.6253301699544539,0.20883093549458998,0.5534717762098499,0.94205821948
1894,0.7470782671290289,0.05856530528453385,0.5702869387313905,0.29827245423
2187138889065392,0.5523661378172343,0.2101071929123518,0.7755052967189994,0.
,0.05994330086130828,0.5952297327194016,0.7422713922522868,0.393121798207079
20662,0.13097374299417408,0.8719130612805055,0.025199094821069634,0.13925407
8619098,0.6059969672710255,0.5769816124176819,0.35483185554316266,0.01071529
077,0.8494464062257355,0.3454808537234457,0.7802126485356335,0.5442573370152
838228,0.04499682123542437,0.8030118815263205,0.5638929210554023,0.975778818
79,0.7341512590416462,0.5402788931874211,0.26116243251086024,0.369136193809
9,0.3413186459500521,0.07928028463119174,0.6949531193430477,0.357192333993044
.4785283699195225,0.5658505603132467,0.5673909441560734,0.3440916178494421,0
45,0.9288595498455656,0.9080185454216624,0.7962436094563872,0.520543837304858
431391,0.33197150473584547,0.39631262549822055,0.7350251861898234,0.44066488
91,0.46533693393948605,0.29632580189980007,0.6101079220362347,0.87991462672
.013682414760681105,0.14346945701802005,0.488177675310814,0.505520791723490
8,0.08159872000483837,0.15144552193535732,0.1331821256675234,0.5077578381885
072,0.258591591134784,0.4327714843418189,0.26133301788538377,0.1383715947205
027852240821074403,0.5956798241615295,0.41909947291708527,0.982739540910386
.631375326048251,0.08061540516960175,0.5992992870063887,0.24997289086882968,
03,0.4263983568016225,0.9395685890251657,0.5137078552153048,0.07336406745960
172224,0.548145202728022,0.7551336174451959,0.2881845259595295,0.66314624897
013,0.17464718589632955,0.5866239182836627,0.006464357789710551,0.5953663701
1,0.2959320294366161,0.8284386709389908,0.49623922190081393,0.41514565399274

1,0.6638244748527251,0.08018675681211329,0.2856259885622431,0.42219223388067
0.9653027236303998,0.4768388777331244,0.7346083007105351,0.7850639210464312,
84,0.0504167736022092,0.6300931268616634,0.02394626527048349,0.1957126400996
0157,0.8903843208552661,0.8286529061182892,0.5848278113057909,0.820253318944
0.5768812013112085,0.7834695600492677,0.9414742375060654,0.6487471138950182,
5231,0.5639195667556237,0.27706924971339963,0.1744092671114995,0.41896977377
35605,0.5002785352077603,0.9408801695592043,0.47164949075059537,0.4580286165
79,0.06939408590418339,0.12434584143087035,0.09142199181713817,0.25664720718
6,0.08987685173616067,0.8751336186211521,0.6256819122613526,0.75211433269697
8197475,0.6009120056590861,0.9709402496046186,0.5505316805962256,0.512874742
4,0.3409585714355583,0.17715352322262556,0.40736552421283234,0.320854542749
46,0.9172680665776807,0.7220660642037853,0.5213770214737412,0.59605011576095
0425,0.40659053735947226,0.04028551877998464,0.8965746584690102,0.995734452
5976,0.1432814587026410,0.4054228251391549,0.1962943023110670,0.4352622279
459791,0.714718833502036,0.516283299197674,0.02256451170534723,0.920691400
07,0.29335342144100984,0.5806452259096341,0.861664884699101,0.34501683992456
9672,0.5254703073558932,0.9359372482485595,0.5771170823879065,0.05473270692
7,0.69783278550517,0.6722506872941947,0.8906584554618424,0.2042421063684288
188,0.9002641112020704,0.481277951219237,0.5969452234131726,0.418695143327684
0.7921911948016761,0.8096750362658893,0.8101610899597025,0.219634928594569,0
5763610678727241,0.9505328209544089,0.42967336335514794,0.00022703821825553
21,0.6796331823951436,0.022066148682290843,0.7498599312882688,0.91500153774
513271,0.9459246440320865,0.9825212722560387,0.913041466889856,0.84387941123
29582409749653604,0.08613213906368034,0.5721229637222063,0.7422922314869471
08901,0.0011120941961874076,0.8143867792687614,0.18078515056237954,0.1678145
4,0.2719545593981987,0.2803347525514125,0.2687976150301981,0.1203635762099
28,0.217891379626744,0.13217965775387175,0.19864457689740578,0.067604749472
411,0.6618345851139982,0.4398556397138349,0.24685702759573291,0.7240291887
496,0.633711599765529,0.6439565794476135,0.3064154493510364,0.9288079606878
5934613827047238,0.380951845023475,0.5551337867531885,0.430535820913037,0.48
44,0.0162434432094537,0.05456540828269896,0.5879916294638328,0.11914067199604
5,0.7287780544506475,0.5994611396587166,0.4264997472418611,0.51239282073210
3,0.3235304124854915,0.9024032761106747,0.6154207798208354,0.3488319074586301
,0.6650559890066311,0.3832153659472517,0.08157492561136592,0.37482492434999
79965,0.5565784285021533,0.21633277763082703,0.08778823705815997,0.621330624
27309254279072,0.4425773459328154,0.17182946035765845,0.7187181132622024,0.34
0.13457626710410775,0.04836271104878087,0.5180774980403448,0.110603598873160
38284,0.09441889766048095,0.8204875844802748,0.21399467869831357,0.035590230
940106,0.8315183331122236,0.8263408109501867,0.28328440349257733,0.99433030
3,0.918397542081558,0.5880004634296284,0.40031283617299407,0.232728348915551
384,0.6497880467642996,0.3531028889383895,0.8118661240103798,0.0538305249084
566,0.10343411486048337,0.7997833644457834,0.013862135912860118,0.18987532909
565878,0.4020272168882407,0.5546870247883672,0.6493788202366224,0.0389578463
8862,0.7289392001051572,0.8259323085500162,0.6688765310406853,0.391810894617
0641,0.7798208840100681,0.6314587925830206,0.7986557803566365,0.80240178445
38,0.1182186222910886,0.7844054732127973,0.9327529390603132,0.34124444975772
.071884538241587e-05,0.59856299321776,0.020137848949289827,0.444183406878366
5,0.712137045880605,0.4141410682156662,0.15378370551321618,0.675586773567438
.35659617598620563,0.9579458599261376,0.886171294055993,0.5088940700698802,0
9812,0.25448133161826025,0.5411138248788447,0.45851856218701537,0.86144568602
4,0.01289746978952544,0.6050895185405384,0.5645906389334635,0.86603567504575
848,0.5402994067614997,0.22048763941474925,0.6630272037231474,0.009910695491
33,0.8511507354259817,0.6256507894272765,0.6790666044956374,0.77804630648319

4126,0.9576316462725879,0.5716076494829438,0.9317404033165082,0.460733600241
,0.5657449506132892,0.18483839794702495,0.9969494174116696,0.98884054130366
0104,0.5144613277398942,0.059587097353143714,0.7201574609309402,0.483232075
179,0.0850563854892411,0.6043593197147711,0.2872212092051508,0.986895945388
54516,0.5488728015425998,0.7636922693503205,0.8797524022943436,0.754569361
3799790789653946,0.5234013709220082,0.04922091454076161,0.24860488461330632
472,0.6071352808835769,0.2267726253196457,0.2310808528744962,0.5383949792869
73,0.3887263478765467,0.6665219766930349,0.8973857174637149,0.99213563947858
,0.24029912345036675,0.08010572660960225,0.25619151443935495,0.857530607751
21,0.0949652752283765,0.4418337048019546,0.22065105962174847,0.097366893791
,0.31491844458889573,0.1633667583984202,0.13812529314024724,0.70637199680999
30547,0.09664483428775228,0.18445905105988336,0.8591117576469165,0.345579585
95,0.17689695082398393,0.20229394915820365,0.5000582591910867,0.006982261880
873361585795459,0.3867070570439779,0.6748932030541548,0.4034809690269996,0.8
083,0.4441035569066186,0.051150205620397626,0.23906638407502967,0.2347544217
48,0.5318717665050166,0.3977934409290982,0.7580404210112165,0.81886927052047
0.8736185287372153,0.5117074471147008,0.7600055449620985,0.3373306088457655,
.9955689004906878,0.4832942849663252,0.31259802490830013,0.8275236939538171,
7643,0.5827144493446815,0.38278410040304056,0.41068263301028285,0.000597938
,0.8124738210441717,0.8391466080707525,0.20969027743020086,0.30781915002611
.32694102433667527,0.1453266924205041,0.9765610233229043,0.24116521430776
57439574472911,0.505696743781374,0.6456025770584116,0.23356736106861098,0.03
4027037209945602,0.062041416956554296,0.9345438807155155,0.0681866197654627,
19,0.6725072626736491,0.07115567887790974,0.30338554362913317,0.187124270410
52625,0.6821916892550988,0.5738658496770963,0.7818909129968897,0.555549569
58,0.3152394346494508,0.566018424887821,0.765858643884742,0.2913610728377445,
,0.1332099241366287,0.878033512844457,0.6916556518665415,0.417394782329147
255,0.6323962564655086,0.5578736756600732,0.9656389752668004,0.376866880347
,0.12832586218876452,0.9597910204048502,0.39355533610631555,0.87590492499989
6,0.5784925082293301,0.04869484839992977,0.1307173235897573,0.90529899901051
22897,0.693422128056309,0.09800768478074107,0.6717088592440608,0.49295697165
,0.7006114963944516,0.04406586157038139,0.7293098004772062,0.330155950315604
7,0.7534721106595967,0.18527685033354002,0.5750605734588147,0.106800704002
4,0.8728594215656645,0.5412701109416781,0.20681789313339627,0.953402879382
49995899196478844,0.645572181427428,0.5044559416693554,0.06022694490029745,0
584,0.7302491895295712,0.0455851579486688,0.3121897365691294,0.297650277568
1428926,0.6191727353658367,0.9459708898455993,0.42746652816668906,0.29806620
8663993979768256,0.842165376650381,0.6452185758446639,0.3204012861402149,0.8
4866,0.025689644869408168,0.2966124821570931,0.7452147426908345,0.164286843
8,0.28454138567693377,0.07718753022646796,0.23222469090134312,0.78316143092
2329,0.4430337063376194,0.16988707321905727,0.40532536064695335,0.122497861
312,0.6171059444286563,0.12694441375497267,0.2691615709916292,0.634906659153

86912,0.12384602670581746,0.3936355202774421,0.3044904243540304,0.2439953023
,0.07053563759295656,0.4734356593750214,0.9829468795547008,0.966734227535108
094295036858765,0.8545473932021803,0.6427774134799279,0.9581381198044961,0.1
5767,0.19012876631870568,0.34000438605989214,0.7176761577934996,0.24763164307
737669,0.8212501545597043,0.8696496848121713,0.9766176373150092,0.3480519554
).5446308711071358,0.08813443097669149,0.3772017824576387,0.895555740873885,0
077856519608769 6,0.7767984372086307,0.8018401684528059,0.9666222208961075,0.
92259,0.9554481914380675,0.8475476330547363,0.43453096330449537,0.4434391028
613,0.24696116699189252,0.18181765283733875,0.8695336718149798,0.32099704564
2946247,0.8596453453526065,0.43034653237220044,0.18065754131130318,0.5260940
3633,0.16881000578456418,0.16549977466365562,0.9476861770642796,0.6877692063
80335,0.8539428821418529,0.7066041289942178,0.21877170841087967,0.2734745153
83,0.013228812905293985,0.5353477680616567,0.3262205069809315,0.736473174619
092871,0.5117804565694807,0.635323346708321,0.7561922341067805,0.05165735711
26,0.7694425794215568,0.19648696695621115,0.3937551415654067,0.04929449276441
17672814,0.9347955174927833,0.21174586205241952,0.6082113767664467,0.3862877
91684,0.909202136206487,0.04149732550990637,0.4445735010694285,0.35347319206
958965,0.8196248695476449,0.3220675351957297,0.7423699370897818,0.3156332153
7,0.8881913496613041,0.559744661333675,0.22833125236861085,0.49101976266891,0
2445739,0.1992196858370765,0.8579349139973288,0.05845798723687701,0.3207335
.28418429571172876,0.6669274474586807,0.29992939747306624,0.1922579184680919
503848735,0.2889685249210585,0.43539632339975143,0.47430866486843026,0.30946
154,0.3770539310016988,0.9531201143941378,0.16802005219267857,0.974904272810
29,0.3933206703851887,0.719245200575831 9,0.3547972896563887,0.4111134169948486
5688,0.544555324654675 5,0.93016520286255,0.3999830182935685,0.4447127768583 35
71,0.1598797633664413 9,0.5275879732721657,0.05739184368827355,0.576544989951
4910783396,0.6912466637859893,0.2589042747931396,0.5833072481842045,0.983340
313,0.1675665393045065,0.05282668636472198 6,0.884343427971287 5,0.24160127643
536,0.3103084821045475 3,0.7260848514838505,0.15174291295305242,0.68128211053
5036,0.503345130606154,0.12130299800553412,0.597808013401514,0.7159846935417
2,0.7964405615234773,0.3027751282719672,0.6647392049108463,0.607324416793065
4,0.7394692332387273,0.5324709078838974,0.41933468041928834,0.0885783396366 4
,0.6455343564105223,0.5644165877797477,0.70109758736427,0.6768722758275765,0.
174,0.15577326058017016,0.600583196987136,0.41073154349101204,0.064094711036
57023,0.5470453456155777,0.16625533034970064,0.5046372879774349,0.5961426495
,0.08744650179988545,0.37973740482164875,0.007353044554426 98,0.739581177221 7
,0.37736317679665077,0.6171942271169667,0.6917201141346888,0.210969890619950
2,0.4135165302899591 6,0.9698188646337847,0.6613426789447244,0.8416220075427 7
).03981143944575383,0.7276311376996887,0.03840087732634456,0.030353805533004
4,0.2739880994699718,0.9226037570939578,0.3682501073951331,0.843812863658306
)301,0.9556494672664894,0.762212058314177,0.9790318407916674,0.4603290506427 6
226,0.9536326078296895,0.5917169688924004,0.4207365625686168,0.2175879399316
3523270542525393,0.19202336913634455,0.5019454051318417,0.133778144993968,0.
4,0.043100108948324056,0.666685651892513,0.9099301581597186,0.9533071675823 7
)27,0.17909493932818155,0.6233795659599071,0.7172618758657564,0.0726655875811
,0.3922177934335401 3,0.6024691704572664,0.5820701311242236,0.1990934199359 25
3204,0.9477796979692298,0.48978586798824075,0.7978046813410725,0.3478223474 3
.14119906353718492,0.5286566039892756,0.8643662664559733,0.6202348489611205,
4,0.7635158436682122,0.33444252711810407,0.45688807173726687,0.5448010852760 9
70395,0.10868253937469208,0.5193126926423032,0.4751083649318354,0.1256434924
152083084,0.9812575773912768,0.19755590567847825,0.3860509356518358,0.289727
616499109135,0.8051743973603768,0.9667061400184606,0.23875177108602408,0.675

.775234538899056,0.1857769546987078,0.6971662542903504,0.21354285449034138,0
3425910828111318,0.08496628988946642,0.0832230662080723,0.41114433647998705,
895,0.6276688771588087,0.43624602635715737,0.8629596233130185,0.704031452591
82,0.19201608474003673,0.6583074395060619,0.4805599408362681,0.2174529322172
97,0.14602101929696254,0.43771682493354136,0.06875390642248747,0.3666788797
17,0.9380641395681313,0.2767541319513086,0.549717255163311,0.3909174268411622
549,0.9176472779044335,0.5581711838904407,0.4171642789757021,0.97645652943541
58,0.8874689402128824,0.34700233541219994,0.8777494048626249,0.9546663568790
005,0.6254334501665935,0.9328676901266331,0.20399460360938737,0.800137582752
3,0.8082682112162782,0.9226451396035721,0.8264646094921829,0.241658920819185
,0.06903693691137491,0.5023718199720955,0.5576886911854527,0.06803731409166
064,0.33478904189067915,0.3278839940182585,0.05461030321346905,0.31690413772
031247571562,0.7371324139566391,0.9718453013254846,0.26679454943383085,0.61
862,0.6356453219526133,0.03671294348306664,0.5126152441031999,0.505925800146
46227,0.3559577734706405,0.47476900604365124,0.2908768542315825,0.777727910
040689,0.1485067816436808,0.33632339427033353,0.4694068419892581,0.858322938
29098,0.5964670754135437,0.9205358851025547,0.857857783517489,0.84486408541
.8143839206690815,0.012003027272682543,0.3049031243850635,0.359675561223067
.3821234944599732,0.5528148757183491,0.19267639648670032,0.6872343434606829
067,0.1322687150984817,0.740663581066626,0.12907124693088146,0.312834197524
9668819731282305,0.48501798752404124,0.29785939839021125,0.060905543235182
23748330516938398,0.08540303016272166,0.7800480870400435,0.7015652656029423
267,0.2998975929945362,0.9724613900279022,0.4716263651952617,0.0098275525697
17,0.6427950705063091,0.5180104313760979,0.22748160369353998,0.409182587458
378995,0.600945484537719,0.6141862443190114,0.1661849996097492,0.3293792693
95,0.6960798615670392,0.23660978270750732,0.3327294765103791,0.6933775385188
0.36250198453151317,0.4834980851068038,0.9399243728857088,0.766245472760378
,0.8239976518799783,0.42914949195832186,0.3430267125900244,0.304094635971283
79914,0.20408766115576027,0.07489584200198374,0.9881240759546354,0.8116270144
707,0.46904074751815394,0.10616035354746722,0.4852304557653562,0.29391676050
85955,0.8009387215414303,0.8374725196187746,0.17916391829015088,0.4463263599
412274,0.18015864773261925,0.23981995220659025,0.8911400917935094,0.72701852
10986311374078295,0.19495827149863054,0.4603553731348269,0.6929202292247886
15,0.8836839676879897,0.5049114512501393,0.7096713435045244,0.9873684723122,
078470398,0.3429296627591959,0.7687471313625129,0.9837115832091518,0.2251610
769535895,0.683870124909032,0.06194651857898792,0.050175350319974,0.66231791
508,0.02534860426393848,0.5768079196382558,0.09669455978756714,0.60968190827
5489327,0.6900468884094496,0.11921044284057336,0.7204567414627562,0.91509845
045,0.3161013115469705,0.03646105082561846,0.281825003295472,0.15636214164487
484,0.05574085358744174,0.05274395216221739,0.18430139695009906,0.7517981635
.609812761548117,0.8341641082084413,0.039361879819729495,0.5682686162687683,
34,0.24080381607317325,0.11797248362806156,0.021846711986201806,0.2746658697
342429355,0.38680724205188,0.04515136441229195,0.7744856031197909,0.47443491
0145,0.08058081228651359,0.4378998871429123,0.13443342736955,0.3337765970697
3,0.14716325109494532,0.8439934719420248,0.4677206159711509,0.57931340278846
688,0.29797723549584376,0.26240837648930193,0.605896815179483,0.889000610814
566,0.08975950056015713,0.4217126175259164,0.42835180056884625,0.56930498441
,0.9108262646863152,0.03992253256375122,0.027106652336233106,0.6317224183265
0.006047043183978418,0.4490785141348759,0.7835015025879088,0.51874784711470
267,0.4838602188643114,0.5776130806371337,0.010513224541653488,0.05116550644
52088,0.12324745813255145,0.5709070537521448,0.761889894377484,0.23285855687
568727,0.30767908947794853,0.33242669217436516,0.8933044937881227,0.49200172
57,0.9260595598182655,0.3155331806631355,0.4328399210571723,0.310235283744

806,0.16169455327398685,0.10662361893659789,0.5355424730977982,0.18176359776
).9658323197183326,0.3667983206462684,0.47698339261765377,0.5041328419184383
4906,0.2057943769894217,0.09949466887296465,0.3892086186234388,0.32280979344
3897,0.7415661084668296,0.7665959447120516,0.8874245353703603,0.258107643173
0.5466772648973117,0.9662742977481859,0.8939335106699905,0.4639854174403808,
95244,0.8844787275967592,0.9700744566667069,0.3358163646415345,0.13328425808
838686,0.5539932555031309,0.8650710993753938,0.615069754014698,0.92774573307
60958,0.5345592191563087,0.6007921493396191,0.9269075621458641,0.86770209791
48,0.1954580003683999,0.7008656972438799,0.9620584989413515,0.70762058574053
3052678,0.16622936205165872,0.28520580268041407,0.5089590404220475,0.1062138
436,0.9164891511155142,0.6307704201726206,0.4528248052843268,0.7758772544696
)2,0.32783819885003407,0.45564954943987745,0.2786943159285258,0.807957328109
051323,0.37208225496289593,0.4820173397769325,0.95887187319041,0.76776461771
55655384,0.7598236584283884,0.757776560375158,0.14281430447286736,0.11396588
786196,0.33882348242280735,0.28191835559725165,0.579829576047725,0.643907957
61,0.9520624466390027,0.3180396116945199,0.2825502675186389,0.83432479574939
985886,0.7379277689988003,0.9246417911286366,0.2790082903477966,0.5773534012
,0.59742924257342,0.056224294850972245,0.40496858737618846,0.646407375228089
42,0.8808951605447525,0.4856342984133404,0.9987929358156545,0.45836347942630
.04960590834948275,0.9128008004078617,0.054966306839822066,0.528707319884052
75,0.25706269218920874,0.610363007818157,0.5785418182035315,0.03437685835172
5272,0.46503633422090995,0.5464070742121321,0.08257215803527718,0.6389630219
)929,0.22697853947152102,0.2497441792293552,0.15452082999051897,0.0814472128
,0.7439051873021519,0.34327548906444827,0.4395629417728987,0.435014594340223
9564602863,0.9192080310537252,0.8520484158046929,0.5104601313464413,0.292655
506,0.4237701199309234,0.5592181317684581,0.258792408364524,0.545427950211713
8315,0.34213260011189495,0.5147421937567799,0.3164571308744162,0.1334163355
9212,0.9615507321375663,0.09957971354002892,0.08696702025593306,0.6916434019
53,0.44485243282754083,0.01602815645512312,0.3032197838442505,0.795011536928
90484004492832,0.9636851886870491,0.9665584393805441,0.1633544497431565,0.67
4816,0.35164987234190537,0.37698616728684164,0.9447073816724817,0.1043130873
5,0.9095484730121384,0.6032130620995043,0.334878290136985,0.14551548278735915
3,0.5689554832818507,0.07971507318728499,0.5708539362787616,0.8771275618985
7,0.7621745871816447,0.6831142857230466,0.9095953911109443,0.5281892617563562
1557073,0.8118067183508864,0.9325667087059609,0.0061838226408966745,0.467260
-18490419172582,0.5756652529974197,0.8106835703375919,0.10671181910235428,0.8
)85,0.43369433132038004,0.12611532237541057,0.948369552788919,0.6495560067569
306476386,0.8484717744062646,0.5817215274734767,0.09132573433355706,0.867999
5857149,0.36325535167581025,0.7663472131046524,0.16144768603292103,0.8281858
33,0.7939351334518513,0.40641653657401866,0.9607463027713554,0.6260547089808
59076,0.9409923225744371,0.8798377479414679,0.5375401436403463,0.075278716651
9591998,0.623210373068007,0.6486829533573645,0.014196634471723701,0.112397027
4846422,0.7416272874518889,0.8992489182892025,0.25763199876352916,0.04930898
17167,0.8050973120411349,0.6235053112473372,0.4499436742688596,0.2574792164
3243,0.8450396488966425,0.13128797200482423,0.6929089110247677,0.13566146716
)6,0.39505333785679597,0.3096917111051891,0.7331603781367996,0.2600480402524
55,0.05126345917599817,0.20179494825245692,0.9954215129254561,0.385058055718
8,0.4018762712367395,0.9088264977168623,0.287511199383759,0.9429827840711357
.6737562297840898,0.7604000672874827,0.5382201018684752,0.6103760597816921,0
213242,0.4503503857807928,0.676398368910006,0.533323547859605,0.834973785682
65,0.7489631508603986,0.3009663196316217,0.4056452594472375,0.710351500712333
153643,0.3569824294686639,0.18404477205021352,0.1691618938424202,0.112503130
92,0.6347441411040295,0.7564510879889118,0.9774565631838426,0.73592040637394

67387,0.18641980485397458,0.47415794587907967,0.8533171866665861,0.275098881
1,0.7493694366449569,0.22578955215062912,0.4983330282942372,0.639107846994706
37543515,0.5456959688130258,0.6166219781652751,0.6032454226840298,0.340961973
975,0.20406438854660058,0.040333852138451975,0.2272610535596623,0.99767226326
057366,0.29191117349780726,0.3261702541282535,0.5437870703917469,0.2635514794
,0.7486087085818324,0.46855699459113465,0.6949701331214758,0.279130470774269
577,0.4826012356615207,0.14795833410940584,0.5151670283865888,0.930672255562
27,0.2160314403930722,0.9846544775908396,0.9902851705849413,0.02464440463632
9237,0.9672711903702976,0.20909736458961425,0.6113416562477262,0.69668898787
211,0.7151250198168879,0.13035313004326554,0.37097371259216905,0.72532639073
61,0.9571348325397961,0.2035348738868964,0.7721520276158381,0.83637857741039
87081306,0.4271050453603432,0.7690611331597242,0.17046251158164205,0.9744393
348117,0.47510034069172047,0.4255056592718428,0.38451581370626653,0.9756019
979552161850192,0.565415939752538,0.34695895037758584,0.0652767575664639,0.7
249713,0.5250493402453846,0.021009156986360744,0.43180709510777726,0.8218393
15,0.7072789119056672,0.034431308573314556,0.7817798259287263,0.64630993365
0.25217287749552997,0.44582554982701617,0.48417303216340746,0.64734213437309
0.44672646870264054,0.8166552432016235,0.7940144502659627,0.1283499266146948
347953085,0.12344986581035511,0.8852388787075652,0.5764889031398659,0.650187
15,0.20598003587696168,0.08670656759564022,0.34206943465755535,0.9824825806
,0.000943415267631087,0.5383631021010228,0.8099787436834724,0.9118185695394
926505164866023,0.9132296862792705,0.23828967055309158,0.4391306608048505,0
,0.9772838231769293,0.38797275439788637,0.2243829797997955,0.039947973286094
50173,0.2423536951367623,0.8295658314283686,0.5045029240548358,0.36229349000
56199,0.663310709504002,0.1336956543994362,0.2608492459620889,0.82639031160
,0.8390996987996115,0.9353944896677987,0.009087034951216322,0.70149910146516
,0.5506604592365643,0.7514233175313026,0.6482975272988015,0.45934365559395
885,0.1531445000839562,0.9413343927869017,0.511093821472398,0.05510153515883
46,0.7281164373361664,0.6766172335525065,0.4455178144931612,0.42689472782330
55,0.600251410099583,0.363403931999427,0.9669685146183153,0.469024939717883
586005,0.7312505893473751,0.9381811725247224,0.5711991839629137,0.9285555328
,0.7697466228570872,0.2755667763091785,0.8634315777893845,0.64108610358215
363,0.9754103676847451,0.33217252206173253,0.13945023620516017,0.31165291665
5,0.57356328002671,0.7008084180017403,0.5692064631294277,0.5253182644507214,
.3416411126906018,0.7653162995253863,0.8679796267309029,0.06605530782216007,
8044,0.6484645569002371,0.9294034838491538,0.583105757279898,0.9725682356497
117000415,0.06844070924262613,0.2048024090562095,0.7180141781270015,0.360059
973973183215863,0.797998035219224,0.3720435678122074,0.4778673549131358,0.79
7240928,0.11938101133819679,0.7385460533969634,0.09244951703706661,0.3552839
6972,0.32784283788581825,0.06379455625359787,0.4579002951270714,0.68251173011
6598922,0.8157453806261209,0.38722314121020696,0.3944793908388575,0.66223215
1628,0.5973123783307118,0.2827654570600986,0.8670601823789473,0.71532231052

[illegible]95490886009,0.9111205013919575,15.373408822678767,1871251.6498327148

7684142266,0.43804407590538375,19.92569275878817,3223220.305859041

9837382146,0.7416841289918231,38.485464636684966,4694992.065770977

[illegible]943208276,0.25232274426374524,28.030818465285424,3238193.7011923436

901930726,0.5028755200655849,17.176312239143957,1995626.2673679679
