# Supplementary material for: Risk-indexed artificial neural network for predicting duration and cost of irrigation canal-lining projects using survey-based calibration and python validation
Source: Sci Rep. 2025 Nov 17;15:40316. doi: 10.1038/s41598-025-24125-1 (PMC12623735; doi:10.1038/s41598-025-24125-1)
Supplement: Supplementary file 2 — Supplementary Information 2. [file 41598_2025_24125_MOESM2_ESM.pdf]

```python
import tkinter as tk
from tkinter import ttk, messagebox
import numpy as np
import matplotlib.pyplot as plt
from matplotlib.backends.backend_tkagg import FigureCanvasTkAgg
from sklearn.neural_network import MLPRegressor
from sklearn.preprocessing import StandardScaler, MinMaxScaler
from sklearn.model_selection import train_test_split
from sklearn.metrics import r2_score
from sklearn.base import clone
import warnings

class EICLPPredictor:
    def __init__(self, root):
        self.root = root
        self.root.title("EICLP Time & Cost Predictor with ANN - Corrected")
        self.root.geometry("1200x1000")

        # Initialize model and scalers
        self.model = None
        self.input_scaler = StandardScaler()
        self.output_scaler = MinMaxScaler()
        self.trained = False
        self.loss_curve = []

        self.initialize_variables()
        self.create_widgets()
        self.setup_plots()
        self.train_ann_model()

    def initialize_variables(self):
        """Initialize all class variables and default values"""
        self.default_values = {
            'canal_length': 2620,
            'stone_thickness': 0.30,
            'concrete_thickness': 0.10,
            'concrete_area': 0.9275,
            'bottom_width': 2.0,
            'water_depth': 1.5,
            'side_slope': 1.0,
            'radius': 1.25,
            'top_width': 3.0
        }

        self.risk_factors = [
            {"name": "Concrete pouring works", "weight": 0.1140, "value": 0.0},
            {"name": "Water turn rotation", "weight": 0.1124, "value": 0.0},
            {"name": "Labor productivity", "weight": 0.1083, "value": 0.0},
            {"name": "Canal cross-section area", "weight": 0.1065, "value":
0.0},
            {"name": "Owner payment policy", "weight": 0.1063, "value": 0.0},
            {"name": "Cost estimate accuracy", "weight": 0.0964, "value": 0.0},
            {"name": "Equipment costs", "weight": 0.0960, "value": 0.0},
            {"name": "Survey efficiency", "weight": 0.0952, "value": 0.0},
```

```
            {"name": "Concrete mixer productivity", "weight": 0.0945, "value":
0.0},
            {"name": "Stone construction works", "weight": 0.0940, "value":
0.0},
            {"name": "Equipment efficiency", "weight": 0.0936, "value": 0.0},
            {"name": "Labor skills shortage", "weight": 0.0906, "value": 0.0},
            {"name": "Stone lining area", "weight": 0.0898, "value": 0.0},
            {"name": "Material costs", "weight": 0.0885, "value": 0.0},
            {"name": "Surveying works", "weight": 0.0860, "value": 0.0},
            {"name": "Financing sources", "weight": 0.0838, "value": 0.0},
            {"name": "Environmental impact", "weight": 0.0833, "value": 0.0},
            {"name": "Local job creation", "weight": 0.0806, "value": 0.0},
            # العاملان المضافان
            {"name": "Canal lining length", "weight": 0.0850, "value": 0.0},  #
CD4
            {"name": "Concrete lining area", "weight": 0.0870, "value": 0.0}   #
CD6
        ]

    def create_widgets(self):
        """Create all GUI components"""
        main_frame = ttk.Frame(self.root)
        main_frame.pack(fill=tk.BOTH, expand=True, padx=10, pady=10)

        # Project Parameters Frame
        param_frame = ttk.LabelFrame(main_frame, text="Project Parameters")
        param_frame.grid(row=0, column=0, padx=5, pady=5, sticky="nsew")

        # Project Parameters
        ttk.Label(param_frame, text="Canal Length (m):").grid(row=0, column=0,
sticky="e")
        self.canal_length = ttk.Entry(param_frame)
        self.canal_length.insert(0, str(self.default_values['canal_length']))
        self.canal_length.grid(row=0, column=1)

        ttk.Label(param_frame, text="Stone Thickness (m):").grid(row=1,
column=0, sticky="e")
        self.stone_thickness = ttk.Entry(param_frame)
        self.stone_thickness.insert(0,
str(self.default_values['stone_thickness']))
        self.stone_thickness.grid(row=1, column=1)

        ttk.Label(param_frame, text="Concrete Thickness (m):").grid(row=2,
column=0, sticky="e")
        self.concrete_thickness = ttk.Entry(param_frame)
        self.concrete_thickness.insert(0,
str(self.default_values['concrete_thickness']))
        self.concrete_thickness.grid(row=2, column=1)

        ttk.Label(param_frame, text="Concrete Area (m²):").grid(row=3, column=0,
sticky="e")
        self.concrete_area = ttk.Entry(param_frame)
        self.concrete_area.insert(0, str(self.default_values['concrete_area']))
        self.concrete_area.grid(row=3, column=1)
```

```
        # Geometry Parameters
        ttk.Label(param_frame, text="Bottom Width (m):").grid(row=4, column=0,
sticky="e")
        self.bottom_width = ttk.Entry(param_frame)
        self.bottom_width.insert(0, str(self.default_values['bottom_width']))
        self.bottom_width.grid(row=4, column=1)

        ttk.Label(param_frame, text="Water Depth (m):").grid(row=5, column=0,
sticky="e")
        self.water_depth = ttk.Entry(param_frame)
        self.water_depth.insert(0, str(self.default_values['water_depth']))
        self.water_depth.grid(row=5, column=1)

        # الخطأ كان هنا - تم تصحيح tttk إلى ttk
        ttk.Label(param_frame, text="Side Slope (H:V):").grid(row=6, column=0,
sticky="e")
        self.side_slope = ttk.Entry(param_frame)
        self.side_slope.insert(0, str(self.default_values['side_slope']))
        self.side_slope.grid(row=6, column=1)

        ttk.Label(param_frame, text="Radius (m):").grid(row=7, column=0,
sticky="e")
        self.radius = ttk.Entry(param_frame)
        self.radius.insert(0, str(self.default_values['radius']))
        self.radius.grid(row=7, column=1)

        ttk.Label(param_frame, text="Top Width (m):").grid(row=8, column=0,
sticky="e")
        self.top_width = ttk.Entry(param_frame)
        self.top_width.insert(0, str(self.default_values['top_width']))
        self.top_width.grid(row=8, column=1)

        # Risk Factors Frame with Scrollbar
        risk_frame = ttk.LabelFrame(main_frame, text="Risk Factors (0.0 - 1.0)")
        risk_frame.grid(row=0, column=1, padx=5, pady=5, sticky="nsew")

        risk_canvas = tk.Canvas(risk_frame)
        scrollbar = ttk.Scrollbar(risk_frame, orient="vertical",
command=risk_canvas.yview)
        scrollable_frame = ttk.Frame(risk_canvas)

        scrollable_frame.bind("<Configure>", lambda e:
risk_canvas.configure(scrollregion=risk_canvas.bbox("all")))

        risk_canvas.create_window((0, 0), window=scrollable_frame, anchor="nw")
        risk_canvas.configure(yscrollcommand=scrollbar.set)

        for i, factor in enumerate(self.risk_factors):
            ttk.Label(scrollable_frame, text=factor["name"]).grid(row=i,
column=0, sticky="e", padx=5, pady=2)
            slider = ttk.Scale(scrollable_frame, from_=0.0, to=1.0, value=0.0)
            slider.grid(row=i, column=1, sticky="ew", padx=5, pady=2)
            setattr(self, f"risk_{i}", slider)
```

```
        risk_canvas.pack(side="left", fill="both", expand=True)
        scrollbar.pack(side="right", fill="y")

        # Results Frame
        result_frame = ttk.LabelFrame(main_frame, text="Results")
        result_frame.grid(row=1, column=0, columnspan=2, padx=5, pady=5,
sticky="ew")

        ttk.Label(result_frame, text="Estimated Duration:").grid(row=0,
column=0, sticky="e")
        self.duration_result = ttk.Label(result_frame, text="")
        self.duration_result.grid(row=0, column=1, sticky="w")

        ttk.Label(result_frame, text="Estimated Cost:").grid(row=1, column=0,
sticky="e")
        self.cost_result = ttk.Label(result_frame, text="")
        self.cost_result.grid(row=1, column=1, sticky="w")

        ttk.Label(result_frame, text="Model Status:").grid(row=2, column=0,
sticky="e")
        self.model_status = ttk.Label(result_frame, text="Not trained",
foreground="red")
        self.model_status.grid(row=2, column=1, sticky="w")

        # Buttons
        button_frame = ttk.Frame(main_frame)
        button_frame.grid(row=2, column=0, columnspan=2, pady=10)

        ttk.Button(button_frame, text="Calculate",
command=self.calculate).pack(side=tk.LEFT, padx=5)
        ttk.Button(button_frame, text="Reset",
command=self.reset).pack(side=tk.LEFT, padx=5)
        ttk.Button(button_frame, text="Retrain Model",
command=self.train_ann_model).pack(side=tk.LEFT, padx=5)
        ttk.Button(button_frame, text="Show Loss Curve",
command=self.show_loss_curve).pack(side=tk.LEFT, padx=5)

    def setup_plots(self):
        """Initialize the visualizations"""
        # Create a frame for plots
        plot_frame = ttk.Frame(self.root)
        plot_frame.pack(side=tk.BOTTOM, fill=tk.BOTH, expand=True)

        # Main results plot
        self.figure1 = plt.Figure(figsize=(8, 4), dpi=100)
        self.plot1 = self.figure1.add_subplot(111)
        self.canvas1 = FigureCanvasTkAgg(self.figure1, master=plot_frame)
        self.canvas1.get_tk_widget().pack(side=tk.LEFT, fill=tk.BOTH,
expand=True)

        # Loss curve plot
        self.figure2 = plt.Figure(figsize=(8, 4), dpi=100)
        self.plot2 = self.figure2.add_subplot(111)
```

```
        self.canvas2 = FigureCanvasTkAgg(self.figure2, master=plot_frame)
        # التصحيح هنا: تغيير RRIGHT إلى RIGHT
        self.canvas2.get_tk_widget().pack(side=tk.RIGHT, fill=tk.BOTH,
expand=True)

    def generate_training_data(self, num_samples=5000):
        """Generate realistic training data"""
        np.random.seed(42)

        X = np.zeros((num_samples, 9 + len(self.risk_factors)))

        # Realistic parameter ranges
        X[:, 0] = np.random.uniform(1000, 5000, num_samples)  # canal_length
        X[:, 1] = np.random.uniform(0.2, 0.5, num_samples)    # stone_thickness
        X[:, 2] = np.random.uniform(0.05, 0.15, num_samples)  #
concrete_thickness
        X[:, 3] = np.random.uniform(0.5, 1.5, num_samples)    # concrete_area
        X[:, 4] = np.random.uniform(1.5, 3.0, num_samples)    # bottom_width
        X[:, 5] = np.random.uniform(1.0, 2.0, num_samples)    # water_depth
        X[:, 6] = np.random.uniform(0.5, 1.5, num_samples)    # side_slope
        X[:, 7] = np.random.uniform(0.5, 2.0, num_samples)    # radius
        X[:, 8] = np.random.uniform(2.0, 4.0, num_samples)    # top_width

        for i in range(len(self.risk_factors)):
            X[:, 9 + i] = np.random.uniform(0, 1, num_samples)

        y = np.zeros((num_samples, 2))

        for i in range(num_samples):
            base_duration = X[i, 0] / 1000 * np.random.uniform(6, 12)  # 6-12
months per km

            stone_vol = X[i, 3]/X[i, 2] * X[i, 1] * X[i, 0]
            concrete_vol = X[i, 3] * X[i, 0]
            base_cost = stone_vol * 70 + concrete_vol * 1350

            risk_factor = np.sum(X[i, 9:] * np.array([f['weight'] for f in
self.risk_factors])) / sum(f['weight'] for f in self.risk_factors)
            y[i, 0] = base_duration * (1 + risk_factor * 0.3)
            y[i, 1] = base_cost * (1 + risk_factor * 0.2)

        return X, y

    def train_ann_model(self):
        """Train the ANN model with loss tracking"""
        try:
            X, y = self.generate_training_data()
            X_train, X_test, y_train, y_test = train_test_split(X, y,
test_size=0.2, random_state=42)

            self.input_scaler.fit(X_train)
            self.output_scaler.fit(y_train)

            X_train_scaled = self.input_scaler.transform(X_train)
```

```
            y_train_scaled = self.output_scaler.transform(y_train)

            # Train the model normally and use the built-in loss_curve
            self.model = MLPRegressor(
                hidden_layer_sizes=(128, 64, 32),
                activation='relu',
                solver='adam',
                max_iter=1000,
                batch_size=32,
                learning_rate_init=0.001,
                early_stopping=True,
                random_state=42,
                verbose=False
            )

            self.model.fit(X_train_scaled, y_train_scaled)

            # Get the loss curve if available
            if hasattr(self.model, 'loss_curve_'):
                self.loss_curve = self.model.loss_curve_
            else:
                # If no loss_curve available, create a simple one
                self.loss_curve = [self.model.loss_] if hasattr(self.model,
'loss_') else [1.0]

            train_pred =
self.output_scaler.inverse_transform(self.model.predict(X_train_scaled))
            test_pred =
self.output_scaler.inverse_transform(self.model.predict(self.input_scaler.transf
orm(X_test)))

            train_r2 = r2_score(y_train, train_pred)
            test_r2 = r2_score(y_test, test_pred)

            self.trained = True
            self.model_status.config(text=f"Trained (Train R²: {train_r2:.2f},
Test R²: {test_r2:.2f})", foreground="green")

            # Update loss curve plot
            self.update_loss_curve()

        except Exception as e:
            self.trained = False
            self.model_status.config(text="Training failed", foreground="red")
            messagebox.showerror("Training Error", f"Model training
failed:\n{str(e)}")

    def update_loss_curve(self):
        """Update the loss curve visualization"""
        if not self.loss_curve:
            return

        self.plot2.clear()
        iterations = range(1, len(self.loss_curve) + 1)
```

```
        self.plot2.plot(iterations, self.loss_curve, 'b-', linewidth=2)
        self.plot2.set_title('Training Loss Curve')
        self.plot2.set_xlabel('Iteration')
        self.plot2.set_ylabel('Loss')
        self.plot2.grid(True)
        self.plot2.set_yscale('log')  # Use log scale for better visualization

        # Add final loss value annotation
        if len(self.loss_curve) > 0:
            final_loss = self.loss_curve[-1]
            self.plot2.annotate(f'Final Loss: {final_loss:.4f}',
                              xy=(len(self.loss_curve), final_loss),
                              xytext=(len(self.loss_curve)*0.7, final_loss*10),
                              arrowprops=dict(facecolor='black', shrink=0.05),
                              fontsize=10)

        self.canvas2.draw()

    def show_loss_curve(self):
        """Show loss curve in a separate window"""
        if not self.loss_curve:
            messagebox.showinfo("Info", "No loss curve available. Please train
the model first.")
            return

        loss_window = tk.Toplevel(self.root)
        loss_window.title("Training Loss Curve")
        loss_window.geometry("800x600")

        fig = plt.Figure(figsize=(10, 6), dpi=100)
        ax = fig.add_subplot(111)
        iterations = range(1, len(self.loss_curve) + 1)
        ax.plot(iterations, self.loss_curve, 'b-', linewidth=2)
        ax.set_title('ANN Training Loss Curve', fontsize=14)
        ax.set_xlabel('Iteration', fontsize=12)
        ax.set_ylabel('Loss (Log Scale)', fontsize=12)
        ax.grid(True)
        ax.set_yscale('log')

        # Add statistics
        if len(self.loss_curve) > 0:
            min_loss = min(self.loss_curve)
            max_loss = max(self.loss_curve)
            final_loss = self.loss_curve[-1]
            initial_loss = self.loss_curve[0] if len(self.loss_curve) > 0 else 0
            improvement = ((initial_loss - final_loss) / initial_loss) * 100 if
initial_loss > 0 else 0

            stats_text = f"""Loss Statistics:
            Initial Loss: {initial_loss:.4f}
            Final Loss: {final_loss:.4f}
            Minimum Loss: {min_loss:.4f}
            Maximum Loss: {max_loss:.4f}
            Improvement: {improvement:.1f}%
```

```
            Iterations: {len(self.loss_curve)}"""

            ax.text(0.02, 0.98, stats_text, transform=ax.transAxes, fontsize=10,
                    verticalalignment='top', bbox=dict(boxstyle='round',
facecolor='wheat', alpha=0.8))

        canvas = FigureCanvasTkAgg(fig, master=loss_window)
        canvas.draw()
        canvas.get_tk_widget().pack(fill=tk.BOTH, expand=True)

    def calculate(self):
        """Make prediction"""
        if not self.trained:
            messagebox.showerror("Error", "Model not trained yet!")
            return

        try:
            inputs = [
                float(self.canal_length.get()),
                float(self.stone_thickness.get()),
                float(self.concrete_thickness.get()),
                float(self.concrete_area.get()),
                float(self.bottom_width.get()),
                float(self.water_depth.get()),
                float(self.side_slope.get()),
                float(self.radius.get()),
                float(self.top_width.get())
            ]

            risks = [getattr(self, f"risk_{i}").get() for i in
range(len(self.risk_factors))]
            features = np.array([inputs + risks])

            features_scaled = self.input_scaler.transform(features)
            pred_scaled = self.model.predict(features_scaled)
            prediction = self.output_scaler.inverse_transform(pred_scaled)

            duration, cost = prediction[0]
            risk_factor = self.calculate_risk_factor()

            self.duration_result.config(text=f"{duration:.2f} months")
            self.cost_result.config(text=f"EGP {cost:,.2f}")

            self.update_visualization(
                base_duration=duration / (1 + risk_factor * 0.3),
                adjusted_duration=duration,
                base_cost=cost / (1 + risk_factor * 0.2) / 1e6,
                adjusted_cost=cost / 1e6,
                risk_factor=risk_factor
            )

        except ValueError:
            messagebox.showerror("Input Error", "Please check your input
values!")
```

```
        except Exception as e:
            messagebox.showerror("Prediction Error", f"Prediction
failed:\n{str(e)}")

    def calculate_risk_factor(self):
        """Calculate weighted risk impact"""
        total_weighted_risk = 0.0
        total_weights = 0.0

        for i, factor in enumerate(self.risk_factors):
            risk_value = getattr(self, f"risk_{i}").get()
            total_weighted_risk += risk_value * factor["weight"]
            total_weights += factor["weight"]

        return total_weighted_risk / total_weights if total_weights > 0 else 0.0

    def update_visualization(self, base_duration, adjusted_duration, base_cost,
adjusted_cost, risk_factor):
        """Update the results visualization"""
        self.plot1.clear()

        categories = ['Base Duration', 'Adjusted Duration', 'Base Cost',
'Adjusted Cost']
        values = [base_duration, adjusted_duration, base_cost, adjusted_cost]
        colors = ['#1f77b4', '#ff7f0e', '#2ca02c', '#d62728']

        bars = self.plot1.bar(categories, values, color=colors)

        for bar in bars:
            height = bar.get_height()
            unit = ' months' if 'Duration' in bar.get_label() else 'M EGP'
            self.plot1.text(bar.get_x() + bar.get_width()/2., height,
                         f'{height:.2f}{unit}',
                         ha='center', va='bottom')

        self.plot1.set_title(f'Project Analysis | Risk Factor:
{risk_factor:.2f}')
        self.plot1.set_ylabel('Months / Million EGP')
        self.plot1.grid(True, axis='y')

        self.canvas1.draw()

    def reset(self):
        """Reset all inputs to default values"""
        self.canal_length.delete(0, tk.END)
        self.canal_length.insert(0, str(self.default_values['canal_length']))

        self.stone_thickness.delete(0, tk.END)
        self.stone_thickness.insert(0,
str(self.default_values['stone_thickness']))

        self.concrete_thickness.delete(0, tk.END)
        self.concrete_thickness.insert(0,
str(self.default_values['concrete_thickness']))
```

```python
        self.concrete_area.delete(0, tk.END)
        self.concrete_area.insert(0, str(self.default_values['concrete_area']))

        self.bottom_width.delete(0, tk.END)
        self.bottom_width.insert(0, str(self.default_values['bottom_width']))

        self.water_depth.delete(0, tk.END)
        self.water_depth.insert(0, str(self.default_values['water_depth']))

        self.side_slope.delete(0, tk.END)
        self.side_slope.insert(0, str(self.default_values['side_slope']))

        self.radius.delete(0, tk.END)
        self.radius.insert(0, str(self.default_values['radius']))

        self.top_width.delete(0, tk.END)
        self.top_width.insert(0, str(self.default_values['top_width']))

        for i in range(len(self.risk_factors)):
            getattr(self, f"risk_{i}").set(0.0)

        self.duration_result.config(text="")
        self.cost_result.config(text="")
        self.plot1.clear()
        self.canvas1.draw()

if __name__ == "__main__":
    root = tk.Tk()
    app = EICLPPredictor(root)
    root.mainloop()
```
