# Supplementary material for: Risk-indexed artificial neural network for predicting duration and cost of irrigation canal-lining projects using survey-based calibration and python validation
Source: Sci Rep. 2025 Nov 17;15:40316. doi: 10.1038/s41598-025-24125-1 (PMC12623735; doi:10.1038/s41598-025-24125-1)
Supplement: Supplementary file 3 — Supplementary Information 3. [file 41598_2025_24125_MOESM3_ESM.pdf]

EICLP Time & Cost Predictor (Tkinter App)

This repository contains the supplementary material for the article:

Dynamic Construction Site Layout Optimization Using Deep Reinforcement Learning with PPO

Contents

- `app.py`: Tkinter-based desktop application for predicting project duration and cost using an ANN model.

- `requirements.txt`: List of Python dependencies and versions used.

- `demo_dataset.csv`: Synthetic dataset generated for demonstration and reproducibility.

Requirements

- Python 3.13

- Install dependencies with:

  ```bash
  pip install -r requirements.txt
  ```

Usage

1. Launch the application:

   ```bash
   python app.py
   ```

2. Use the **Input interface (Figure 8a)** to enter project parameters and risk factor levels.

3. Click **Calculate** to generate predictions.

4. The **Output interface (Figure 8b)** displays predicted duration (months), cost (EGP), and uncertainty bands.

Reproducibility

- The demo dataset (` demo_dataset.csv`) can be used to reproduce figures reported in the article.

- The preprocessing pipeline (StandardScaler for inputs, MinMaxScaler for outputs) is consistent with model training.

- Versioning: v1.0.0 (Python 3.13, scikit-learn 1.4, numpy ≥1.25, matplotlib ≥3.7, pandas ≥2.0).

Notes

- Tkinter comes bundled with Python and does not require installation via pip.

- The application includes input validation, error handling, and loss curve visualization.
