# Supplementary material for: Risk-indexed artificial neural network for predicting duration and cost of irrigation canal-lining projects using survey-based calibration and python validation
Source: Sci Rep. 2025 Nov 17;15:40316. doi: 10.1038/s41598-025-24125-1 (PMC12623735; doi:10.1038/s41598-025-24125-1)
Supplement: Supplementary file 4 — Supplementary Information 4. [file 41598_2025_24125_MOESM4_ESM.pdf]

```
# requirements.txt
python==3.13
numpy>=1.25
pandas>=2.0
matplotlib>=3.7
scikit-learn==1.4
tkinter  # Comes bundled with Python, listed here for clarity
```
