# Supplementary material for: Risk-indexed artificial neural network for predicting duration and cost of irrigation canal-lining projects using survey-based calibration and python validation
Source: Sci Rep. 2025 Nov 17;15:40316. doi: 10.1038/s41598-025-24125-1 (PMC12623735; doi:10.1038/s41598-025-24125-1)
Supplement: Supplementary file 5 — Supplementary Information 5. [file 41598_2025_24125_MOESM5_ESM.pdf]

Table (S1). The 20 most important factors affecting the estimation of EICLPs' contingency (items map) [10]

Code	Name of factor	Group	Sub-Group	Frequency Index (FI)	Impact Index (II)	Final Index (F.I)	Relative Weight	Rank
CC7	Pouring works of plain concrete mortar consisting of (gravel, sand, cement, and water) for irrigation canals' beds, side slopes, and feet	Cost-Related Factors	Construction-Related Factors	0.3320	0.3433	0.1140	100.00%	1
TP5	Water turn rotation in irrigation canals (on and off turns)	Time-Related Factors	Project-Related Factors	0.3333	0.3373	0.1124	98.60%	2
TP10	Labor productivity participating in irrigation canals lining projects	Time-Related Factors	Project-Related Factors	0.3173	0.34 13	0.1083	95.00%	3
CD2	Area of irrigation canals' cross-section [Hydraulic cross-sectional area] (m^2)	Cost-Related Factors	Design-Related Factors	0.3267	0.3260	0.1065	93.42%	4
TO3	Owner's payment policy (such as Initial payment and invoices)	Time-Related Factors	Owner-Related Factors	0.3240	0.3280	0.1063	93.25%	5
CD6	Area of irrigation canals cross-section with plain concrete lining (m^2)	Cost-Related Factors	Design-Related Factors	0.3200	0.3273	0.1047	91.84%	6
CD4	Irrigation canals lining length (m run)	Cost-Related Factors	Design-Related Factors	0.3147	0.3113	0.0980	85.96%	7
FR1	Inaccurate project cost estimate	Risk-Related Factors	Financial Risk-Related Factors	0.3007	0.3207	0.0964	84.56%	8
CO5	Equipment expenses as (leased equipment, owned equipment, and maintenance and repairs)	Cost-Related Factors	Overheads-Related Factors	0.3187	0.3013	0.0960	84.21%	9
TP3	Efficiency of irrigation canals' surveying works that need to be lined	Time-Related Factors	Project-Related Factors	0.3180	0.2993	0.0952	83.51%	10
TP12	Productivity of plain concrete mixer in site used for pouring beds, side slopes, and feet for irrigation canals that required lining them	Time-Related Factors	Project-Related Factors	0.3060	0.3087	0.0945	82.89%	11
CC6	Construction works of rubble stones for irrigation canals' beds, side slopes, and feet	Cost-Related Factors	Construction-Related Factors	0.3167	0.2967	0.0940	82.46%	12
TP7	Equipment efficiency used in the execution of irrigation canals lining works	Time-Related Factors	Project-Related Factors	0.3067	0.3053	0.0936	82.11%	13

TR4	Lack of sufficiently skilled and trained labor	Risk-Related Factors	Technical Risk-Related Factors	0.3000	0.3020	0.0906	79.47%	14
CD5	Area of irrigation canals cross-section with rubble stone lining (m^2)	Cost-Related Factors	Design-Related Factors	0.2960	0.3033	0.0898	78.77%	15
FR2	High costs of materials and equipment needed for irrigation canals lining works	Risk-Related Factors	Financial Risk-Related Factors	0.2727	0.3247	0.0885	77.63%	16
CC3	Irrigation canals survey works such as (reshaping, levels' adjustments, ...)	Cost-Related Factors	Construction-Related Factors	0.3440	0.2500	0.0860	75.44%	17
TC1	Financing sources for irrigation canals lining projects	Time-Related Factors	Contractor-Related Factors	0.2720	0.3080	0.0838	73.51%	18
ER5	Effects of irrigation canals lining projects on plant and animal life	Risk-Related Factors	Environmental Risk-Related Factors	0.2840	0.2933	0.0833	73.07%	19
SR4	Create local jobs opportunities and improve the economic conditions of the region	Risk-Related Factors	Social Risk-Related Factors	0.2760	0.2920	0.0806	70.70%	20
