# Supplementary material for: Risk-indexed artificial neural network for predicting duration and cost of irrigation canal-lining projects using survey-based calibration and python validation
Source: Sci Rep. 2025 Nov 17;15:40316. doi: 10.1038/s41598-025-24125-1 (PMC12623735; doi:10.1038/s41598-025-24125-1)
Supplement: Supplementary file 6 — Supplementary Information 6. [file 41598_2025_24125_MOESM6_ESM.pdf]

Table (S2). The main groups affecting the estimation of EICLPs' contingency pre- and post-findings of ranking from the questionnaire [10]

Group No.	Group Name	Factors in each group pre-		Factors in each group post-	
		**Sum**	**Weight**	**Sum**	**Weight**
1	Cost-Related	25	26.88 %	8	40 %
2	Time-Related	30	32.26 %	7	35 %
3	Risk-Related	38	40.86 %	5	25 %
	**Total**	**93**	**100.00%**	**20**	**100.00%**
